# Computational Design of a PDZ Domain Peptide Inhibitor that Rescues CFTR Activity

**DOI:** 10.1371/journal.pcbi.1002477

**Published:** 2012-04-19

**Authors:** Kyle E. Roberts, Patrick R. Cushing, Prisca Boisguerin, Dean R. Madden, Bruce R. Donald

**Affiliations:** 1 Department of Computer Science, Duke University, Durham, North Carolina, United States of America; 2 Department of Biochemistry, Dartmouth Medical School, Hanover, New Hampshire, United States of America; 3 Institute for Medical Immunology, Charite Universitätsmedizin, Berlin, Germany; 4 Department of Biochemistry, Duke University Medical Center, Durham, North Carolina, United States of America; Consiglio Nazionale delle Ricerche, Italy

## Abstract

The cystic fibrosis transmembrane conductance regulator (CFTR) is an epithelial chloride channel mutated in patients with cystic fibrosis (CF). The most prevalent CFTR mutation, ΔF508, blocks folding in the endoplasmic reticulum. Recent work has shown that some ΔF508-CFTR channel activity can be recovered by pharmaceutical modulators (“potentiators” and “correctors”), but ΔF508-CFTR can still be rapidly degraded via a lysosomal pathway involving the CFTR-associated ligand (CAL), which binds CFTR via a PDZ interaction domain. We present a study that goes from theory, to new structure-based computational design algorithms, to computational predictions, to biochemical testing and ultimately to epithelial-cell validation of novel, effective CAL PDZ inhibitors (called “stabilizers”) that rescue ΔF508-CFTR activity. To design the “stabilizers”, we extended our structural ensemble-based computational protein redesign algorithm 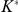 to encompass protein-protein and protein-peptide interactions. The computational predictions achieved high accuracy: all of the top-predicted peptide inhibitors bound well to CAL. Furthermore, when compared to state-of-the-art CAL inhibitors, our design methodology achieved higher affinity and increased binding efficiency. The designed inhibitor with the highest affinity for CAL (kCAL01) binds six-fold more tightly than the previous best hexamer (iCAL35), and 170-fold more tightly than the CFTR C-terminus. We show that kCAL01 has physiological activity and can rescue chloride efflux in CF patient-derived airway epithelial cells. Since stabilizers address a different cellular CF defect from potentiators and correctors, our inhibitors provide an additional therapeutic pathway that can be used in conjunction with current methods.

## Author Summary

Cystic fibrosis (CF) is an inherited disease that causes the body to produce thick mucus that clogs the lungs and obstructs the breakdown and absorption of food. The cystic fibrosis transmembrane conductance regulator (CFTR) is mutated in CF patients, and the most common mutation causes three defects in CFTR: misfolding, decreased function, and rapid degradation. Drugs are currently being studied to correct the first two CFTR defects, but the problem of rapid degradation remains. Recently, key protein-protein interactions have been discovered that implicate the protein CAL in CFTR degradation. Here we have developed new computational protein design algorithms and used them to successfully predict peptide inhibitors of the CAL-CFTR interface. Our algorithm uses a structural ensemble-based evaluation of protein sequences and conformations to calculate accurate predictions of protein-peptide binding affinities. The algorithm is general and can be applied to a wide variety of protein-protein interface designs. All of our designed inhibitors bound CAL with high affinity. We tested our top binding peptide and observed that the inhibitor could successfully rescue CFTR function in CF patient-derived epithelial cells. Our designed inhibitors provide a novel therapeutic path which could be used in combination with existing CF therapeutics for additive benefit.

## Introduction

**Figure 1:**
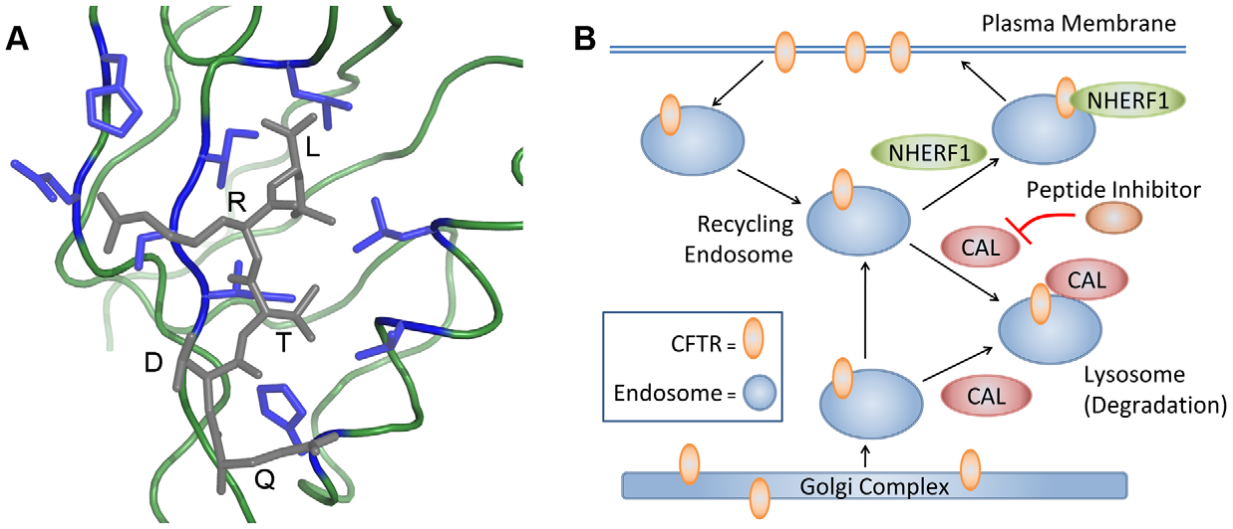
(A) Structural model of the CAL PDZ domain (green and blue) bound to a CFTR C-terminus mimic (gray) used as input for computational designs (PDB id: 2LOB). Residues shown in blue were modeled as flexible during the design search. (B) Model of the CFTR trafficking pathway with PDZ domain containing proteins NHERF1 and CAL. CAL is associated with lysosomal degradation of CFTR, while NHERF1 is associated with insertion of CFTR into the cell membrane.

Protein-peptide interactions (PPIs) are vital for cell signaling, protein trafficking and localization, gene expression, and many other biological functions. The PDZ (PSD-95, discs large, zonula occludens-1) family of proteins forms PPIs that play crucial physiological roles, including synapse formation [1] and epithelial cell polarity and proliferation [2]. The common PDZ structural core generally binds a specific sequence motif at the extreme C-terminus of its binding partner through 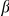-sheet interactions (Fig. 1A). Recently, key PPIs have been discovered linking the trafficking of the cystic fibrosis transmembrane conductance regulator (CFTR) to PDZ domain containing proteins [3] (Fig. 1B). Specifically, the PDZ domain of the CFTR-associated ligand (CAL) binds CFTR, targeting it for lysosomal degradation and reducing its half-life at the plasma membrane [4], [5].

CFTR is an epithelial chloride channel that is mutated in cystic fibrosis (CF) patients. The most common disease-associated mutation, ΔF508-CFTR, is a single amino acid deletion that causes CFTR misfolding and endoplasmic reticulum-associated (ER) degradation. There is now evidence that the ΔF508-CFTR loss of function can be pharmacologically improved through the use of “correctors” [6] and “potentiators” [7]. Correctors, such as corr-4a [6], [8], work by correcting the folding defect of CFTR and preventing ER retention of CFTR. Potentiators combat mutant CFTR gating defects and increase the flow of ions through CFTR channels present at the cellular membrane. Despite these interventions, the half-life of ΔF508-CFTR in the membrane is still reduced compared to that of the wild-type protein [9]. However, the CAL-mediated degradation of ΔF508-CFTR can be reduced by RNA interference or by mutagenesis of the CAL PDZ domain, suggesting that a competitive inhibitor of the CAL binding site could act as a CFTR “stabilizer” and thus ameliorate CF symptoms [3], [10]. Since stabilizers address a different underlying CF defect than correctors and potentiators, combined application can achieve additive rescue of ΔF508-CFTR activity [11].

Since PDZ domains have an inherent affinity for peptides, here we focus on the use of protein design methods to rationally design a competitive peptide inhibitor that could serve as a ΔF508-CFTR stabilizer. Indeed, the development of successful peptide inhibitor design tools would provide a means to target a wide variety of PPIs for both mechanistic and therapeutic applications. Several aspects of our new 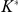 design algorithm (described below) are well suited to the requirements of this class of problems.

In general, structure-based computational protein design seeks amino-acid sequences that are compatible with a specific protein fold. Often, additional functional constraints are applied to the problem in order to design a protein with a given binding or catalytic activity. Because protein conformational space is large, design algorithms often assume a fixed backbone conformation and reduce side-chain configuration space by using discrete conformations called *rotamers* [12]–[15]. Thus, most current design methods try to solve the traditional design problem, which can be defined as: for a given *input model* (protein structure, rotamer library, and energy function), find the side chain rotamers that yield a single, global minimum energy conformation (GMEC) for the entire protein [16]–[34]. However, in reality, a protein in solution exists as a thermodynamic ensemble and not just a single low-energy structure [35]. Accounting for such ensembles can help find true native protein structures [36]–[39]. The design algorithm we present here, 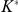, takes this into account by computing Boltzmann-weighted partition functions over structural molecular ensembles to find provably-accurate approximations to the binding constant for a protein complex [40], [41]. The value of this approach is reflected in previous applications of the 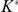 algorithm to design a switch in enzyme specificity for an enzyme in the non-ribosomal peptide synthetase pathway [40] and to predict resistance mutations for antibiotic targets [42].

As with the established 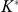 algorithm, most successful protein design studies have focused on protein/small molecule systems, since predicting PPI binding is more challenging than small molecule binding, due to PPIs' much larger, flexible, and energetically shallow binding surfaces. The methodologies that have been developed to study protein-protein interactions and, more specifically, PDZ domain interactions, can be divided into sequence- [43], [44] and structure-based [38], [45]–[49] methods. Sequence-based methods require a large amount of sequence and binding information for the protein family and do not provide direct structural information on the modeled interaction. Among the previous structure-based alternatives, most focus on finding the single GMEC conformation, although one study suggests that designing to a set of different backbone conformations can improve recovery of PDZ domain binding motifs [45]. In addition, only the work of Altman *et al.* [46] utilizes provable techniques, and none use both provable techniques and protein ensembles. In comparison, the 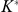 algorithm is more general, requiring only a starting template structure and preserving structural information on the modeled interaction. It also evaluates energy-weighted ensembles, employs provable guarantees for finding the optimal sequence, and uses the minimization aware dead-end elimination (minDEE) pruning criteria [16], [41] to permit continuous minimization of rotamers during the search. As a result, 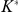 complements existing approaches while addressing some of their methodological limitations. Here we report the development of new extensions to the 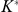 algorithm, enabling the software to design novel PPIs.

Using this new tool we designed high-affinity CAL PDZ inhibitors and validated them in both biochemical and cell-culture experiments. We present peptide array data which shows that CAL binds a specific sequence motif, but does not bind all sequences within that motif. Therefore, it is important that the 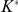 algorithm is able to differentiate the affinities of peptides that share the motif, rather than just separating motif from non-motif sequences. Overall, 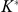 searched 2166 peptide inhibitor sequences within the CAL binding motif (approximately 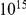 possible conformations) and generated top-ranked peptides that had up to a 170-fold improvement in binding to CAL compared to the wild-type CFTR sequence. The best binder was able to rescue ΔF508-CFTR function in human cells.

## Materials and Methods

### 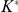 Algorithm


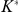
 computationally searches over peptide amino acid substitutions (mutations) for a given protein-peptide complex and assigns each candidate sequence a score, called a 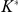 *score* [40], [41]. To compute the score for a given protein-peptide complex candidate sequence, 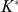 evaluates the low-energy conformations for the sequence and uses them to compute a Boltzmann-weighted partition function. Partition functions are computed for each protein binding partner using rotamer-based ensembles defined as 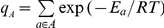, 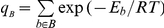, 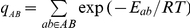 where 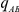 is the partition function for protein 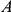 bound to protein 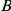, and 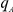 and 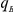 are the partition functions for the unbound proteins, 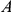 and 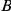. The 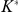 score is defined as the ratio of partition functions: 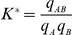, which is an approximation of the protein complex association constant, 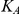 [41]. Candidate sequences are ranked based on their 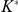 score, where sequences with a higher 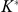 score are considered to have a higher affinity for the target protein.

**Figure 2:**
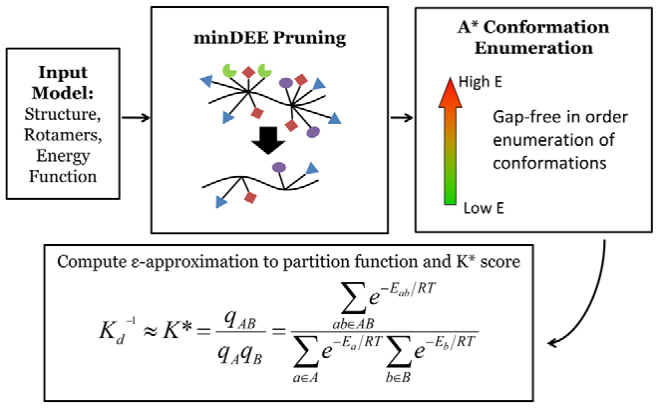
Overview of 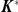 Algorithm. he 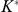 algorithm searches over protein sequences and conformations to find the protein complexes with the best binding constant. 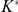 takes an *input model* composed of an initial protein structure, a rotamer library to search over side-chain conformations, and an energy function to evaluate conformations. Minimization-aware DEE (minDEE) prunes rotamers that are not part of the lowest energy conformations for a given sequence. The remaining conformations from minDEE are enumerated in order of increasing energy lower bounds using A*. Finally, the conformations are Boltzmann-weighted and used to compute partition functions and ultimately a 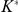 score for each sequence.

The 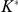 algorithm has been described previously [16], [40], [41]. Briefly, to calculate a partition function for a given sequence, 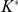 finds low energy conformations by performing a rotamer search as follows. First, 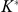 uses an enhanced version of dead-end elimination (DEE), minDEE [16], [41], [50], to prune side-chain rotamers that provably cannot be part of low-energy structures. Since rigid-rotamer DEE [34], [51] often eliminates rotamers and sequences that are involved in *bona fide* low-energy conformations [50], 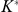 prunes rotamers using minDEE, which allows local side-chain rotamer minimization to relieve clashes that are incorrectly pruned by rigid rotamer design methods. In order for minDEE to account for minimization during the rotamer search, it computes energy lower bounds for each rotamer pair. The branch-and-bound algorithm 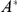 [30] is used to enumerate conformations in gap-free order of their minimum energy bounds. These conformations are minimized and their Boltzmann-weighted energy is incorporated into the partition function. The partition function is computed with respect to the input model (protein structure, energy function, and rotamer library), so the accuracy of the partition function is bounded by the accuracy of the input model. Refer to Fig. 2 to see the general framework for the 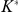 algorithm.

The energy minimization scheme that is used for both the energy lower bounds computation and the minimization of a full conformation is similar to previous descriptions [41]. The 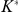 algorithm's minimization protocol separates a protein's degrees of freedom (DOF) into three categories: (1) backbone dihedrals (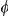 and 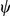 angles) (2) side-chain dihedrals (up to four 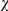 angles per side chain) and (3) rigid body rotation and translation (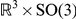). The minimization process holds the backbone dihedrals fixed while allowing the side-chain dihedral and rigid body DOF to minimize. The minimization over these DOF is performed using gradient descent. To prevent rotamers from minimizing from one rotamer to another, each side-chain dihedral was only allowed to move a maximum of 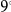 from its modal rotameric value.

### Extension of 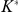 to Amino Acid Substitutions/Flexibility on Two Protein Strands


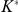
 relies on the mathematically provable guarantees of each of its steps (Fig. 2) to compute an accurate 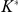 score. If we were to use heuristic steps to find the low energy conformations, it could not be guaranteed that all the low energy conformations are found and we would lose the ability to calculate a provably-good 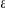-approximation (where 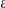 is user-defined) to each partition function for the design system. Because of the provable aspects of 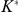, if 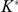 makes an errant prediction, we can be certain that it is due to an inaccuracy in the input model and not a problem (such as inadequate optimization) with our search algorithm. This makes it substantially easier to improve the model based on experimental feedback, as we show in Section S2 of Text S1.

Before applying 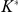 to PPI designs, we first had to ensure that the mathematical framework of 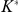 could be extended to cover larger systems. For large designs such as PPIs, the provable guarantees of 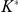 no longer hold as they did for small design systems. Specifically, the previous 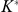 proofs [41] for intermutation pruning and guaranteeing the accuracy of the 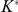 score, relied on properties of small molecule design systems that are not true for PPIs. We now show that it is possible to improve the 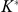 algorithm to maintain these critical provable guarantees. As a result, systems where both binding partners in the protein complex are flexible or mutable during the search can be accurately studied using 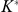.

*Intermutation pruning* uses computed partition functions to truncate the conformation enumeration process for candidate sequences when they will provably fail to achieve a 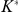 score close to the best 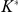 score. To show that an intermutation pruning criterion [41] exists for PPI design we seek a halting condition for the conformation enumeration such that we know we have an 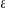-approximation to the bound partition function for a given protein complex. First we observe: 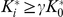, where 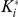 is the 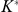 score of the current sequence, 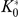 is the best score observed so far, and 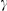 is a user-selected parameter. In the following lemma, 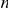 is the number of conformations in the search that remain to be computed, 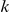 is the number of conformations that have been pruned from the search with DEE, 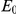 is the lower energy bound on all pruned conformations, 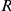 is the universal gas constant, and 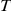 is the temperature. The full partition function for the protein-protein complex, and unbound proteins are 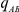, 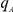, and 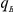 respectively, while 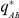, 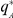, and 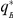 denote the current calculated value of the partition functions during the computational search.

#### Lemma 1

*If the lower bound* 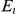 *on the minimized energy of the* 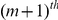 *conformation returned by* 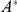 *satisfies* 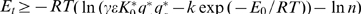 *, then the partition function computation can be halted, with* 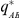 *guaranteed to be an* 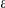 *-approximation to the true partition function,* 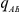 *, for a candidate sequence whose score* 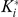 *satisfies* 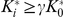 *.*

This lemma shows that even when designing for protein-protein interactions, there exists a sequence pruning criterion during the 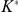 search.

Now we show that we can obtain a provable guarantee on the accuracy of the 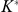 score for each protein conformation. Since both partition functions are 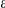-approximations, we no longer obtain an 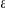-approximation to the 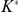 score but rather the following:

#### Lemma 2

*When amino acid substitutions (or flexible residues) are allowed on both strands in the computational design, the computed* 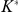 *score is a* 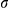 *-approximation to the actual* 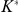 *score, where* 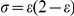 *.*

Since neither of the protein complex partition functions are calculated fully, the 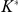 score approximation is a 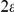-approximation as opposed to the 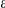-approximation for small molecule designs. This implies that we must compute better partition function approximations than before to maintain the same level of 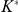 score approximation. Nevertheless, the fact that the 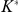 score can still be provably approximated, confers all the advantages of a provable algorithm as stated above. The proofs of Lemmas 1 and 2 are provided in Text S1.

### Computational Designs with 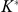


The previously-determined NMR structure of the CAL PDZ domain bound to the C-terminus of CFTR (PDB ID: 2LOB) was used to model the binding of CAL to CFTR. To prepare the protein complex for the computational design, the initial complex structure was obtained by molecular dynamics refinement of the NMR structure as described previously [52]. Hydrogens were added to the structure using Reduce [53]. The CFTR peptide in the NMR structure was truncated to the six most C-terminal amino acids. An acetyl group was modeled onto the N-terminus of the peptide using restrained molecular dynamics and minimization in which the N-terminus of the peptide was allowed to move, while the remainder of the protein complex was restrained using a harmonic potential [54]. The coordinates of this starting structure are provided as supporting information (Text S2).

An 8 Å shell around the peptide hexamer was used as the input structure to 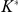. The CFTR C-terminal residues, VQDTRL, were mutated to the following residues during the design search: 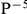 to W, 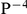 stayed fixed to Q, 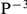 to all amino acids except Pro, 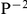 to T/S, 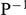 to all amino acids except Pro, and 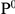 to I/L/V. In addition, the Probe program [55] was used to determine the side-chains on CAL that interact with the CFTR peptide mimic. The nine residues that interact with the peptide, as well as the two most N-terminal residues on the peptide, were allowed to be flexible during the design search (Fig. 1A). To explore the feasibility of our new algorithms, unless otherwise noted, full partition functions were not computed and a maximum of 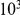 conformations were allowed to contribute to each partition function.

Rotamer values were taken from the Penultimate Rotamer Library modal values [14]. The energy function used to evaluate protein conformations has been previously described [40], [42]. The energy function, 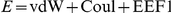, consists of a van der Waals term, a Coulombic electrostatics term, and an EEF1 implicit solvation term [56]. The EEF1 solvation term implicitly models water solvent during all of the computational designs. All design runs used the Amber98 [57] forcefield terms except for one prospective design run which used the Charmm19 [58] forcefield parameters.

### Training of Energy Function Weights

Previously-determined experimental binding constants [59] for 16 of CAL's natural ligands were used to train the energy function weight parameters (See Text S1 Section S2). 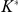 scores were computed for each of the natural ligands. For this training, the CAL-CFTR structure only included the four most C-terminal residues of the peptide inhibitor. A gradient descent method was used to optimize the correlation between the 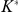 scores and the experimental 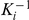 values. The final parameters chosen for the design runs are as follows: a van der Waals scaling of 0.9, a dielectric constant of 20, and a solvation scaling of 0.76.

### Peptide Array Comparison


**Figure 3:**
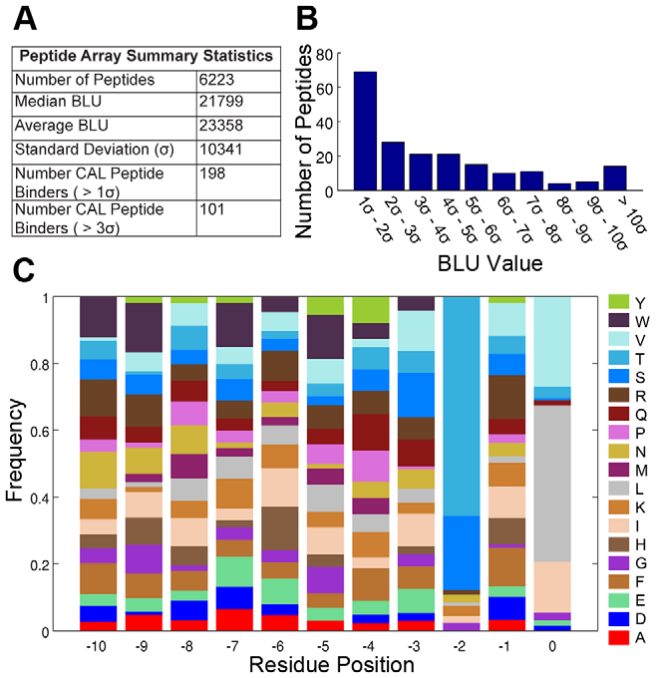
Summary of CAL peptide array. (A) Summary statistics for peptide array. Higher BLU (biochemical light unit) values indicate stronger protein binding to a peptide. (B) Distribution of the peptide BLU values from the peptide array in units of standard deviation above the mean (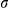). (C) Normalized amino acid frequencies for the top sequences that have a BLU value greater than 3 standard deviations from the average, which were considered as the peptides that bound CAL for the validation of 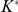 predictions. The frequency of each amino acid type for each residue position was normalized by the total number of occurrences of that amino acid in the array at the given residue position.

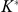
 was used to predict binding between the CAL PDZ domain and the HumLib set of 6223 human protein C-termini. The binding of the C-termini peptides to CAL was experimentally assessed using a peptide SPOT array [59], [60]. Due to experimental restrictions, all cysteines in the HumLib peptide set were replaced by serine in the peptide array. For consistency, all computational predictions compared to the array modeled serines in the place of cysteines. A summary of the peptide array data is presented in Fig. 3 while the complete binding results from the array are provided as **Supporting Information** (Table S1). The 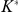 algorithm was used to evaluate 4-mer structural models of 6223 peptide-array sequences to verify the accuracy of the algorithm's predictions. To compare the array data with the 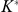 predictions, the quantitative array data, measured in biochemical light units (BLUs), was converted into a binary yes/no CAL binding event. In other words, by using a fixed cutoff value, each sequence from the array was classified as either a CAL binder or non-binder. The cutoff value was chosen as three standard deviations away from the average BLU value of the array. A receiver operating curve (ROC), which uses a floating cutoff to compare array data to 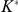 scores, was used to evaluate the ability of 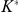 to predict the array binding data.

After the 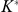 predictions were calculated, the binding of C-termini peptides to CAL was also experimentally assessed using an additional SPOT array. The profile library array (ProLib; Fig. S3 in Text S1) was designed based on the following motif: bbbb 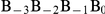 (B = permutation of a defined set of amino acids, b = mixture of 17 amino acids, without C, M and W). The defined set of amino acids were selected based on the HumLib results combined with substitutional analyses [60] with 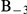 = A/C/D/E/F/I/K/L/M/N/Q/R/S/T/V/W/Y, 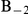 = S/T, 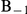 = A/C/D/E/F/I/K/L/M/N/Q/R/S/T/V/W/Y, 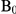 = I/L/V (Total number of peptides = 1734+22 internal control sequences). Incubation condition: 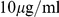 His-tagged CAL PDZ domain detected by anti-His (Sigma; 1∶2600)/anti-mouse-HRP (Calbiochem; 1∶2000) antibody sandwich.

### Prospective Computational Predictions


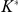
 was used to search over all peptide sequences within the CAL PDZ domain sequence motif (excluding prolines) to find new CAL peptide inhibitors. For computational efficiency the number of conformations enumerated by A* for each partition function was limited to 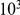 conformations. Two sets of peptides (promising designs and poorly ranked designs) were chosen to be experimentally validated.

In order to choose the most promising peptide inhibitors, a second 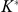 design was done where 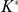 scores for the top 30 sequences were re-calculated with the number of enumerated conformations per partition function increased to 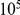. Several top-ranked sequences were chosen to be experimentally tested. First, the top 7 ranked sequences from the second run were chosen. In addition, two sequences that greatly increased in ranking from the first to second run (rank 29 to 9, and rank 28 to 11) were chosen as well. Finally, a 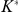 run was conducted using Charmm forcefield parameters instead of Amber parameters. Two sequences that scored high on both the Amber and Charmm runs were chosen to be experimentally tested as well (Table 1).

**Table 1:** Experimental validation of top-ranked 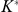 predictions.

Name	Sequence	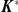 Ranking	Experimental
		(out of 2166)	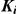 ( 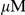 )
kCAL01	Ac-WQVTRV	9	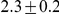 †
kCAL02	Ac-WQFTRL	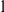 ‡	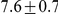 †
kCAL03	Ac-WQKTRL	2	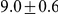 †
kCAL04	Ac-WQRTRL	5	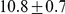 †
kCAL05	Ac-WQKTRI	4	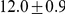 †
kCAL06	Ac-WQKTRV	1	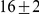
kCAL07	Ac-WQFTKL	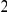 ‡	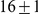
kCAL08	Ac-WQRTRI	7	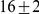
kCAL09	Ac-WQLTKL	11	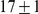
kCAL10	Ac-WQKTKL	6	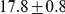
kCAL11	Ac-WQRTRV	3	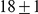

**†:** 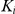 values with a binding affinity higher than the best previously known hexamer (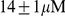). These sequences are shown in green in Fig. 5.

**‡:** Sequence rank obtained by ordering the quantity: 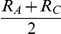, where 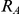 is the sequence rank from a design run using the Amber forcefield and 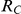 is the sequence rank from a run using the Charmm forcefield.

The poorly-ranked designs were chosen to minimize the sequence similarity among the set of poorly-ranked peptides (Table 2). First, the worst-ranked peptide was chosen and added to initialize the set of negative sequences. Next, sequences were successively chosen from the worst 200 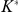 ranked sequences and added to the set in order to maximize the amino acid sequence diversity with all the sequences already in the set. The similarity between two sequences was determined using the PAM-30 similarity matrix [61]. In total 23 (eleven top-ranked and twelve poorly-ranked) K^*^-computed peptide inhibitor sequences were experimentally tested.

**Table 2:** Experimental validation of poorly-ranked 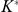 predictions.

Name	Sequence	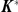 Ranking	Experimental
		(out of 2166)	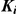 ( 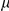 M)
kCAL20	Ac-WQYTMI	1981	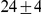
kCAL21	Ac-WQYTDL	2082	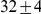
kCAL22	Ac-WQISWL	1973	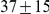
kCAL24	Ac-WQHTEV	1989	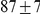
kCAL23	Ac-WQMTDI	1969	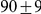
kCAL25	Ac-WQCSEI	2051	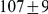
kCAL26	Ac-WQESEL	2095	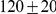
kCAL27	Ac-WQDTWI	2158	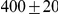
kCAL28	Ac-WQWSDV	2166	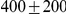
kCAL29	Ac-WQDSCV	2011	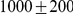
kCAL30	Ac-WQGSDV	2075	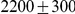
kCAL31	Ac-WQDSGI	1992	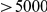

### Measuring Peptide Inhibitor Constants

The inhibitor dissociation constants of top- and poorly-ranked peptide sequences from the 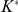 CAL-CFTR design were experimentally determined. As a control, the best known peptide hexamer was also retested. The corresponding N-terminally acetylated peptides were purchased from NEO BioScience (Cambridge, MA) and the 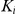 values for the peptides were detected using fluorescence polarization (FP), using the method previously described in [59]. Briefly, the CAL PDZ domain was incubated in FP buffer (25 mM Tris-HCl pH 8.5, 150 mM NaCl; supplemented to a final concentration of 0.1 mg/mL bovine IgG (Sigma) and 0.5 mM Thesit (Fluka)) with a labeled peptide of known binding affinity. Each peptide inhibitor was serially diluted and the protein-peptide mixture was added to each dilution. Finally, the amount of competitive inhibition was tracked using residual fluorescence polarization at temperatures between 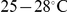. Each 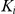 value is reported as an average of three FP experiments conducted on separate days along with the corresponding standard deviation.

### Measuring Chloride Flux

Ussing chamber experiments were performed as described previously [11]. Polarized monolayers of patient-derived bronchial epithelial cells, CFBE-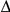
F cells (a generous gift of Dr. J.P. Clancy [62], [63]), were maintained in MEM with 2 mM l-glutamine, 10% fetal bovine serum, 50 units/mL penicillin, 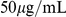 streptomycin, 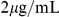 puromycin, 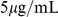 plasmocin, and 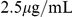 amphotericin B. Cells were grown at 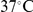 in 5% 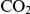. Twenty four hours before treatment the cells were moved to MEM with only penicillin and streptomycin. Peptides were dissolved in DMSO and diluted to 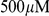 in PBS. Peptide solutions were applied to cells following incubation with BioPORTER delivery reagent (Sigma). The final DMSO concentration did not exceed 0.03%. Following a 3.5 hour incubation with peptide, short circuit currents (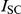) were monitored in Ussing chambers. Following treatment with amiloride, forskolin, and genistein, ΔF508-CFTR chloride flux was measured as the change in 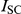 when the CFTR-specific inhibitor, 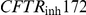 [64], [65], was applied to the cell monolayer. All measurements were performed at 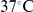.

## Results

We applied the 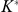 algorithm to the CAL-CFTR system to find a CAL PDZ peptide inhibitor that acts as a biologically active stabilizer of ΔF508-CFTR. First, we developed the ensemble-based computational structural design software 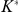 to design PPIs. To validate the design methodology, the predictions of the 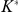 algorithm were compared with binding data of CAL binding human protein C-termini. The validation showed 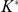 was able to enrich for peptide inhibitors. We then used 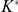 to prospectively find new peptide inhibitors of CAL. The top-scoring predicted sequences were experimentally validated and we determined that they all bind CAL with 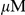 affinity. Next, additional binding data for peptide sequences that match the known CAL binding motif were collected and compared to the 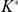 predictions. Finally, Ussing chamber experiments showed that the highest affinity designed peptide significantly rescues ΔF508-CFTR in bronchial epithelial cells.

### Validation of the 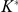 Algorithm

**Figure 4:**
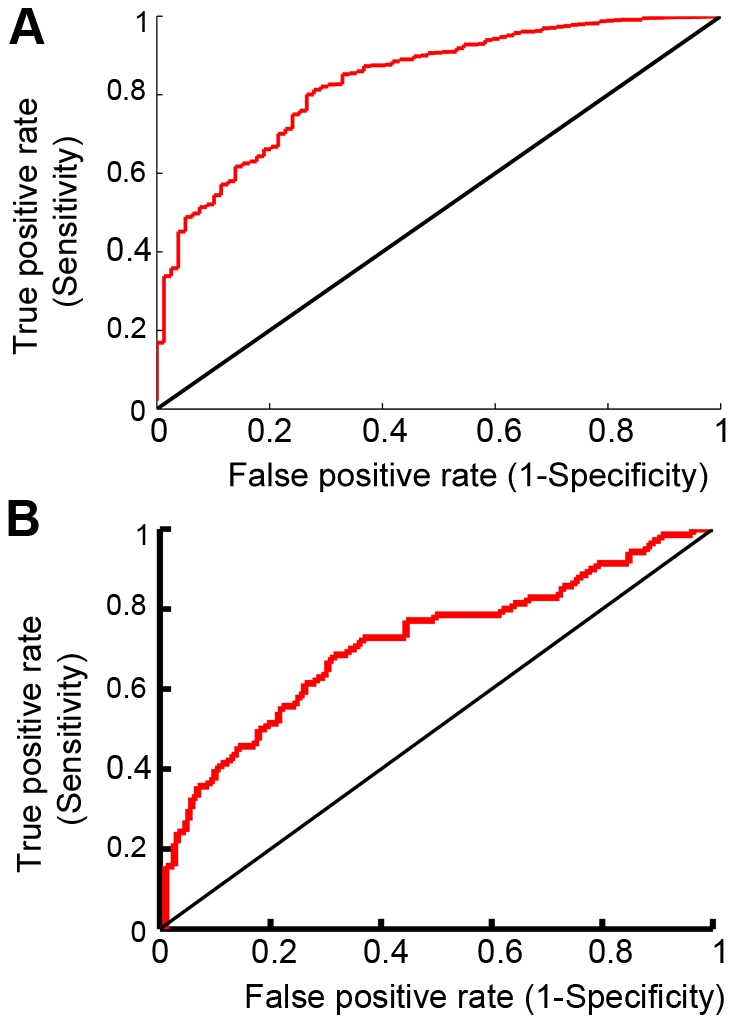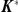 enriched for peptide sequences that bind the CAL PDZ domain. ROCs were calculated comparing 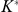 predictions to (A) the entire HumLib peptide array data set (AUC = 0.84) and (B) only sequences in the HumLib array that matched the CAL binding motif (AUC = 0.71).

To validate the 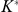 algorithm, we compared 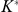 predictions for CAL peptide inhibitors against peptide array binding data. First, peptides from the 6223 peptide HumLib library were tested for CAL binding using a SPOT array [59]. The array was able to find over one hundred peptides that clearly bind the CAL PDZ domain (Fig. 3). Second, 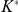 predictions were made for all of the peptide sequences in the HumLib library. Fig. 4A shows the resulting receiver operating curve (ROC) when comparing the 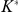 scores to the binding measurements (BLU values) of the peptide array. The ROC has an area under the curve (AUC) of 0.84 which shows that 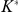 greatly enriches for peptides that bind CAL. Specifically, according to the peptide array, out of the top 30 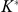 predicted sequences, 11 are expected to bind CAL. Notably, this is a 20-fold increase over the number of binders that would be expected to be found if the CAL binding peptides were distributed randomly within the 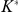 predictions.

To investigate the success of the algorithm in more detail, we evaluated the importance of the CAL binding motif in determining 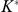 predictions. The amino acid frequencies from the top binding peptides of the HumLib library (Fig. 3C) and natural binding partners of CAL [59] reveal that the canonical sequence motif of CAL is X-S/T-X-L/V/I. As expected, among the full set of HumLib peptides, 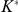 enriches for sequences that conform to this motif. Furthermore, if we allow 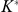 to design peptides varying at the primary motif positions 0 and −2, it achieves an AUC of 0.94 (Text S1 Section S3 and Fig. S2 in Text S1), confirming its ability to identify the motif *de novo*. While 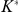 also identified a few non-motif sequences in each case, the HumLib suggests that CAL actually can bind to such sequences, albeit less frequently (10 of 5867 sequences).

Of course, the identification of motif residues, while a necessary test of the algorithm, does not by itself represent a major advance in affinity prediction. The HumLib library shows that only 70 out of 261 sequences with the CAL binding motif bind to CAL. A much more stringent test of the 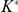 design algorithm is thus to determine how well 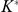 enriches for binders among sequences that match the known CAL binding motif. As a first test, we recalculated the ROC curve considering only peptides in the HumLib library that match the CAL sequence motif, and 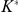 was still able to significantly enrich for CAL peptide binders (AUC = 0.71; Fig. 4B). This search, together with the blind test of 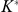 rankings described below, provides a true test that the success of 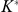 in predicting HumLib binders is not merely due to its identification of peptides conforming to the known sequence motif, but also to its ability to distinguish high- and low-affinity binders among such peptides.

### Prospective Design of CAL Peptide Inhibitors

While SPOT arrays have proven to be a powerful tool for the identification of CAL binding peptides, the highest affinity inhibitors identified to date are composed of at least 10 amino acids. For hexamers, the highest published affinity is for iCAL35 (WQTSII; [60]). Since 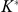 was able to successfully enrich for CAL binders found in the HumLib library, we then used 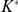 to prospectively find novel, shorter CAL peptide inhibitors, searching over 2166 peptides containing motif-based combinations of the C-terminal four residues. To facilitate accurate experimental binding-constant measurements, each peptide was extended by a shared N-terminal addition of the most frequent 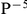 and 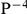 residues among HumLib binders(WQ), yielding hexamer sequences that exhibit a higher baseline affinity [59]. Both top- and bottom-ranked sequences were chosen for experimental validation. The 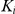 value for each peptide hexamer was determined using fluorescence polarization [59] (Table 1). We used the same FP protocol to confirm the affinity of the acetylated iCAL35 reference peptide for CAL (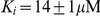).

**Figure 5:**
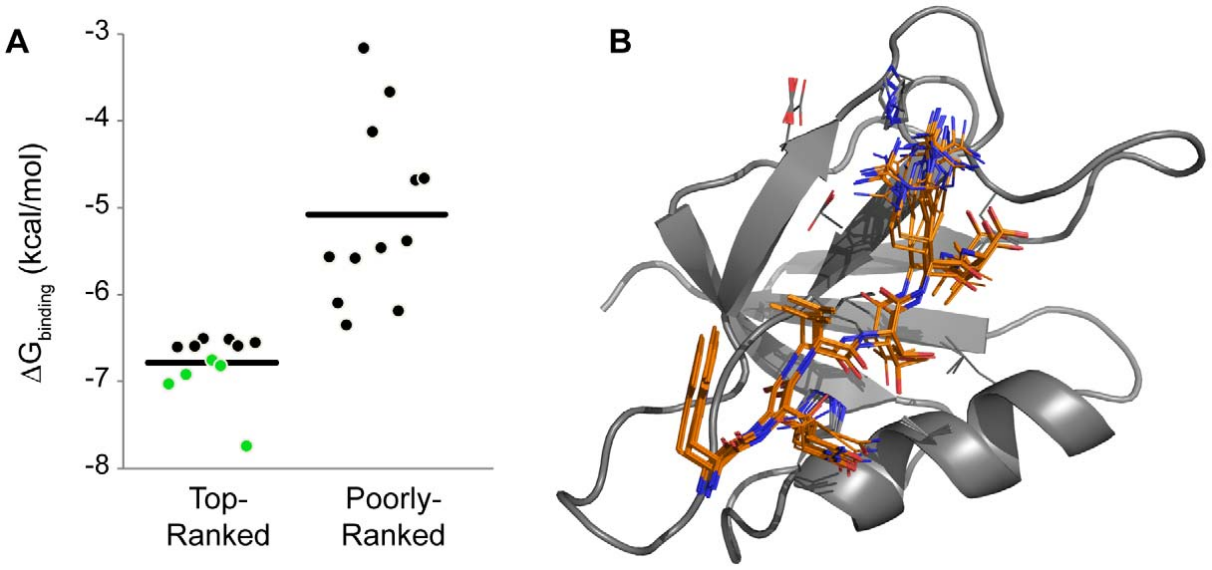
(A) 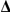
G values for top- and poorly-ranked 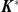 predictions that were experimentally tested using fluorescence polarization. Predictions plotted in green denote that the binding affinity was higher than the best previously known hexamer (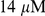). Horizontal line represents average 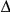
G for plotted sequences. Sequence information and binding data can be found in Tables 1 and 2. (B) Ensemble of top 100 conformations for the peptide (kCAL01: WQVTRV, orange sticks) with tightest binding to CAL (gray ribbon).

All of our top-ranked inhibitors are novel CAL ligands, for which neither predicted nor experimental affinities were previously available. Remarkably, all of the top predicted peptides bind CAL with high affinity (Fig. 5A, Table 1). The tightest binding predicted peptide (kCAL01, WQVTRV) had a 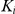 of 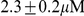. While this affinity is comparable to that of several other PDZ inhibitors [66], [67], solution-state measurements show that the CAL PDZ domain exhibits systematically weak interactions with target C-termini: note that the 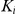 for the wild-type CFTR sequence (TEEEVQDTRL) is 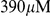 and the best known affinity natural ligand (ANGLMQTSKL) for CAL is 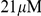 [60]. Thus, our design algorithm successfully identifies high affinity peptide inhibitors of the CAL PDZ domain, with 170-fold higher affinity than the interaction we were trying to inhibit and 9-fold higher affinity than any comparable natural ligand. This peptide affinity advantage may be important in physiological applications, since the native CAL∶CFTR target interaction may involve additional sources of affinity outside the PDZ binding pocket [4], [59], not available to a peptide inhibitor.

We also performed further analysis of the HumLib SPOT array used for 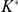 validation. Selecting the most common amino acid at positions 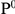 to 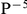 among HumLib binders yields the sequence WQSTRL (HumLib01, Fig. 3C), which is ranked in the top 50 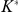 predictions (out of 2166). This sequence is also the strongest binder identified among the ProLib sequences (see below, and Fig. S3 in Text S1). However, when we measured the CAL binding for HumLib01 using fluorescence polarization (FP) it exhibited a 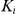 value of 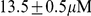, only a marginal improvement in affinity compared to iCAL35 (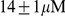). In comparison, five of the eleven top 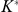 predicted sequences we measured with FP show an improvement in binding compared to both iCAL35 and HumLib01, and kCAL01 shows a six-fold improvement over both iCAL35 and the HumLib01 sequence.

The best inhibitor found through previous FP and array screens involves a fluorescein group modification to a peptide decamer (F^*^-iCAL36, F^*^-ANSRWPTSII, 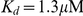). kCAL01 rivals this binding affinity despite the computational search library restriction to only allow amino acids and hexamer sequences. Critically, at 830 Da, kCAL01 has approximately twice the binding efficiency (ratio of inhibitor potency, 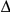
G, to molecular mass) of F^*^-iCAL36 and is much closer in size to typical drugs. This makes kCAL01 a very promising inhibitor compared to F^*^-iCAL36 and other discovered inhibitors.

Furthermore, as suggested by our retrospective tests, the tight binding of our top-ranked sequences was not merely a consequence of the underlying CAL-binding motif used to select candidate sequences for evaluation. To establish this, we selected a set of poorly-ranked peptides to minimize sequence similarity and evaluated their CAL-binding affinity experimentally. Almost all of the poorly-ranked sequences bound CAL, consistent with their motifs (Fig. 5A). Reflecting the enrichment of CAL binders in the pool, the two poorly-ranked peptides with the best affinities (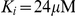 and 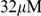, respectively) were indeed close to the affinity of the weakest top-ranked sequence (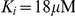). However, all of the poorly ranked peptides bound CAL more weakly than any of the top-ranked sequences (Table 1), and none of them had improved affinity relative to prior biochemical efforts. This suggests that 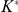 can efficiently distinguish among motif-bearing peptides, allowing it to predict sequences with CAL affinities unprecedented among hexamers.

Detailed analysis of the 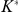 predictions suggests that the use of both ensemble-weighting and minDEE approaches was important in the success of the algorithm. The ensembles generated by 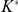 do not have a dominant conformation, i.e., a conformation with significantly lower energy than the others, which would thus dominate in the partition function. For example, in the case of iCAL35 (WQTSII), 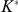 found 75 conformations that were within 0.5 kcal/mol and 454 conformations that were within 1 kcal/mol of the iCAL35 GMEC. In general, the ensemble conformations are consistent with canonical PDZ:peptide interactions and with the conformation of the CAL-bound CFTR peptide determined by NMR [52]. To determine the importance of the ensemble-based 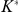 rankings we compared the predictions to two single-structure GMEC-based methods, minDEE [41], and rigid-rotamer DEE (rigidDEE) [68]. Both minDEE and rigidDEE were run with the same energy parameters as the 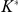 designs. However, since the single-structure designs only compute the energy of the bound state, reference energies [16] were included as in [69] to account for the energy of the unbound state. The inclusion of reference energies for single-structure designs have been deemed necessary by most protein designers to account for the unfolded/unbound state [24], [69], [70]. 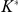 does not need reference energies since it calculates a partition function for both the bound and unbound states of the complex [16], [40]. Therefore, reference energies are included to make the comparison between 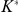 and the single-structure designs more fair. We compared the top 30 sequences from minDEE and rigidDEE and found they had no sequences in common. This supports previous work where we have shown that in over 69 protein design systems minDEE finds low energy sequences that rigidDEE discards by not allowing minimization [41], [50]. In addition, when we compare the top 30 rigidDEE and minDEE results to the top 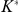 designs we find that they have only three and four sequences in common, respectively. If we had used only GMEC-based approaches instead of 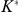, we would not have predicted most of the experimentally successful sequences that 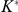 found, including the best inhibitor kCAL01. In addition, the overall sequence rankings show a very poor correlation between the minDEE and 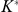 predictions; the same is true of the rigidDEE and 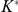 predictions (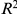 = 0.1 and 0.09 respectively).

### Blind Test of 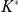 Predictions within the CAL Binding Motif

**Figure 6:**
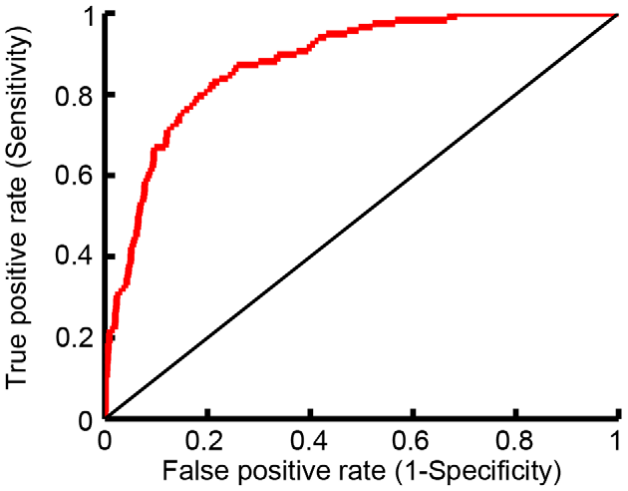
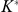
was used to predict binding between the CAL PDZ domain and the peptide array, ProLib (Figure S3), which contained peptide sequences that match the CAL binding motif. The ROC curve shown compares the 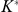 predictions to the observed peptide array binding data. AUC = 0.88.

The prospective peptide predictions demonstrate that 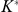 can successfully find CAL peptide inhibitors. Our solution-state binding tests provide robust information for the best and worst K^*^-predicted peptides, but give little information about the CAL binding of the remaining peptides that match the CAL motif. To investigate this experimentally, we designed a peptide library SPOT array (ProLib) based on the HumLib motif combined with substitutional analyses [60]. The resulting sequences closely match our prospective prediction set and the binding of these sequences to CAL was assessed as described in the Materials and Methods section. Using a similar analysis to that performed on the HumLib peptide array we compared the 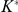 predictions to the CAL binding observed with the ProLib array. We found an AUC = 0.88 (Fig. 6). Note that this AUC is much higher than the 0.71 found when only looking at CAL motif sequences within the HumLib array. One explanation for this improvement is that the experimental setup is closer to the design model used by 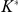. Specifically, the ProLib array uses a mixture of amino acids at 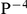 to 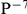 of the peptides, while the HumLib array is composed of decamer peptides. Thus, the ProLib data focuses on the identity of the last 4 C-terminal positions, which better matches the sequence and structure search space of the 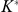 designs. A complete evaluation of the accuracy of 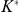 affinity predictions would require the synthesis and FP binding analysis of all 2166 sequences within the CAL binding motif. However, taken together, the FP measurements for the designed peptides plus the ProLib blind test suggest that 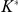 is a powerful filter, efficiently selecting tight binders from a pool of sequences with baseline affinity for the target.

### Biological Activity of the Highest Affinity Designed Peptide Inhibitor

**Figure 7:**
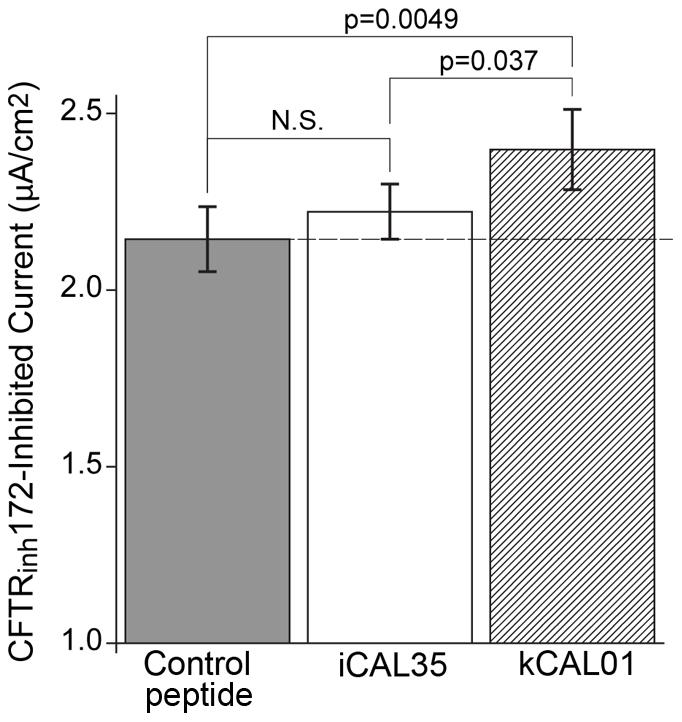
Top binding peptide is biologically active. The ΔF508-CFTR specific chloride flux is shown for a control peptide (kCAL31; WQDSGI; no CAL binding detected), the reference peptide (iCAL35; WQTSII), and the tightest binding design peptide (kCAL01; WQVTRV). kCAL01 shows a 12% increase in chloride efflux over the control peptide. 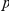 values shown are for pairwise comparisons (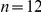). Values shown are mean 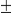 standard error of the mean (SEM). N.S.: *not significant*, 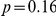.

All of our top-predicted inhibitors successfully bound CAL, which suggests that they should disrupt the degradation pathway of CFTR. The ability of kCAL01 to restore ΔF508-CFTR function was assessed by measuring CFTR-mediated chloride efflux in CF-patient derived bronchial cells expressing ΔF508-CFTR (CFBE-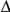
F) using an Ussing chamber apparatus [11]. As a control peptide, we used kCAL31 (WQDSGI), which was ranked as the weakest interactor by 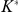 and for which no binding was detected experimentally (Table 2). Fig. 7 shows ΔF508-CFTR chloride secretion across polarized monolayers treated with either kCAL31, the iCAL35 reference peptide, or kCAL01. Previous studies with fluorescently labeled peptides have demonstrated delivery into CFBE-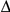
F cells using the BioPORTER reagent [11]. Significance of rescue was evaluated by comparing percentage improvement in chloride efflux to rescue from a well-established “corrector” under identical conditions, and by Student's 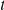-test (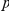-value). Compared to the non-binding control, the previously best hexamer, iCAL35, yields only a slight (non-significant) improvement in chloride secretion (4%, 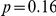). In contrast, chloride secretion following treatment with the designed inhibitor kCAL01 is significantly enhanced with respect to the control peptide (12%, 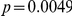) and with respect to the reference (8%, 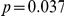) peptide. Indeed, the biological activity of kCAL01 is very similar to that observed under similar conditions following treatment with either the best previously available CAL inhibitor (F^*^-iCAL36) or the first-generation corrector corr-4a [6], [11].

## Discussion

The new 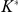 algorithm has enabled the design of the first high-affinity hexapeptide CAL PDZ inhibitor with demonstrated ability to rescue ΔF508-CFTR. By interfering with CAL-mediated degradation, our best designed peptide, kCAL01, can act as a CFTR “stabilizer,” allowing ΔF508-CFTR to recycle back into the membrane. Currently the only well-studied ways to rescue mutant CFTR function with drug-like molecules are through “potentiators” and “correctors” which do not address the problem that ΔF508-CFTR is rapidly endocytosed and degraded at physiological temperatures [9]. Like other CAL inhibitors, kCAL01 should work in conjunction with potentiators and correctors to create an additive effect [11].

kCAL01 was observed to increase ΔF508-CFTR activity by 12%. While this effect is clearly statistically significant (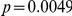), we also wished to assess its magnitude relative to the effect of known rescue compounds. The performance of kCAL01 was benchmarked using polarized human airway epithelial cells derived from a CF patient (stably expressing ΔF508-CFTR; CFBE-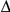
F cells). In these cells, CFTR rescue is more challenging than in heterologous cells, but the levels of rescue observed are more likely to reflect the physiological situation. Since CFTR modulation is extremely sensitive to experimental conditions, and particularly to the type of cells used [8], [71], we chose to compare the performance of kCAL01 against the corrector corr-4a. There are two reasons for this choice for comparison: (a) corr-4a is a well established benchmark for CFTR correctors [72]; and (b) directly comparable data are available based on our previous studies [1]. Under identical experimental conditions, corr-4a produces a 15% increase in ΔF508-CFTR levels in CFBE-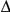
F cells [1]. Thus, the 12% increase seen with the kCAL01 inhibitor peptide is similar to that produced by a first-generation corrector. Since corr-4a and kCAL01 have orthogonal mechanisms of action, this enables additive rescue as an attractive treatment option. Specifically, in the long term the therapeutic impact of CAL inhibitors is likely to be enhanced by their ability to provide additive rescue with correctors, offering the prospect of combination treatment [11].

To design kCAL01 we developed a novel, provable, ensemble-based protein design algorithm for protein-peptide and protein-protein interactions. The validation of 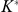 by comparing its predicted binding scores to CAL peptide-array data demonstrates 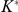
's strong ability to enrich for human protein sequences that bind CAL. While the HumLib array showed that CAL binds a specific motif, it also shows (along with the ProLib array) that CAL does not bind all sequences that match the motif. In HumLib, 191 of 261 sequences that match the motif did not bind CAL. Moreover, all of the peptides synthesized for this work (kCAL01-kCAL31) match the CAL motif, but have a wide range of binding affinities. Therefore, 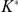 needs to perform the difficult task of differentiating the affinities of peptides that share the CAL motif, rather than merely separating motif from non-motif sequences. The HumLib analysis, FP analysis of top and poorly-ranked 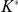 predictions, and the ProLib analysis all show that 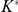 is able to enrich for sequences within the CAL PDZ sequence motif that have high-affinity interactions with CAL.

The experimental validation of top-ranked 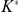 sequences confirms that 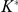 prospectively predicted novel high-affinity CAL peptide inhibitors. Compared to the inhibitory constant of the natural CFTR C-terminus, the designed sequences are much stronger binders. Indeed, our approach found peptide sequences that bound more tightly than iCAL35, the best previously known hexamer sequence. Interestingly, even though iCAL35 binds to the CAL PDZ domain, it is unable to mediate significant or substantial rescue of ΔF508-CFTR in CFBE-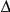
F cells (Fig. 7). The designed inhibitor's improvement in binding directly translates to increased ΔF508-CFTR activity in CF-patient derived airway epithelial cells, demonstrating the value of using our computational approach to design protein∶peptide interactions.

Current therapeutics known to rescue CFTR function are small molecules generally discovered through high throughput library screens [72]. To find CFTR stabilizers we needed to discover inhibitors that could block the CAL-CFTR PPI. Unfortunately, small molecules that inhibit PPIs are rare and the development of such inhibitors has been very difficult due to the shallow, distributed nature of the interfaces [73]. Therefore, we have focused on tools to design peptide inhibitors, developing and validating a new 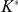 algorithm that has identified low molecular weight, high-affinity sequences. While our previous work employed high-throughput peptide arrays to screen for inhibitors [60], the computational design approach can easily and accurately be expanded beyond the limits of peptide array synthesis, providing a novel avenue for identifying CF therapeutic leads with improved affinity, specificity, and proteolytic stability.

In this paper we have focused on improving peptide inhibitor affinities, but our success suggests that 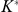 can also be used to improve peptide specificity and proteolytic stability. For optimal biological efficacy, CAL inhibitors should avoid off-target effects, including interactions with other CFTR trafficking proteins (Fig 1B), such as the NHERF family [3]. To achieve peptide specificity, 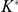 could be run to find peptides that did not bind well to these off-target interactors, a process known as *negative design* [16], [42]. The experimentally-tested poorly-ranked 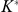 predictions all had a worse affinity for CAL than the top-predicted peptides (Tables 1 and 2). This suggests that 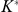 has the capability to conduct negative design for the CAL system. Also, we have shown the successful application of 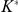 negative design to other biological systems [42]. Finally, since the efficacy of natural peptides is often limited by proteolytic stability, it could be beneficial to extend the 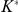 software to incorporate non-natural amino acids, such as d-amino acids, into the design search space. This will allow the design of compounds that inhibit CAL, but cannot be degraded as readily as linear L-peptides.

The 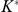 scoring function uses energy terms for electrostatics, van der Waals energy, and implicit solvation. 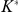 also utilizes an approximation of conformational entropy factors through its ensemble-based scoring [16], [41]. Analysis of these components can potentially identify important interactions in the top peptide inhibitor designs. Comparing the average energy contribution for the top 30 predictions to the median for all designs we find that all components contribute favorably to the peptide binding, with van der Waals giving the largest benefit (−11.2 kcal/mol), followed by electrostatics (−10.9 kcal/mol), and finally solvation (−8.2 kcal/mol). However, even within the top 30 predictions the dominant energetic component varies greatly (electrostatics is dominant for 12 sequences, van der Waals for 6 sequences, and solvation for 12 sequences).

Tidor and co-workers [69] have suggested that design predictions are best when re-ranking structures using a purely electrostatic energy function. We addressed this possibility by comparing the AUC obtained from a purely electrostatic function vs. that obtained from our complete energy function. If we use only the electrostatic term, the AUC was 0.61 (bound energy only) or 0.66 (bound minus unbound). Both values are significantly lower than the 0.84 AUC value obtained with the full function. Thus, while electrostatic terms are important to the success of the algorithm, inclusion of a more complete energetic model improves the prediction. In fact, no individual energy term outperforms the 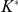 score when classifying the peptide array data. Thus, 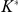 predicts its successful designs by accurately incorporating all three energy terms through ensemble-based scoring.

Many of the binding sequences identified by 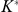 contain a positively charged residue (R/K) at 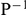. Similarly, in the HumLib array, about 26% of the sequences that we consider to be binders contain a positively charged residue at 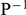, and in the ProLib array 53% of the binders contain an R/K at 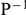. Based on our previous NMR analysis [52], the 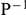 Arg can form a salt-bridge with Glu309 on the periphery of the CAL binding site (Fig. 1A), an electrostatic contribution that could theoretically dominate the ROC curve analysis. However, because 74% of the top binding sequences in the HumLib array do not contain the 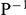 R/K, the strong 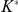 AUC values suggest that it must also correctly predict these sequences. To test this assertion more forcefully, we removed all of the sequences with a positively charged residue at position −1 and then recalculated the ROC curve. This results in an AUC of 0.82, almost identical to the value of 0.84 obtained with all sequences. Thus, consistent with the significant contributions of each term in the energy function, the ROC behavior of the algorithm is not dependent on the presence or absence of a positively charged residue at 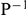.

A small number of 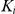 values were used to train the new 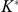 algorithm to properly scale energy terms for protein-peptide interactions, which can now be used for additional protein-peptide interaction designs. Besides the training, the only system specific data used was the input starting structure and CAL sequence motif. The sequence motif was used as an optional filter to expedite the search, but should not affect the ability of 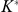 to find high-affinity inhibitors. As seen from the HumLib peptide array comparison, 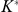 yields a higher ROC AUC when considering the entire array, which implies that 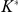 is better at distinguishing CAL peptide inhibitors from the entire sequence space than from within only the known sequence motif. This suggests 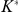 will be able to find new high-affinity inhibitors if the search space is expanded.

Beyond its utility in the design of enhanced CAL inhibitors, the 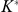 algorithm represents a general framework for analyzing PDZ domains and other protein-protein interfaces. PDZ domains are among the most common interaction domains in the human genome [74]. Using traditional biochemical approaches, the characterization of the binding affinity of candidate partners, as well as the identification of high-affinity reporters and inhibitors, often requires the individual synthesis of dozens of peptides, many of which fail to interact robustly. As shown for CAL, 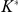 offers a facile mechanism to predict affinities and to design novel ligand sequences using only an initial input structure. Furthermore, the proofs and algorithm presented here provide a general approach for modeling peptide-mediated PPIs that regulate a wide variety of critical physiological processes.

### Availability

The source code of our program is freely available, and is distributed open-source under the GNU Lesser General Public License (Gnu, 2002). The source code can be freely downloaded at http://www.cs.duke.edu/donaldlab/osprey.php.

## Supporting Information

Table S1 Binding data from CAL HumLib peptide array.
(PDF)

Text S1 Proof of Lemma 1 and 2. Additional methods detailing training of energy function weights and computational design of CAL motif residue positions.
(PDF)

Text S2Structural coordinates for the 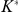 design starting template of the CAL PDZ domain:CFTR C-terminus complex.(TXT)
