# Supplementary material for: Computational Design of a PDZ Domain Peptide Inhibitor that Rescues CFTR Activity
Source: PLoS Comput Biol. 2012 Apr 19;8(4):e1002477. doi: 10.1371/journal.pcbi.1002477 (PMC3330111; doi:10.1371/journal.pcbi.1002477)
Supplement: Table S1 — Binding data from CAL HumLib peptide array. (PDF) [file pcbi.1002477.s001.pdf]

Table S1: Binding data from CAL peptide array.

ID	Sequence	BLU	ID	Sequence	BLU	ID	Sequence	BLU
O60591	NALEEPKGTRL	289032	P35913	PAPKSSTCCIL	59847	P25024	TSSSVNVSSNL	34050
P50150	FREKKFFCTIL	244862	Q9Y698	SNTANRRTTPV	59748	P27037	NVDFPPKESSL	33908
P23508	NSRPHTNETSL	233260	Q9NP72	GGACGGYCSVL	59689	P05181	PRYKLCVIPRS	33756
Q9Y289	SSTCILQETSL	212567	P02404	RRHWRRTKLGL	59368	O43196	QEVLPAATSIL	33683
Q9UL62	DGQEEQVTTRL	201912	O15439	PSTLTIFETAL	58805	Q9UKG9	DMIQLMNSTHL	33674
P52790	VACRLAQLTRV	192017	Q15760	PINSNPPNTFV	58371	P01705	FGGGTRVTVLS	33549
O60760	NWIKRRPQTKL	187987	P35354	TVLLKERSTEL	58317	Q15067	YKHLKSLQSKL	33517
P22749	LRLCIPSTGPL	163083	O43365	RIQEAPKLTHL	57789	P53778	LGARVSKETPL	33385
P09017	EQQRAEDITRL	154368	P98066	KNFLAGRFSHL	56857	P30443	GSDVSLTACKV	33337
P34998	SFHSIKQSTAV	152494	P21941	KRLAILENTVV	56571	P36382	SSKARSDDLSV	33248
P25025	GSSSGHTSTTL	151871	P20020	GSPLHSLETSL	55717	Q13613	SHSATSVHTSV	33234
Q03052	HHHHTLPGSVQ	147564	O95477	LQDEKVKESYV	55519	P17252	QFVHPILQSAV	33168
P00973	QAEEDWTCTIL	144120	P15153	TRQQKRACSLL	55320	Q13255	LRDYKQSSSTL	33132
Q14135	SYRRPPSATCV	139137	Q9Y2T5	KPRKRANSCSI	53958	P56748	SPSVYSRSQYV	33010
Q14916	DWAKEKQHTRL	129725	Q14155	MNDPAWDETNL	53933	P47211	DTPPSTNCTHV	32555
P30670	PFRPQKVCSFL	128534	O60896	VWRSKRTDTLL	53200	P00352	TVTVKISQKNS	32552
Q01668	LADEMICITTL	122709	Q9UBN1	VSMLNRRTTPV	53010	Q12951	GVLYPREGTEV	31988
Q99527	EQSDVRFSSAV	122169	P07951	LDNALNDITSL	52634	P47890	KLIWVRKIHSP	31948
O15049	PWSPRLESSKI	122101	P23219	GPAVERPSTEL	52229	P46089	IPFRSRSPSDV	31937
Q9UJW7	CGKGFSYSSVL	117998	P35914	SSKVAQATCKL	50529	Q92667	AQWVDSYYTSL	31882
P12034	SVKYRLKFRFG	117990	P09104	AGHNFRNPSVL	50202	P21757	HSEDAGVTCTL	31842
O15287	FRTSLPKSCDL	117487	O75792	LERGLESATSL	50077	O43281	QFTTLLTSLAP	31721
P56381	SNVKIVKVKKE	116107	Q9Y2T3	GKQVVPFSSSV	49757	P09958	RTAFIKDQSAL	31438
O60359	NNPANRRTTPV	107942	P47872	QSQGTCRTSII	49280	Q9UHE8	KINKTEICSQL	31269
O14862	TIKVIKAKKKT	107439	P28335	SSVVSERISSV	49257	O94956	GPGKKPEDSRV	31225
Q15418	RRVRKLPSTTL	107169	O00764	DPEIVVQATVL	49083	P01733	TFGSGTRLTVV	31107
Q13936	LQDSRVYVSSL	106809	P30872	NGTCTSRITTL	48357	P53992	VREIGTVTYLM	30958
Q92834	TERRSKSCTIL	104909	P11766	SGKSIRTVVKI	48003	P12756	KYRLIYLYCSF	30917
P13569	ETEEEVQDTRL	103406	P18462	GSDMSLTACKV	46986	P06887	FGTGTKVTVLR	30832
P14415	RVAFKLRINKT	102531	P46721	VLKDDELKTKL	46419	P01716	FGGGTRLTVLS	30829
Q13011	KELKTVTFSKL	101678	P35499	TVRPGVKESLV	45998	Q9ULV3	PLPRRSTRLKT	30410
P48448	WGMGSQSCTLL	101596	Q93070	TSVIIFSKSRV	44955	P35715	YRPRRKTKTLL	30356
Q99712	LRTLLLQQSNV	100826	P45844	FVLRYKIRAER	44835	Q15392	STTWCRTCWCP	30345
O14578	QVNKVWDQSSV	99732	P41240	LEHIKTHELHL	44003	P04435	HFGDGTRLSIL	30134
P05997	FGVEIGPVCFV	98936	P33993	VNASRTRITFV	43608	Q03112	ESSAIQSISHV	30088
Q06495	ALPAHHNATRL	98912	P01704	FGGGTYVTVLR	43373	Q9Y6H6	HVYIKNRVSMI	29974
Q16581	NVISERNSTTV	98814	P08588	CRPGFASESKV	43358	Q01524	VMGINHRFCCL	29895
P16473	QISEEYMQTVL	98102	O75616	VDIRLSVKLLK	43271	Q16254	CDLFDVPVLNL	29844
O00476	EWAKERKLTRL	97639	Q14166	QPGGCHVTCLV	43038	P30305	RRELCSRLQDQ	29760
P56539	SSIKVVLRKEV	97400	O14905	RLVAFSCHCQV	43010	P17655	DLISWLCFSVL	29748
O95377	RPRDHVKKTIL	94806	P25445	SNFRNEIQSLV	42930	O43555	PRPAPPSSNKV	29696
Q10588	LFLVLASRTQL	92449	P28222	FHKLIRFKCTS	42792	Q15165	TLYHRALYCEL	29687
O60299	SRLERIESTEI	88885	P11169	IEPAKETTTNV	42750	P54852	GIIYIHLRKRE	29508
P51843	DMMLEMLCTKI	87919	O43929	RQWATSSLSWL	42420	P50539	SSASVKLSFTS	29499
P53618	KINLSQKETSI	86001	P32241	SSSFQAEVSLV	42325	P15313	RLQDLAPDTAL	29484
P13056	IHFTRRAITDL	84892	Q9UG63	EPQLTKRTHNV	41737	O60229	LGPGDPFSTYV	29440
Q08426	QSLAGSPSSKL	84380	Q15303	PPPYRHRNTVV	41456	P55060	GYLQAASVTLL	29439
P35222	SNQLAWFDTDL	83064	O75015	TGLYFSVKTNI	41339	P48065	GLIAGEKETHL	29392
P09543	KGGALQSCTII	82568	Q00056	PSTSTPVPSSI	41120	P20769	YSTTVTLFKVK	28978
O14972	TENFPLKLCRI	82301	P47775	LAQRARSPSDV	40656	P10767	IMTVTHFLPRI	28841
Q12979	KRNTLYFSTDV	80217	Q15139	MKALGERVSIL	40514	O75964	IGKRGIIGYDV	28752
Q00722	IAKADAQESRL	80178	P41595	GDKTEEQVSYV	40388	Q05066	QRDRYSHWTKL	28664
P27816	TLDSQIQETSI	80125	P20809	VRGLLLLKTRL	40249	P26599	HLRVSFSKSTI	28643
O14832	ARLVKGERTNL	79085	P30044	CSLAPNIISQL	40000	O95336	LTVPFEKHSTL	28566
Q15349	RGMKRLTSTRL	78837	Q9ULD4	GPHSFVTSSYL	38658	Q99765	QSFCTVLVSSI	28565
O95049	DGYDWGPATDL	77093	P23378	SPFSEQKRASS	38408	O95822	LVAQFQKNSKL	28513
P56730	FVPWIKSVTKL	77071	P15529	ETHREVKFTSL	38289	P55082	YKDGAYENCQL	28494
Q15126	ENLIEFIRSRL	77065	P14679	EDYHSLYQSHL	38112	Q01453	GVIYVILRKRE	28436
P02654	QKVKEKLKIDS	76930	P01135	RTACCHSETVV	38003	O43924	VSTSRVRLFYV	28284
O95998	GGKGGLCQSSL	76290	Q13324	SFHSIKQTAAV	37825	Q13574	MIQREDQETAV	28264
P51812	RGIKKITSTAL	75629	P10267	SKRDQIVTVSV	37815	Q15147	AEMDRRPATVV	28149
P35346	AANGLMQTSKL	75304	P11234	KKSFKERCCLL	37190	Q15040	VEAHQSWRTDV	28111
P98153	RHSRSSLNTVV	74938	Q13268	NIAVAGYSTRL	37168	Q13336	QAKKRMVESPL	28087
Q01814	GSPIHSLETSL	73965	Q92911	DGGRDQQETNL	36869	O95969	VLVKILKKCSV	28062
Q00597	RELLKELRTQV	73874	O43749	KKVVGRVVFSV	36643	P09874	KLKFNFKTSLW	27981
Q14940	WCIQFNRGSRL	73802	P08311	FKLLDQMETPL	36515	P30046	IGKIGTVMTFL	27956
P05981	HSEASGMVTQL	73447	P54709	GRVMFKITARA	36419	P09430	DDANRNYRSHL	27855
P11274	KRQSILFSTEV	72848	Q14524	PSPDRDRESIV	36278	Q15166	TVFGKTLYCEL	27820
Q14165	FIPTLFCLCRL	72701	P54851	GMMYLILRKRK	36213	O43613	VVLTSVTTVLP	27793
P02452	FGFDVGPVCFL	71152	P19320	YSLVEAQKSKV	36164	P46095	VPFRSRSPSEV	27759
P31249	RLPEAPKLTHL	70828	P01702	FGGGTRVTVLG	36142	Q14195	PGGRSNITSLS	27722
O75593	GPGWLLSWCSL	70639	P41594	IRDYTQSSSSL	36079	O60756	MLLLLLLNYNM	27687
Q12768	NFIFDEFRTVL	70518	P56750	TMLSKTSTSYV	35901	Q13201	TTFSGYLLYRT	27684
O14904	RVVTRPCQCQV	69941	Q9UKP5	SSCNLAKETLL	35557	P49418	LFPENFTRRLD	27582
O00633	DEDQHTQITKV	68498	P09016	AKDHHTDLTTL	35512	O14924	PKTSAHHATFV	27510
P02461	FGVDVGPVCFL	68469	O14775	GSWDHTLRVWA	35356	P22303	HYSKQDRCSDL	27442
P33402	IGTMFLRETSL	68116	P43353	MEAQGCSCTLL	35208	P11168	EMKFLGATETV	27434
P41587	QSFLQTETSVI	67977	Q9Y5I7	TAKMYAVDTRV	35001	P09693	SHLQGNQLRRN	27404
P07942	SQKVAVYSTCL	66823	Q01970	DSESQEENTQL	34814	O14493	AARSAAASNYV	27350
P23510	IHQNPGEFCVL	66775	P19961	PFIAIHAESKL	34618	Q01118	AKEKSPIQSQI	27258
P11233	AKRIRERCCIL	62913	P23560	CVCTLTIKRGR	34434	P16106	QLARRIRGERA	27254
O43451	NFTSLTWISTL	62533	P32745	EKSSTMRISYL	34401	Q14517	PPLDSQQHTEV	27163
Q15668	WEIPVQIVSHL	62507	P48067	NGSSRLQDSRI	34400	P25800	QLNGTFESQVQ	27142
P43250	SDSEEELPTRL	61813	P01700	FGGGTQLTVLR	34277	P33240	KEQIQKSTGAP	27016
O43711	SSKVPAVTSLV	61382	Q9UBX5	RLRIYVSQYPF	34210	P28906	ARQHVVADTEL	26943
P50995	KILLKICGGND	61296	P18077	RIRVMLYPSRI	34176	Q12959	SYIWVPAKEKL	26911
P25054	RHSGSYLVTSV	60515	Q16348	MIKLETKKTKL	34156	P08949	YRRLLVQILQK	26848
P02458	FGVDIGPVCFL	60447	Q06889	VSLAPVVTTCA	34051	P56937	DNQARLSGSCL	26839

ID: ID from Uniprot Database (http://www.uniprot.org/) **Sequence**: Listed sequences match the Uniprot ID, but for the array experiments and for the computational predictions, the cysteines were changed to serine. **NA**: not available. **BLU**: biochemical light unit.

Table S1: Binding data from CAL peptide array.

ID	Sequence	BLU	ID	Sequence	BLU	ID	Sequence	BLU
O15228	TPIGKPATAKL	26812	P28698	LIQHQRVHSAE	24477	Q13009	FAPSRKLNTEI	23706
Q13490	IIKGTVRTFLS	26743	O15427	GEVVHTPETSV	24463	P16234	DSSDLVEDSFL	23695
Q15743	GSGGFPTGRLA	26646	O60469	NNPYAKSYTLV	24449	Q03060	TLKDICSPKTD	23689
P01730	PHRFQKTCSPI	26595	P03923	VYIVIEIARGN	24446	P98073	RFTEWIQSFLH	23689
P46937	KLDKESFLTWL	26594	P01703	FGGGTKLTVLR	24424	P53701	RMKVAWWRWTS	23686
P05163	DGDFKIKVRGL	26588	O94823	SHRRSQSSLTI	24421	P05121	TVLFMGQVMEP	23673
P07766	DLYSGLNQRRI	26525	P28331	GAQAVEEPSIC	24411	P35228	VQPSSLEMSAL	23669
P07339	NRVGFAEAARL	26330	P11182	ENPAFMLLDLK	24396	O14737	KVMDSDEDDDY	23668
Q15738	VQSFRHLRRVK	26144	O76015	TCGASTTGSRF	24394	Q9NXH9	GPGAAAGPGID	23664
P01773	WGQGTLVTVSL	26023	P08913	ILCRGDRKRIV	24391	P55196	GKRVTNQLSLS	23663
Q16048	LGRVYQSCWQV	26012	Q09019	QPGNSPSGTVV	24386	Q15818	KWTFEACRQIN	23653
P24539	LAKKAQAQPVM	25963	P47881	WRMLTGRRSLA	24378	Q16853	PAFSHGGFSHN	23647
O75604	FYELASPTSPI	25940	O60676	FTVMEKKCEDA	24363	O60248	LAGAPMPLTHL	23647
P35555	NLKMKIQVLLH	25927	P03996	AGPSIVHRKCF	24331	O15431	AVVVDITEHCH	23639
Q11201	SINKIRIFKGR	25906	P45381	LNAKSIRCCLH	24319	P04437	FGTGTRLQVTL	23635
P04208	FGGGTTLTVLS	25848	Q14249	GSLKAITAGSK	24308	Q10589	IVLLGLSALLQ	23621
Q92628	SEEELFSRTHL	25820	P35080	ELALYLRRSDV	24281	P18825	LFRRRRRGFRQ	23618
P51164	GKVEFKLKIEK	25771	P08620	TMKVTHFLPRL	24255	Q92903	EKGILQPTLKV	23615
P36507	NQPGTPTRTAV	25768	Q12983	GRRLTTSTSTF	24255	P30559	SQRSCSQPSTA	23603
P10809	GMGGGMGGGMF	25767	P12694	GEHYPLDHFDK	24251	Q14203	QLHQLHSRLIS	23600
P48047	QKLGRAMREIV	25731	O00341	TIQISELETNV	24236	Q14790	TLRKKLVFPSD	23588
P57735	LGEKRACCISL	25729	O95371	RRLLGKGREVG	24236	P51398	PSLLERHCAYL	23586
Q05682	SVDKVTSPTKV	25704	P55040	KSKSCHDLSVL	24226	P05177	EHVQARRFSIN	23584
P52948	APPPQVEKKGQ	25695	O00322	MLIAMYFYTML	24223	P36222	TNAIKDALAAT	23583
O75912	VIGHEDLETAV	25679	P24347	FGCAEPANTFL	24220	Q15878	LLSDTEEDDKC	23571
P05091	TVTVKVPQKNS	25673	P49821	AQQHQARQAAS	24199	P24298	RFHAKFTLEYS	23569
O43309	PEWQSCFRRDL	25658	P37058	VAYLKLNTKVR	24192	P12110	EPGPPGDPGLT	23564
P26885	ELLKIERRTEL	25624	P13349	ASSSRLIYHVL	24180	P15586	VRTRRFSKHLL	23562
O75690	CCVPICCQCKI	25596	Q16531	IKVVEELTRIH	24178	Q12950	LRRRKRFQRHQ	23554
Q14330	RSLSNINSEML	25578	P01816	WGQGILVTVSS	24172	P56746	SFGKYGRNAYV	23551
Q9UJT1	LEQVVASYCNL	25576	Q99583	PSFPVSTLKLA	24121	P17317	LIGKKGQQKTV	23540
P01709	FGTGTKVTVLG	25487	P40261	SLVARKLSRPL	24120	O60551	TDSEKVGLVLQ	23536
P48436	EQPVYTQLTRP	25455	P41222	LPQTDKCMTEQ	24110	Q9UH64	WARVGWIQLPG	23531
P78508	GSALSVRISNV	25417	P13667	ATKLSRTKEEL	24108	P21953	CYDALRKMINY	23524
P00846	LLVSLYLHDNT	25370	P13725	KRLMTRGQLPR	24085	P23142	NVRIFVSEYWF	23494
P25100	ADYSNLRETDI	25365	P05166	RPWRKHANIPL	24082	P47900	PEFKQNGDTSL	23479
Q02363	LMSNDSKALCG	25335	P47893	WQIFLGRRSLT	24071	P34810	IRRRPSAYQAL	23474
Q99489	ALRTPIPKSNL	25334	O00624	LPASEDIKLQT	24065	P19338	HKPQGKKTKFE	23446
P42857	EQETEAAEKSA	25311	P08579	SHAMKITYAKK	24065	P29122	QFCCRTCLLAG	23441
P30837	TVTIKVPQKNS	25305	O75947	PYWPHQPIENL	24058	P08908	KKIIKCKFCRQ	23432
Q09327	PARGKLDEAEV	25281	P25942	KESRISVQERQ	24032	P32456	SKSLEPICNIL	23431
P22830	ETKSFFTSQQL	25268	Q9NQ66	DIPGKEFDTPL	24018	Q16621	VPRGTKMEATD	23419
P22692	DCHQLADSFRE	25262	P43007	APELESKESVL	24002	Q15077	KLTAKWQRQGR	23419
Q14957	WRRISSLESEV	25249	O95236	IYQRLNPCHTH	24001	P12036	KATEDKAAKGK	23416
P01699	FGGGTKVTVLG	25248	P14920	KKLSRMPPSHL	23998	Q9UNI6	LPVLGSQTGKI	23414
P23634	DSSLQSLETSV	25202	Q15072	HHIRHQKIHTH	23974	P08174	LGTLVTMGLLT	23413
Q13607	KFSGLTSKLAT	25167	Q04446	ALILQNVDLPN	23965	O75899	PPSFRVMVSGL	23413
P28838	EFLLRFSQDNA	25151	O75521	VVNFLSRKSKL	23957	Q14934	FPAPPGEEPPA	23413
P20813	PPTYQIRFLPR	25117	Q01101	ILLQVPVRPAC	23947	Q05215	FYSLGLSFASL	23398
O60503	ELTKLNVSKSV	25116	Q15647	SLNQAVVSKLA	23936	P09619	APRAEAEDSFL	23397
O95990	IATLTSEEREL	25102	P11166	LFHPLGADSQV	23934	P51582	SSCSTPRADRL	23395
Q9UNA0	SAFKQCLLKKC	25083	P48426	RFLDFIGHILT	23925	P00558	LPGVDALSNIL	23382
P28223	SDGVNEKVSCV	25020	Q9UHG3	DGLYEKLKTEL	23924	Q14592	NGFIVEETLPL	23381
Q14416	REVVDSTTSSL	24980	P08651	GIYQAQSWYLG	23920	O00321	CAGGGRGAETQ	23368
P49913	FLRNLVPRTES	24960	P49646	ASSYSSSYLGD	23920	O95865	LCLVLSTRPHS	23361
Q05048	ARFWYRRSTTD	24937	Q9UHT4	QFFILITITKY	23911	P01028	LQEYGTQGCQV	23359
Q15062	ERDSRESWRAA	24933	P49146	PNDSFTEATNV	23908	Q9Y2Q3	GPIPPAVNARL	23346
Q14832	REVLDSTTSSL	24930	P24071	LTFARTPSVCK	23902	O94777	LKTKRVTKKAQ	23345
P26436	CCRNQSFCNKI	24913	P56182	QEPEKKKKRRE	23897	O60669	QSVTSERETNI	23323
P98172	PQSPANIYYKV	24897	P06748	IQDLWQWRKSL	23888	Q9UI10	PVVLRVKSSDQ	23321
Q16661	LCVNVACTGCL	24877	Q15124	RTGRRGPTVIT	23886	P30405	KIVITDCGQLS	23319
P11844	VGSLRRVTDLY	24869	Q16517	VLGERRQRAPN	23885	P08912	KLYWQGNSKLP	23318
P37198	EQERSFRITFD	24831	P98095	KMHIFFTTFAL	23876	Q15722	ASSPLKLNELN	23311
P01567	TNLKKGLRRKD	24816	Q08431	RIALRLELLGC	23875	Q13489	TIKGTVRTFLS	23307
P41208	FLRIMKKTSLY	24809	P03999	STVSSTQVGPN	23875	P27707	VEKVKEFLSTL	23306
Q07815	TRCLRMRPPRS	24793	P41146	CKTSETVPRPA	23872	P06576	KADKLAEEHSS	23301
P43003	TEKPIDSETKM	24775	P06318	VFGGTRLTVLG	23871	P42696	RFRSLVCFIPP	23294
Q10713	GRLPRTYRLFR	24761	Q12982	NGKQDEPKNEQ	23869	P00740	VNWIKEKTKLT	23291
P22914	PAVQSFRRIVE	24760	P08100	SKTETSQVAPA	23861	P13798	AVLWLRTHLGS	23289
Q02086	HYKTHLVTKNL	24760	Q92466	IWSQEEARTRK	23859	Q01518	KLVTTVTEIAG	23283
Q14961	LEMTRKRVLHA	24755	O75438	ELQPSEEVTWK	23859	P28328	SGIEMSEVNAL	23279
P53634	IAVAATPIPKL	24741	P10515	KYLEKPITMLL	23856	P48444	TTFLVDKYEIL	23273
P12838	VSFTYCCTRVD	24720	P06729	AAENSLSPSSN	23839	O43169	YRYYTSESKSS	23262
P32926	LCTEDPCSRLI	24644	Q05586	QLQLCSRHRES	23837	Q15121	IIKLGPPPKKA	23262
P15169	MEMRQLQRGPA	24636	O15519	TLRKKLILSYT	23824	P42356	MIQYYQNDIPY	23255
P51178	ATLFVKISLQD	24622	O00408	PINGCCSLDAE	23821	P53677	MTKAGKFQVRT	23254
O15511	IVRVLTARKTV	24613	P20800	PRSTHSRWRKR	23818	Q9Y263	SECCRFILNLL	23252
Q92826	VLAKVKNSATP	24575	O43709	LVWPLMAHLMT	23815	P13497	FRVQKRNRTPQ	23251
Q14181	SPCIAVQVVRI	24564	O43933	MFRPGQKVTLA	23811	Q14656	PRFHPSGGKTR	23248
P11836	ESSPIENDSSP	24563	P27169	TVFHKALYCEL	23808	P13284	STSSLRSVCFK	23247
P26371	CCVPVCYQCKI	24557	P50151	PFREPRSCALL	23806	O60238	KRLSTPSASTY	23244
Q9UK32	RSMKKRTSTGL	24545	P10316	EGLPKPLTLRW	23799	P49641	MEIATFRLRLG	23239
Q99679	RSKGPLNGCHI	24544	P46459	REEGASPLDFD	23781	P04001	EVSSVSSVSPA	23237
Q12882	RGVPLSVNPVC	24532	P30939	KAFQKLVRCRC	23777	P22059	KQDWSSCPDIF	23236
P35372	ENLEAETAPLP	24525	P04234	SHLGGNWARNK	23763	Q9UK08	FRDKRLFCVLL	23229
P04066	FAWTIKLTGVK	24512	P03951	VDWILEKTQAV	23760	O15120	ATAGSGVQPAQ	23220
Q13614	AQCVTPVQTVV	24492	Q12981	YIVKKRLFPFL	23722	O15145	FMNKSLSGPGQ	23218
O60241	EPPDGDFQTEV	24486	P53420	ISRCQVCVKYS	23710	Q9Y2G3	LTLSTMDSSTC	23215

ID: ID from Uniprot Database (http://www.uniprot.org/) **Sequence**: Listed sequences match the Uniprot ID, but for the array experiments and for the computational predictions, the cysteines were changed to serine. **NA**: not available. **BLU**: biochemical light unit.

Table S1: Binding data from CAL peptide array.

ID	Sequence	BLU	ID	Sequence	BLU	ID	Sequence	BLU
P30988	PLNIIEQESSA	23214	Q9UKL4	GRTQSSDSAYV	22962	P00387	VGHPTERCFVF	22767
Q05315	LTKFNVSYLKR	23210	P47972	VETCEEALLDL	22961	P49069	ELLDYWGSEVP	22766
Q9Y3D2	SVALKFKPRKH	23209	Q14118	PYRSPPPYVPP	22958	P42695	SLRKTPLKTAN	22766
Q13555	SKPIHTTILNP	23208	P52798	LLLILRLLRIL	22956	P98088	LTSKENLPYVL	22761
Q15642	VPTSYLRVTLN	23204	Q9Y210	SMEPNQEETNR	22952	Q16555	PGGRANITSLG	22754
P19174	RRTRVNGDNRL	23193	O60397	IQLSMDQKSDK	22950	P15538	MCPLLTFRAIN	22753
O14834	SPKDYSTKYRY	23191	P01720	FGTGTMVTVLG	22948	P10635	PSPYELCAVPR	22752
Q02962	PAARAAAYDRH	23182	O95967	RLTVFVGAYTF	22938	O00244	TGKTVSYLGLE	22751
Q9Y4L1	GQKRPLKNDEL	23179	P16591	KELTIIKRKLT	22938	P42345	CQCYIGWCPFW	22750
O14683	CLQAEAFNPPS	23178	Q15365	RLSSEKGMGCS	22927	P41271	APHTEEEGAED	22749
O75340	EQYLSMVFSIV	23177	P30953	VIHQKKTFFSL	22926	Q15119	EPKNTSTYRVT	22749
P16415	WLTCLLRHERI	23176	Q99487	TPGAPHHLSSL	22922	P55771	EGSHSVTASAL	22746
P50290	GWRPASLRSWG	23169	Q14061	KECMRALGFKI	22914	Q01718	FKKMIFCSRYW	22744
O75748	TSMNERETGML	23168	P50606	LIGLHFKIKPI	22913	Q13938	FVAMMTSAWQL	22742
Q02846	KLEKARPGQFS	23155	Q13772	EKWLYRTPLQM	22900	P05120	CILFFGRFSSP	22742
Q99943	GDYLKKPGGGG	23145	Q13046	VTVRVSDWTLP	22900	P09668	AACASYPIPLV	22740
Q10586	LSRYQAQHGAL	23138	P01713	FGAGTKLTVLR	22897	Q09472	SNLSQSTLDIH	22739
P22079	LDLSPWASVKN	23126	Q9UN36	GPPGHTMEVSC	22893	O75343	IRRFINSQFVL	22738
Q99677	NGGELMLESTF	23125	O14598	SKGRPSTPLSP	22890	P80095	SFIEQVAVSMT	22738
P07550	GRNCSTNDSLL	23124	Q15438	RKKKVSSTKRH	22890	P31935	GLAYLEETKPL	22738
Q92800	YVGIERETDVL	23124	Q16654	EPKNLAKEVAM	22890	P49189	CVEMGDVESAF	22737
Q13423	LQAKVRESYQK	23124	Q15366	LSSETGGMGSS	22885	O43638	RRRRRFTRQTG	22737
Q14990	GSRFSCRKMIL	23123	P11586	DPETEQVNGLF	22882	P39900	KTLKSNSWFGC	22734
P43119	KAEASVACSLC	23123	Q07444	FIMLTRLVLNS	22877	P51841	KAERQLVRNKP	22733
P38159	RSDRGGGRSRY	23123	Q15027	LSRRSHDLHTL	22877	P40126	HLSSKRYTEEA	22731
P04201	NCNTVTVETVV	23119	P49683	HGQNMTVSVVI	22875	P10145	VEKFLKRAENS	22729
P51160	DDKKSKTCLML	23118	P14923	PPYPTADHMLA	22875	O43422	PTDNSETVENT	22728
P02655	DQVLSVLKGEE	23115	O75474	RIALQPSGSLL	22873	P14406	LAVASFPKKQE	22727
P15259	MEAVAAQGKAK	23115	Q9ULV8	AALGPQDPAPA	22871	O95299	TEVGDKWIWLK	22727
O14863	RTCANCQSSSP	23114	P35268	NQDEEEEEDED	22871	P53355	SYNSISSVVSR	22726
P15428	YDTTPFQAKTQ	23113	Q08050	INWSQFIPELQ	22869	P02248	TLHLVLRLRGG	22724
P39210	NSYLSWKAHRL	23112	Q13093	LQNSSGIEKYN	22869	P07205	ILPGVEALSNM	22722
P20783	VCALSRKIGRT	23112	P18545	ELHELAQYGII	22867	Q9Y466	LLSDMYKSSDI	22720
Q9NRZ5	GNSDSKQKLND	23111	P34949	DLLIFRACCLL	22867	Q12805	RLTIIVGPFSF	22716
P47888	CQLLVGERSLT	23110	Q99797	LDFETFLMDSE	22860	P06316	FGGGTKLTVLG	22716
O60701	LQDPPNKKPKV	23101	P20382	LGRVYRPCWQV	22854	Q07812	TASLTIWKKMG	22715
Q9NWB1	YRGGYNRFAPY	23100	O95936	MGGYYAFYTSP	22852	P12724	PVVPVHLDTTI	22715
Q16720	AAGNPGGESVP	23095	Q16825	LIQFLKSSRLI	22852	O15453	QGFLNPLEFSA	22714
P02570	SGPSIVHRKCF	23093	Q09753	TCYRGKAKCCK	22846	P50542	STLLTMFGLPQ	22713
P05164	ALNLASWREAS	23092	P15954	FLVVRHQLLKT	22843	Q14507	EASATLNSIVA	22712
Q06830	QKSKEYFSKQK	23089	O95452	SGQNAITGFPS	22842	P08559	ANQWIKFKSVS	22711
O00409	GQKEQKETTKN	23081	P23759	KLKSSQMEQFT	22842	P05165	VGEGDLLVELE	22711
Q15120	PRDASKYKAKQ	23080	O00571	YNSQGVDWWGN	22840	O15442	IVIDLPTPRNS	22710
P78358	FLAQPPSGQRR	23066	Q03135	SNVRINLQKEI	22839	P28325	ISILNYKCRKV	22710
P00325	SGKSIRTVLTF	23065	P57088	AFISRLAPTVP	22839	Q08345	RFLAEDALNTV	22709
P31275	KKRLLLREQAL	23065	P13592	LFSAVTLLLLC	22834	P09622	LAASFGKSINF	22709
Q14353	PQMITPLVTKG	23064	P25105	FNQIPGNSLKN	22834	P02689	GVVCTRIYEKV	22704
P07738	EDQGKVKQAKK	23061	Q14690	YVEAKSSVLED	22830	Q9NTQ9	GSAPVDAGGYP	22703
Q9NYP3	VLRDYIYNWRS	23060	P12814	FSTALYGESDL	22828	P05496	LMVAFLILFAM	22702
P30808	PPIPPPPPHQQ	23059	Q00604	ILSCHCEECNS	22826	O43186	KDQSAWKFQIL	22701
P01243	QCRSVEGSCGF	23059	P50391	SLRLSGRSNPI	22826	O15239	YYVSKGLENID	22697
P35663	LPEAPWIHKLL	23057	P78310	IPAQSKDGSIV	22825	P43155	ALLQSHPRAKL	22696
P15812	LVVVDSRLKKQ	23050	Q15048	YGRLAADYFSL	22825	P04118	GICHDAGRSKQ	22696
Q14562	AWRISRAFRRR	23050	Q14093	DEKKDAKKKGK	22822	P29536	LKKVEVPKLLQ	22690
O75306	TQDIVFGEVDR	23050	P07202	GRDTHRLPRAL	22822	O43674	IDHSPKATPDN	22689
P56589	EAFSTPQQLEK	23049	P32119	DDSKEYFSKHN	22818	Q99871	GAAAGSTTSAP	22688
P35212	PSSSASKKQYV	23046	Q9UI46	LNLVREVKIKT	22817	P22966	QFGSEVELRHS	22687
Q15768	PQSPPNIYYKV	23039	Q92791	EAEAEPEPELA	22815	Q13021	YILHAFSIYYH	22687
P57055	SSRGGKCSSSK	23033	P47895	TVTIKLGDKNP	22813	O60312	LLIGASSRRSQ	22685
Q9UHZ2	CQAGYDSEDVI	23033	Q14938	LSASDPGTATF	22811	P30304	KREMYSRLKKL	22683
O60258	TKRTRRPQPLT	23032	O94788	TVTVKIPQKNS	22807	O14967	KSVRKRRVRKD	22682
P49747	EDYETHQLRQA	23029	P54849	IGVLYLVLRKK	22805	P19099	TSPLLTFRAIN	22681
P13611	RWSRRWQESRR	23028	O00567	KKKFHKASQED	22805	P08697	EEDYPQFGSPK	22679
Q9UK55	FLGRVVNPTLL	23023	Q99807	CRVAIYLSERL	22804	Q16656	GQAVEVVTLEQ	22678
O15165	KGKDRKPGNLV	23022	P09529	PNMIVEECGCA	22804	P17643	EKLQNPNQSVV	22678
Q9UI56	HEKPVSRPQNT	23022	P09669	MRKAGIFQSVK	22803	P22413	KTHLPTFSQED	22673
P08237	ITRKRSGEAAV	23016	P78329	GGLWLRVEPLS	22802	Q02413	ITKYSTVQYSK	22672
O14511	PPRAKQDSAPL	23012	O60262	PFKDKKPCIIL	22802	Q15652	SLQWFLVCIRK	22669
Q01523	ISGRLYRLCCR	23010	P27824	RSPRNRKPRRE	22800	P26718	PNTYICMQRTV	22667
P29590	RGLAERASQQS	23008	Q02108	ANFLGKASGID	22798	P23975	RQFQLQHWLAI	22667
P17026	LWLVSVKYRAF	23008	P56715	DLESSREQEDL	22798	Q13608	YKRIQRKFAAC	22667
P41145	LRDIDGMNKPV	23007	P55265	QEEKNFYLCPV	22797	P04424	QVRALLQAQQA	22664
P12107	GFEVGPVCFLG	23003	Q05524	AGRNFRNPRIN	22797	Q02241	FLKYISCILIN	22662
Q13705	NVDLPPKESSI	23000	P31314	TSVTSVASACE	22797	P30874	TLLNGDLQTSI	22662
P11940	AVNSATGVPTV	22996	O43603	AGDSILTVDVA	22796	P48595	TILFYGRLCSP	22661
P01718	FGGGTKLTVLS	22992	Q9Y215	YIDSTPCRYFT	22795	P41219	SELDKSSAHSY	22659
Q13310	QKVGAVAAATS	22988	P10176	LSHLETYRRPE	22794	P49746	PFRRQLLQGRV	22658
Q13258	CSNSTNMESSL	22988	Q15398	ITFSPLQPGEF	22791	Q9Y6J3	KSVLATEHAQT	22656
P08652	TRPLQTVPLWD	22987	Q9UF02	AASPSSAVFSL	22790	Q9Y5N5	ETLSVLKFTKS	22656
Q9NRZ7	SYGNQEFKKKE	22987	Q92597	SAGPKSMEVSC	22790	P11308	SHMPSHLGTYY	22655
P54687	REESDWTIVLS	22985	P40939	HANSPNKKFYQ	22781	Q92685	PKSTQHSKKAH	22652
P33552	FRRPLPKDQQK	22984	O43920	PHHIGKGEPRP	22779	P48736	LGIKQGEKHSA	22652
P02278	TKAVTKYTSSK	22979	O00628	IYDPACLTIPA	22778	P25063	VVSLSLLHLYS	22650
P00414	FLYVSIYWWGS	22975	Q99259	IEEIERLGQDL	22772	P14598	ESTKRKLASAV	22650
Q9P2J9	SESIGAYYKGG	22971	O14718	VSQNPLASGRI	22772	Q14194	PGGRSNITSLG	22644
Q9ULP0	GQVNHTMEVSC	22964	Q16352	IEETTISSQKI	22771	P54762	VQISQSPTAMA	22642
Q04656	GDFREDDDTAL	22962	P34995	RSSRHSGLSHF	22769	P02774	SEIDAELKNIL	22642
P28329	KATRPSQGHQP	22962	P24387	NSIGEFCLSGL	22768	P09919	SYRVLRHLAQP	22641

ID: ID from Uniprot Database (http://www.uniprot.org/) **Sequence**: Listed sequences match the Uniprot ID, but for the array experiments and for the computational predictions, the cysteines were changed to serine. **NA**: not available. **BLU**: biochemical light unit.

Table S1: Binding data from CAL peptide array.

ID	Sequence	BLU	ID	Sequence	BLU	ID	Sequence	BLU
P27216	DFRKLLVALLH	22640	Q14444	GPQDPTEGCRK	22566	P09493	LDHALNDMTSI	22493
Q13619	KDNPNQYHYVA	22640	P01178	ACDAEATFSQR	22565	P16219	IAGHLLRSYRS	22492
Q99576	QVPEAPGGSAV	22639	P29728	RNFWRSSGNRF	22564	P05090	VTDQVNCPKLS	22491
P12891	WFLLLPYISLV	22639	Q9Y2Q5	LEEPLTQVAAS	22564	P13385	VGICLSIQSYY	22491
Q9Y6M9	YIVTRPRERPM	22638	Q13153	AAAKEATKNNH	22564	P50461	GGLTQQVEKKE	22491
Q13393	KEAIVPMEVWT	22638	O95456	TTNEIQSNIYT	22562	Q9Y3P0	DQVAILLFKSG	22491
P36383	KSGDGKNSVWI	22637	P49419	DLPLAQGIKFQ	22561	P11712	PPFYQLCFIPV	22490
P53805	RRPEYTPIHLS	22636	Q01196	ARLEEAVWRPY	22559	P80748	GTKLTVLSQPK	22488
P43246	NEIISRIKVTT	22635	Q92499	GYLPNQLFRTF	22559	Q14094	PCPPLQPVSVM	22487
Q9UGV2	LDRHQTMEVSC	22635	P52758	AIQGPLTTASL	22555	P33991	LTVTGKTVRLL	22485
P50225	AGCSLSFRSEL	22634	P06850	SNRKLMEIIGK	22554	Q9UGL9	NNRSSGCCSGC	22482
P35321	PAPAQQKTKQK	22633	P33260	PPLYQLCFIPV	22553	P14416	FRKAFLKILHC	22481
P46059	FMSGANSQKQM	22633	P24310	YSLGWASFPRN	22552	P16989	PAPPTQQSSAE	22481
P49770	SELYHPDDHVL	22632	P03928	ICSLHSLPPQS	22551	P20073	YRRLLLAIVGQ	22480
Q14494	RQERKPKDRRK	22632	P36776	DEQAEALAVER	22550	P10645	VAHQLQALRRG	22480
P40313	STWINQVIAYN	22631	P78382	ETASKERVIGV	22549	P53621	IGLRISPLQFR	22476
P47989	PENCKPWSVRV	22628	P30838	YPPSPAKMTQH	22549	P48023	ESQTFFGLYKL	22476
P15813	FKRQTSYQGVL	22627	Q00975	SYHHPDQDHWC	22545	P43627	AESRSKVVSCP	22476
Q13454	HGYPYSDLDFE	22625	Q14008	KRLERIKSSRK	22545	Q16795	EDVKPAKTVNI	22476
O00548	EKDECVIATEV	22623	O95665	SGFGDPPETRT	22541	O15432	YYLAYPLLSTA	22474
Q99062	RVHGMEALGSF	22621	Q13363	EADRDHASDQL	22537	P55291	ALLPRHRGRTA	22473
Q9Y5Z0	DESSLVRHRWK	22620	P53672	GQLQSIRRVQH	22536	P26717	SSMIYHCKHKL	22473
P16410	KQFQPYFIPIN	22617	P13473	LKHHHAGYEQF	22536	Q92581	NLLDNTRHGPA	22472
Q9P286	IVPLMRQYRHH	22617	P20585	NMEETQTSLLH	22536	P33681	ERLRRESVRPV	22471
P02462	VSRCQVCMRRT	22614	Q02487	FRTLAEACMKR	22535	O00622	RLFNDIHKFRD	22469
P25103	ESFSFSSNVLS	22614	O60832	KKAKEVELVSE	22534	Q01459	WEVLKPKLLQR	22469
Q16718	EPPADQWKWPI	22613	P51513	GVRAANPQKVG	22534	P22004	RNMVVRACGCH	22467
P02765	PCPGRIRHFKV	22611	Q9UI15	FLSSSGPRVSV	22534	O60806	GGHHSPSSLDG	22467
P30084	VEKRKANFKDQ	22610	Q06330	SVTSSTATVVS	22534	O14874	HIDGREESFRI	22466
P13591	ATQTKENESKA	22609	P33765	SLDTSIEKNSE	22532	P29798	FREKKFFCALL	22466
P42336	IFHTIKQHALN	22609	P30414	HRSPSESSRYS	22532	Q14138	VWVFRLYPSTS	22465
Q92824	QLCCKTCTFQG	22609	Q02338	PGAISDMIYIR	22531	P01138	CVLSRKAVRRA	22458
Q13126	MAQFSVLLPRH	22608	Q14451	LLRHCCTRVAL	22531	Q02153	TGTEETKQDDD	22457
P48764	PPAALPESTHM	22607	P50851	QAVRLLLLGPF	22530	P34130	VCTLLSRTGRA	22457
O75936	RILRQRVENGN	22605	Q00872	ECKLEVKVIAQ	22530	P48740	DWIQRVTGVRN	22456
P15088	FIAKYILKHTS	22605	Q99784	TLFHVIRSDEL	22530	O96009	WGETAQAQFPG	22456
P48050	DNISYRRESAI	22605	P48539	KFQKKKAGSQS	22529	Q14500	EQRPYRRESEI	22455
P51589	VSHRLCAVPQV	22604	O95406	LYGMIYVLVSS	22528	P25440	SSSDTSDSDSG	22454
Q14833	QTYVTYTNHAI	22604	Q16850	NPVIRYKRRSK	22528	P16435	TKGRYSLDVWS	22453
Q9Y618	CSQYETLSDSE	22604	P29762	DVVCTRIYVRE	22528	P49023	YCQNCFLKLFC	22453
O95298	GEIFEKFHPIR	22603	Q05193	RPESPRPPFDL	22527	Q99683	KAIIDFRNKQT	22452
Q16832	IHLLLLQQGDE	22602	P06888	FGGGTNVTVVG	22526	P03915	FPLILTLLLIT	22451
P49005	DDDLGGLGLGP	22602	P56555	RDKQISASPST	22525	P57073	DQPVYTTLTRP	22451
Q16610	NISSTSEPKEE	22602	Q14978	VQVNSIKFDSE	22525	P26232	KKHIFACTGFK	22445
Q9UBI6	PFKDKKTCIIL	22602	Q15763	YCIVSAFGLSI	22523	P20929	AMEIISEKPEF	22443
Q9UHP9	SELKYVPKAEQ	22602	P10997	LKREPLNYLPL	22523	P55290	LLLSLFSLACL	22440
Q05516	IEKTYLYLCYV	22602	O15151	EIQLVIKVFIA	22523	Q9NYC9	VLAGVALLLQI	22439
Q02246	AMLILIGSLEL	22601	Q16612	RSPRISYLHFF	22523	P32247	GCSVKQAEDRF	22437
P13674	FRRPCTLSELE	22601	Q9Y6M5	NMPNKQPESSL	22523	P43005	DTISFTQTSQF	22437
O95484	GASGLDKRDYV	22600	P53999	ISDIDDAVRKL	22522	P56385	RELAEDDSILK	22436
P17039	SGSYLVQHQRI	22599	Q16527	GQGAGALVHAQ	22520	P42330	SHPNYPYSDEY	22436
P54252	IMFATFTLYLT	22598	P18669	EAVAAQGKAKK	22518	P36610	VQALSLYDGLV	22435
P14061	PELGDPPAAPQ	22597	P24311	GRVTPKEWRNQ	22515	Q02297	SVIANQDPIAV	22435
O15255	SPPPETSCVLA	22596	Q02928	LPNPCEDKDQL	22515	P56181	QPSSGRESPRH	22435
P42126	MYLERLKEEKG	22594	Q9Y5S8	KVQFYFNKENF	22515	O43741	KYVTTLLYKPI	22433
Q16698	IEELIRKTKGS	22594	P16499	GATTSKSCCIQ	22514	P15036	AILGVQPDTED	22433
Q02218	AAPATGNKKTH	22594	P51864	AGICLSIQSYY	22513	Q13415	QDDVLYALKDE	22432
P01732	GDKPSLSARYV	22592	O94760	CSVLINKKVDS	22513	P54750	TSSQKCEFIHQ	22431
P56282	KTVEDSKLQGF	22592	P56545	HGDNREHPNEQ	22512	P08034	LAEKSDRCSAC	22430
P47883	GGLSPLNSHGL	22592	Q13685	AKVFCVQRPDR	22511	Q14542	ASLSFLFKALL	22430
P20264	PPHHGLQTSVQ	22590	Q99758	HLQPPTAEEGR	22511	P14921	AMLDVKPDADE	22430
Q9UBM4	CLPRLPIGRFT	22590	Q99966	FDFTADFPSSC	22511	O43913	FDIIKYLYDFL	22430
P98187	DGLWLRVEPLG	22588	O75251	RERRLQIWYRR	22511	P31645	IPCGDIRLNAV	22429
P02792	YLFERLTLKHD	22588	P30301	GEPVELNTQAL	22508	P13686	FKTRLPRRARP	22429
Q9UBD5	DHVARLTWGGC	22586	P36957	VEDPRVLLLDL	22508	P41252	YVSVLPTTADF	22429
P28221	FQKIVPFRKAS	22585	P07315	AGSLRRVVDLY	22507	P14854	QRAEGTFPGKI	22428
P04798	CCEHFQMQLRS	22585	Q13416	TDFLEKEEEEA	22507	P53602	LGPDGLPKPAA	22428
O00337	CRFYNHTICAQ	22584	O00151	EGYEVVTVFPK	22507	O95258	ITYEQLKRLQI	22427
O75828	QLVHDKVVQNW	22584	P43116	TQSDASKQADL	22507	Q9UBP0	RWNKDFGDTTV	22427
P01133	LDPPHQMELTQ	22584	P15848	PKATGVWGPWM	22506	Q9UNQ0	YLKLLFLKKYS	22426
Q14764	PRGACTALTPD	22584	Q9UBY0	SRKARFGSEKP	22506	P14859	ASTTTTASKAQ	22426
Q9UM60	VMAMMYTVVTP	22583	P55769	SIQQSIERLLV	22506	P04220	LVMSDTAGTCY	22425
Q9UMR2	DDLDEIEKIAN	22582	P23786	FDALEGKSIKS	22505	P15086	YVASYVLEHLY	22424
P51397	PRTQHIQQPRK	22581	Q92841	PPPPPPPPSRK	22505	Q14318	VALSVVIAARN	22424
P30989	LSSNATRETLY	22581	P56856	VQSYPSKHDYV	22504	P50281	LYCQRSLLDKV	22424
P07148	DIVFKRISKRI	22580	O95182	RWELSSDQPYL	22504	P57076	HGVKDIKWRPR	22423
P20592	ALCQFSSKEIH	22580	Q13014	GQFVVRRFFKS	22503	P11279	RKRSHAGYQTI	22422
O00329	LAHNVSKDNRQ	22580	Q14197	AVKTSRRVDMD	22502	P54710	KKRRQINEDEP	22421
Q16549	DVPHGKEEQIC	22580	O96013	IVPLMRQNRTR	22500	O43181	SWNKRTRVSTK	22420
P55283	LADMYGGGEED	22578	Q00526	ARQYVLQRFRH	22499	P35869	PFPDLTSSGFL	22418
Q13114	VIVDTSDLPDP	22577	Q12860	PAFGILVYLEF	22498	P46663	RKEIFQLFWRN	22417
O96002	PRTYLYVWPYK	22575	P01588	YTGEACRTGDR	22497	P09172	PTVVSIGGGKG	22416
O76093	RSRRIRPTHPA	22572	Q92871	EIFFPETAHEA	22497	Q12841	EKTKRVSTKEI	22416
P23409	KLPCVEEVVEK	22571	P53004	EEIQKYCCSRK	22496	O15305	DTRRICELLFS	22416
Q13625	PRIKPRQRSLA	22570	P16220	ALKDLYCHKSD	22496	P11177	IIFAIKKTLNI	22415
P01189	AIIKNAYKKGE	22569	P04181	IEIINKTILSF	22496	P17707	SFAKKQQQQQS	22413
P21964	IYKGPGSEAGP	22567	Q99742	PAGTRLPRKGD	22495	P04626	NPEYLGLDVPV	22413
P48165	SSRARSDDLTV	22566	Q9UP62	VASVFYTVVIP	22495	P29033	RYCSGKSKKPV	22412

ID: ID from Uniprot Database (http://www.uniprot.org/) **Sequence**: Listed sequences match the Uniprot ID, but for the array experiments and for the computational predictions, the cysteines were changed to serine. **NA**: not available. **BLU**: biochemical light unit.

Table S1: Binding data from CAL peptide array.

ID	Sequence	BLU	ID	Sequence	BLU	ID	Sequence	BLU
P53673	FQVQSIRRIQQ	22411	Q14681	AKILQERGSRM	22359	P80365	LSPGPSPAVAR	22318
Q9Y5R6	FTVTPVIEEDE	22411	O75712	LQASAPNLTPI	22357	P80297	KGTSDKCSCCA	22318
P06746	WKYREPKDRSE	22410	P11423	ETPCYITGWGR	22357	P14222	EPPGNRSGAVW	22318
Q13145	MYSGHGKLEFV	22410	P23280	EILDYLRRALN	22356	O60635	IVSMYLYCNLQ	22318
Q14943	PAGNRSEQRGF	22409	P11479	IYQGRLWAFCC	22356	Q13639	SPLVAAQPSDT	22317
P56749	EIDIPVVSHTT	22407	O94778	DGKTRLILKAR	22355	P51685	SSRSSSVDYIL	22317
Q9UI25	FGPEVVAPQRL	22407	O15182	EEFIAIMTGDI	22355	O43708	RQPDTPTELRA	22317
P04920	VDEYNEMPMPV	22406	Q08752	KEKAVYAKMFA	22355	P01854	VQRAVSVNPGK	22316
O95500	THSGYRLNDYV	22406	Q9Y5X4	MEKLLCDMFKN	22355	Q14574	FITLAEACTKR	22315
P45877	KTPFVVEIADW	22406	Q13316	DQDDNDCQDGY	22354	P19883	YSFPISSILEW	22314
P31276	SKSKAPHLHST	22406	P32519	KQNELLEPNSF	22354	P55286	LYSVGESDKET	22313
Q15700	PFIWIPSKEKL	22405	Q9NQX5	LPAPSSPPALP	22354	P21926	CCAIRRNREMV	22313
P49639	SSTSDTLTTSH	22405	P22736	IDKIFMDTLPF	22354	P42773	ANGAGGATNLQ	22313
P14209	EPAVQRTLLEK	22405	Q11203	ARVITDLSSGI	22352	Q05923	LLQFETQVLCH	22313
Q14432	GEEIPTQKPDQ	22404	P08138	LCSESTATSPV	22351	Q99808	AVFSFLFRAIV	22312
O76082	ERPTILKSTAF	22404	Q9UKP4	LCPRPAGRVHG	22350	P55957	YVRSLARNGMD	22311
P00167	ALMYRLYMAED	22403	P27487	SHFIKQCFSLP	22350	P43166	KGRVVKASFRA	22311
O60711	CQPCFNKLFPL	22403	P55075	GSQRTWAPEPR	22350	P50570	IIRPAEPSLLD	22311
P29120	QALVDILNEEN	22403	P17844	IGYPMPTGYSQ	22349	P23610	LHLVLQETISP	22311
P47874	NLKASVVFNQL	22402	P29965	TGFTSFGLLKL	22349	P16442	VPKNHQAVRNP	22310
P00748	YLAWIREHTVS	22400	Q16619	DLGQLLPGGSA	22348	P32391	CRHNPVFGVMS	22309
Q13891	NMFTNQATVIR	22399	P05092	KITIADCGQLE	22347	O95158	YNFQSEHPYFG	22309
P42338	MAHTVRKDYRS	22399	Q02750	QPSTPTHAAGV	22347	P51689	CSCHEDGDGTP	22308
O95954	LVLDCLETRQE	22398	Q13621	GNHKNVLTFYS	22347	P30098	SSSHMSVEEGS	22308
Q16617	CGGPRPGYETL	22396	P46109	IFDPQNPDENE	22346	Q01850	RTKYRSLSSHS	22308
P51970	THGSRFYFWTK	22396	P16152	QFVSEKRVEQW	22346	P01213	PNAYSGELFDA	22307
Q08289	RKPRRVETLPT	22395	P51114	TQEAAVLNGVS	22346	O95157	YHSDTPYYPSG	22307
P21452	LLAPTKTHVEI	22395	P34059	ESIPKKCLWSH	22346	P52907	SYKIGKEMQNA	22306
Q99743	SESSGLQQPPR	22395	P46087	KFNTHFKTSGH	22346	P54098	GSLEKRSQPGP	22306
P46734	AFVKKILGEDS	22392	Q16678	VQNLQAKETCQ	22345	O00154	AKRQGHAEPQP	22305
P40199	TIGVLARVALI	22392	P20916	ELAEYAEIRVK	22345	P07320	VGSLRRVIDFS	22304
Q9UJV9	KDYLAHSSMDF	22390	P08493	NRYFRKRRGAK	22345	Q07814	DVVYNAFSLRV	22303
P03989	TVPRALMCLSQ	22389	P38432	SPSNTSSTEPA	22345	P48745	ELELKTTRGKM	22303
P46527	PKKPGLRRRQT	22389	P49753	GGREGTIPSKV	22345	P31146	LDRLEETVQAK	22302
P21728	QPITQNGQHPT	22389	P16278	QKNKDSWLDHV	22344	P49137	ARALEAAALAH	22302
P47884	GRVLWRPFQRP	22389	P53567	NTTADGDNAGQ	22344	P22033	EKCLEKKQQSV	22302
P14091	NNRVGLAPAVP	22388	Q9NYV4	PRGGRGRGVPY	22344	Q9Y2T1	GRILGKVERID	22301
Q04760	ILNPNKMATLM	22388	P56278	QELLLKLLPDD	22344	P19801	PFSYNGTYRPV	22300
Q13405	ELKAWLLEKGF	22388	Q13823	KHKRKKFRQKQ	22344	P01037	RSLVKSRCQES	22300
P54845	PGSGDPSHLFL	22388	P00367	KVYNEAGVTFT	22343	P01772	WGQGTPVTVSS	22300
P57679	SKKMLKRRSNL	22387	P26715	SSIIYHCKHKL	22343	P13535	SREVHTKISAE	22300
Q01959	RQFTLRHWLKV	22387	P35270	KSGAHVDFYDK	22343	P51946	EWTDDDLVESL	22299
P21145	AVFSLIRWKSS	22386	O75914	AAKEAIKNSSR	22342	P23284	EVEKPFAIAKE	22299
Q9Y236	FVERGGGDGIA	22385	P16885	SSCTRRNATRG	22342	Q9UHI6	IYLQEMMHSNQ	22299
P18146	ATFSPRTIEIC	22384	P25232	RGRTVGVSKKK	22341	O60683	PQKLIYLRHYR	22299
P48728	PFVPTNYYTLK	22384	P16260	YELMKQFFHLN	22340	O60423	SFWLFRMPTSA	22298
O95644	RNDLSSTSTHS	22383	P14621	KLEYSNFSIRY	22339	P43251	AALYGRLYERD	22297
O15075	SETVRSPNSPF	22382	P17661	SEATQQQHEVL	22338	O15296	DPPLIENSVSI	22297
Q92796	HYIWVPSPEKL	22382	P49448	KVYSEAGVTFT	22338	Q04900	HTRNYIPDLKK	22297
P25713	EAEAEKCSCCQ	22382	P19634	GEGEPFFPKGQ	22337	P37059	LRMPNYKKKAT	22296
Q15332	TVHRQPSHFPR	22381	P19235	EPLPPSYVACS	22335	P28340	RRFGPPGPEAW	22296
Q13206	ELVLHLLRSQS	22381	O43900	RQARDKNCIVA	22335	Q04743	SPEEIDVTSDD	22296
O60242	DVQEGDFQTEV	22380	Q16658	ETVDPASLWEY	22334	Q9NPA2	LPLLVGGVASR	22296
P35613	KGKNVRQRNSS	22380	P48751	QDEYNELHMPV	22332	P43628	IVYTELPNAEP	22296
P27482	YEEFVRVLVSK	22380	P28476	FFNLIYWSVFS	22332	Q14246	LSSMPSASKTG	22295
P55268	NLQVQIYNTCQ	22380	P49959	MNTSSLRRNRR	22332	P16519	ERSLKSILNKN	22295
P49116	EYNGQITGASL	22380	Q07108	NLYWICNKPYK	22331	Q13615	GQQKEFGVGYI	22294
Q07973	SRELPIAFCQR	22379	P02144	ASNYKELGFQG	22331	O14576	SEEEGTVELSA	22293
P55083	LKRTEMKIRRA	22379	P54707	PGSWWDKNMYY	22330	P35580	DVNETQPPQSE	22293
O43174	PARFTHFHGEI	22378	Q92874	FPVEVTLKFHR	22330	P21397	VLYKYKLLPRS	22292
P46379	PNAQRAFADDP	22377	Q16706	MEISTFRIQLR	22330	Q07864	WLLQKNPQLGH	22292
P00742	APEVITSSPLK	22377	P55056	PRLVCGDKDQG	22329	O14641	GNPSEFFVDVM	22292
Q9Y2A7	AVYKQSVTSSA	22376	P48681	GDRESWSSGED	22329	P14625	AKESTAEKDEL	22292
P05323	HVTRRTPDYFL	22376	O43678	RALENVLSGKA	22329	P51511	LYCKRSLQEWV	22292
P98173	EGCIPRRSTAS	22375	P07316	VGSLRRVMDLY	22328	P03971	PNMVATECGCR	22291
O43852	FGEALVRHDEF	22375	P18282	SLIVAFEGCPV	22328	Q13203	CRLEVKASAAH	22291
P41143	PSDGPGGGAAA	22375	P14174	ANVGWNNSTFA	22328	O43583	DSIEDLGEVKK	22290
Q06430	SETAIQPSWYF	22374	P31641	KPTHIIVETMM	22328	P81272	TAYSCLPGKPK	22290
P31513	ILLIAVFLVLT	22374	P02747	SVFSGFLLFPD	22327	P09466	DLKQMEEPCRF	22290
P78559	DESFPACKIEF	22374	P12830	LADMYGGGEDD	22326	Q08495	RNELKKKASLF	22289
P02730	RDEYDEVAMPV	22373	P10632	PPSYQICFIPV	22326	P00451	EVLGCEAQDLY	22289
P78352	PYIWVPARERL	22373	Q14031	VSRCQVCMKSL	22325	Q92686	RGGAGGGPSGD	22289
P18509	VKNKGRRIAYL	22373	P04114	KLAPGELTIIL	22323	Q13291	TVYASVTLPES	22289
P32856	IVLIIGLSVGK	22369	P54259	SHLKKESDKPL	22323	P15289	PACCHCPDPHA	22288
Q06455	GTPSTIETTPR	22369	P29371	SSPYTSVDEYS	22323	O00305	GGYSHDSRHRL	22288
O75489	LEAGDKKPDAK	22369	Q9Y678	LPVDIILASVG	22322	P09131	HFIYSSLFPVP	22288
Q13698	GSQETLIPPRL	22367	P05093	AWREAQAEGST	22322	P49711	TPEMILSMMDR	22287
P48066	IAAITEKETHF	22367	P34820	RNMVVKACGCH	22321	P54368	FERESSGEEEE	22287
P08581	DTRPASFWETS	22366	P02593	YEEFVQMMTAK	22321	P35609	FSSALYGESDL	22286
P10243	ATSSTSRALIL	22366	P51648	VAAVLVKAEYY	22321	P49335	TVKTDTSCHDL	22286
P17302	SSRPRPDDLEI	22365	Q15761	DLVSLIHCLHM	22321	O75881	SDVLFRYKVKS	22286
Q16643	PEEEEGFEGGD	22365	Q01968	LLGFLLGSEED	22321	P35221	ALSEFKAMDSI	22286
Q08554	FRTLAKTCIKK	22365	P55000	CCFRDLCNSEL	22320	P55011	GNHQSVLTFYS	22286
P32889	DWLSNQLRNQK	22364	Q13873	MVSKDIGMNCL	22320	P08206	FVVHMKQKGKK	22286
P09884	SKLFAGCAVKS	22361	O14936	APQWVPVSWVY	22320	Q92542	IAPREPGAVSY	22285
Q14126	TKHSTVQHSYS	22361	Q9Y6H8	SGRARPEDLAI	22319	P10643	TSIRPCAAETQ	22284
P18078	MASLQMAGNPG	22361	P52895	GPPNYPISDEY	22319	O60584	SCFSPYSFVSV	22284
Q09013	VWRRPGAARAP	22360	P28356	KMSKEKCPKGD	22319	O15303	PPKGEDAEAHK	22283

ID: ID from Uniprot Database (http://www.uniprot.org/) **Sequence**: Listed sequences match the Uniprot ID, but for the array experiments and for the computational predictions, the cysteines were changed to serine. **NA**: not available. **BLU**: biochemical light unit.

Table S1: Binding data from CAL peptide array.

ID	Sequence	BLU	ID	Sequence	BLU	ID	Sequence	BLU
P55055	PLLSEIWDVHE	22283	Q99440	VSGAHSTAVHQ	22254	Q13618	PEDRKVYTYVA	22222
P30049	EANEALVKALE	22282	P49901	HSPQNESRPSK	22254	P21917	NVFRKALRACC	22222
P10768	HIRHHAKYLNA	22282	Q13409	AEEEAATRIPA	22252	P16050	RPSVVENSVAI	22222
P41162	EGLAIRNASLT	22282	O95704	FRLKPSLLHMP	22251	P41227	KDSSEASDSAS	22221
Q10567	VYQAYETILKN	22281	P40121	PIFKQFFKDWK	22251	P04141	IPFDCWEPVQE	22221
P10415	CITLGAYLGHK	22281	P98082	MYRDPFGNPFA	22251	P57721	LTSEVTGMGTL	22221
Q99615	SGPGNFFFQFG	22281	Q9Y4Y9	MLVPGGEGPEV	22251	O96008	KFQCGFGLTIG	22220
O14579	DRLVLQYAPSA	22280	O00462	SFHVTSLTDIY	22251	O43612	ASVAPGGQSGI	22220
O43868	CCGFYNNTVCA	22279	P16444	LAPLVLCLSLL	22251	Q9UBX1	VNTMASSAVVD	22219
P47889	AVGIFNTVINP	22279	P36871	RTGRTAPTVIT	22251	P23528	SAVISLEGKPL	22218
Q02410	LLTAQEQPVYI	22278	O15068	ELVQEGDEGLW	22249	O60879	RSRHNGAISSK	22218
Q16878	PNATSGSLENN	22278	O95190	YPLDQNLSDED	22249	Q15701	GQVAILLFKSG	22218
O60344	PMNPGQLCEVW	22278	P36542	IEIISGAAALD	22248	Q13183	PSLANTTTPSP	22218
O75891	YLRVKTVTFEY	22278	P20248	SLLNPPETLNL	22247	O75380	YCGLQFRQHHH	22218
P18054	KPSCIENSVTI	22278	O14983	YLEDPEDERRK	22246	O15527	KRRKGSKGPEG	22218
O43861	LSPPSYCKLAS	22277	P17544	IMTPQSQSAGR	22246	O60629	ESAHDPGVVGT	22217
Q00765	VNLLGEEKKST	22277	Q15800	EKRKKFEKKTE	22246	O43405	GICRDFLESQQ	22217
Q15363	LKRFFEVRRVV	22277	P31512	MSPYLVSLWRG	22246	P33908	KDKKEVEIREE	22216
P05813	SQIQSIRRIQQ	22276	P04431	TFGQGTRLEIK	22246	P04080	NKAKHDELTYF	22215
P19113	QLPCCPLQAMV	22276	Q9Y6K9	TLQIHVMECIE	22246	P35573	IATILETLYDL	22215
P02794	KHTLGDSDNES	22276	P08235	GNAKPLYFHRK	22245	Q13098	ANSQSRMSTNM	22215
P16150	DEPEGGDGAAP	22276	Q9UI09	QEWIPPSTPYK	22245	Q14324	AECKLEVRVPQ	22214
P01121	QNGCINCCKVL	22276	Q13892	EEVTGSCYFLL	22244	Q16236	PKSKKPDVKKN	22214
P48634	GDKEPGLPPPR	22275	P01880	EVSYVTDHGPM	22244	O43524	KQASSQSWVPG	22213
O75363	PVSIGPVGKPK	22275	O60663	LYSMQSSYFAS	22244	P01708	FGTGTKVIVLG	22213
O15374	PLAERWKNSLT	22275	P46934	IENTQGFDGVD	22244	O15457	RTEQVPEKTEE	22213
Q99884	REIAEEEESMM	22275	P01127	DKTALKETLGA	22244	Q13402	MSKQRGSRSGK	22213
Q9P0J1	HVVGAYQNQEK	22274	Q13627	VCVQQSPVASS	22243	P09871	TMQENSTPRED	22212
P26196	YHSEPVEDEKP	22273	Q02818	RLPEVEVPQHL	22243	Q16875	SRSSADSSRKH	22212
P35556	ALRMRLQIQLY	22273	Q13561	SIDERMKKLGK	22240	P42702	FTNFFQNKPND	22212
P13995	LKSKELGVATN	22273	Q92633	AGVHSNDHSVV	22239	P09917	SPDRIPNSVAI	22212
P46531	FLSGEPSQADV	22273	Q9Y216	CFVNLFSVLIS	22239	P52961	VRAFPDGPGLL	22212
Q15582	VYQKLLERMKH	22273	O43847	QQHSTFSPTIK	22239	P09086	GLWWNPAPYQP	22212
Q15744	ANLIKGVGGCS	22272	Q93084	RNHMHEEMSQK	22238	P23490	QQKQAPTWPSK	22211
P07359	TVSIRYSGHSL	22272	Q13890	SIDHKPVLQMV	22238	P48645	PRNGRRSAGFI	22211
P32121	MKDDDYDDQLC	22271	P29279	LYYRKMYGDMA	22238	P35606	DINLDEDILDD	22210
P15559	IPTDNQIKARK	22271	Q9NYJ7	FPYPSSILSVK	22238	Q13421	VLALLLASTLA	22210
O15118	KGTERERLLNF	22271	P47944	KGGSDKCSCCP	22238	O95167	QGPSLEWLKKL	22210
P16581	DGSYQKPSYIL	22270	P18089	ILCRPWTQTAW	22237	P25705	IVTNFLAGFEA	22209
P35659	FIKTTVKELIS	22269	O60883	SPPLLPLGTPC	22237	O15197	QHLRQQGSVEV	22209
O15375	KQTALGWNSPT	22269	P24522	QWVPVINLPER	22237	P20591	QARRRLAQFPG	22209
O94916	QNQGNNLTGSF	22269	Q13939	AKLPCKILQRI	22236	Q9Y2N3	LQARRQHTRKK	22209
O95169	KEPERVVHYEI	22269	Q13155	APFNTALKLLK	22236	Q99457	GKHYGNKKYRK	22209
P28566	FKKLIRCREHT	22268	P03905	LNPDIITGFSS	22236	Q9Y585	LQKLFSKRISS	22209
P16587	DWLANQLKNKK	22268	P48039	TNNNVVKVDSV	22235	Q99767	LLTGQETPLYI	22208
O14640	GNPCEFFVDIM	22268	Q13323	LLSGGLHLLLK	22234	Q16558	NQYLSILAAQK	22208
O15243	GRGDDFSWEQW	22268	Q15059	SGSSSDSSDSE	22234	P41214	FSCCPEDDNWT	22208
P26441	GSHYIANNKKM	22267	P40879	RVYEVPVETKF	22234	P46821	DESFPACKIEL	22208
P50416	TLFGLSSNSKK	22267	P00488	LDVQIQRRPSM	22234	P40925	KESAFEFLSSA	22208
Q13616	DGEKDTYSYLA	22267	P80188	FPVPIDQCIDG	22234	Q15019	DGDGGALGHHV	22208
P36575	KAVEAEGDEGS	22266	O96014	CERTVERYVCK	22234	Q15678	LIQFLQNSRLI	22208
P22003	RNMVVRSCGCH	22266	O94973	QRLCELLSAQF	22233	P15924	SYSFSSSSIGH	22207
Q14123	IQNISHNWNRK	22266	P07858	IPRTDQYWEKI	22233	P20794	QPPYTDYVSTR	22207
P02795	KGASDKCSCCA	22266	P10747	APPRDFAAYRS	22233	P21359	SFKRNSIKKIV	22207
P21554	SVSTDTSAEAL	22265	Q9NPJ1	LDLSYVIEDKN	22233	P52594	PTGSSSTNPFL	22207
P16870	WWKMMSETLNF	22265	Q16515	TTLGTLEEIAC	22232	Q10472	LLRNVTLPEIF	22207
P28907	NPEDSSCTSEI	22265	O14786	KLNTQSTYSEA	22232	P39188	WDYRREPPRPA	22206
P80370	TFSKEAGDEEI	22265	P11216	LQIPPPNIPRD	22232	O43822	LRGEEVQEHAE	22206
O00754	LASVQWKEVDG	22265	O60479	PGPPPNPGAVY	22231	P04733	KGASEKCSCCD	22206
P11137	DVTAALAKQGL	22265	Q99501	LAPGGSDPAGG	22231	Q01538	SIKQAVRGIQV	22206
P54821	SLQRNQVPTVN	22265	Q99748	HELSARECACV	22231	P19474	LNIGSQGSTDY	22206
P56693	EQPVYTTLSRP	22265	P26842	DYRKPEPACSP	22230	O15169	EKIIGKVEKVD	22205
Q9Y600	LNELERLGQDL	22264	Q9NRM1	DQVQDCLLLQA	22230	P56945	FRRVLGQLAAA	22205
O95214	GSKDDFSWQQW	22264	P56470	QGDVTLSYVQI	22230	Q9Y3R5	IEYDFLEHPEC	22205
O60462	KMNHQKCCSEA	22264	P03897	EWLQKGLDWTE	22230	P15502	CLGKACGRKRK	22205
P25929	KINNNDDNEKI	22264	P78562	MNRGMDSCRLW	22229	O75376	AQYETLSDSDD	22205
O95248	WDFYTEETLAE	22263	P10523	EGKRDKNDADE	22228	O00170	DKARFRGIFSH	22204
P56975	NEIQRDSALTK	22263	P21860	RLFPKANAQRT	22228	P55081	ERPSAKKRKTT	22204
O15123	ATTMMIRPADF	22262	O15354	MSTFASVGTHC	22228	Q15406	LHSCKTSVGKE	22204
P19835	KEAQMPAVIRF	22262	P80303	PAGELKFEPHI	22228	Q13177	MAAKEAMKSNR	22204
P56557	YKFGRTEELWT	22262	P40205	EGRPPCLKINK	22228	P26442	ISLSRLDLGSG	22202
P11717	HDDSDEDLLHI	22262	P17029	DAFGAFLKSCV	22228	P09525	KVLLVLCGGDD	22202
Q12931	SCWWIRYTRTP	22262	O95415	RRCPNCGATFA	22227	O15144	KTITGKTFSSR	22202
P08709	RPGVLLRAPFP	22261	P55285	MYGGVDSDKDS	22227	Q16877	EEALVTVPAHQ	22202
P52179	LESLKGGKKAK	22261	Q06432	WESCMDAEPEH	22227	O00743	IPPRTTTPYFL	22202
P09564	CNTLSSPNQYQ	22259	Q9Y233	SVAQKAAASED	22227	Q01064	EDEHNQNGNLD	22201
P29466	LTRCFYLFPGH	22259	Q12887	SGGGDAGPPPS	22227	Q04828	GPPNYPFSDEY	22201
O00451	VLSVLMLKQAL	22259	Q08447	FKELKGGCVIS	22227	P57054	FLAAKELYTKN	22201
P10606	HYKLVPQQLAH	22258	P45983	EAAAGPLGCCR	22226	P48664	SRGRGGNESAM	22201
P08567	KAIQMASRTGK	22258	P09417	EGRTELTPAYF	22225	P53796	ERRGESTMSAH	22201
P19022	KLADMYGGGDD	22257	P51452	DRLAKEGKLKP	22225	P30954	ALCRAVGGKFS	22201
Q13606	EKVLRSKVDSS	22257	P01817	WGQGTLVTVSS	22225	O60231	KKIGKTREELG	22200
P04040	SHLAAREKANL	22256	P52701	VHKLLTLIKEL	22224	Q92988	HSSDVLSPQMM	22200
P21918	KITPFTPNGFH	22256	Q92838	GAIRLGEAPAS	22223	P21741	KAKAKKGKGKD	22200
P01706	FGGGTNLTVLG	22256	P47869	LNREPVLGVSP	22223	P81274	FKNSGKKSADH	22199
P47886	GVGVFNTVINP	22256	P15248	KEKMRGMRGKI	22223	O00222	TTYISYSNHSI	22199
P24043	HWRLILPRPWN	22255	P10244	PSHTSRTLILS	22223	P01163	QPPGGSKVILF	22199
P23946	RPWINQILQAN	22255	P43629	AKPRSKVVSCP	22223	Q16633	ALNHTLSVEGF	22199

ID: ID from Uniprot Database (http://www.uniprot.org/) **Sequence**: Listed sequences match the Uniprot ID, but for the array experiments and for the computational predictions, the cysteines were changed to serine. **NA**: not available. **BLU**: biochemical light unit.

Table S1: Binding data from CAL peptide array.

ID	Sequence	BLU	ID	Sequence	BLU	ID	Sequence	BLU
P20774	CLKRLPIGSYF	22199	P32249	MMIHSKSSNGK	22169	P31785	APPCYTLKPET	22148
P50443	CVPNGLSLSSD	22198	Q99687	FGTRKEEWHYL	22169	Q02817	ARRSPRHLGSG	22148
P49685	FARRRKRSVSL	22198	P43366	RAPFSRSSHPM	22169	P57740	GLDPLGYEIQL	22148
P11055	SSRMVVHESEE	22198	Q07687	GGAPVSAGTIF	22168	Q9UPR5	SLEAYCHIRGF	22148
P48723	PNKHLQKTNFN	22198	Q92949	LQDWASVGAFL	22168	P13688	TEIIYSEVKKQ	22147
P13945	QRLDGASWGVS	22197	Q15080	KDNYRVYNTMP	22168	P23511	EEAMTQIIRVS	22147
P11161	LAPCSSRTRTP	22197	Q06141	VRLPYVCKFTD	22168	P12829	YEAFVKHIMSG	22147
P08473	YMNPEKKCRVW	22197	P48553	QDDHVLEVSVT	22167	P12882	REVHTKIISEE	22147
Q15777	IIFDLPNPQGS	22196	P21439	MVSVQAGTQNL	22167	P07197	HAIVKEVTQSD	22147
P31751	PQFSYSASIRE	22196	O95222	DPDPKKGSRNV	22167	P47887	VICKRKNPFLL	22147
P05423	DFESLLDHKHR	22196	Q08211	YFGQGRGGGGY	22166	P50458	SPSQTTLTNLF	22146
O75665	GFSHEELDDSW	22196	P56211	KPSLVASKLAG	22165	P03956	KANSWFNCRKN	22146
Q14210	SLLAVILAPSL	22196	P35226	SVNGSSATSSG	22165	Q9UIE0	DIILSLFLNDM	22146
Q14566	LVVNPNYLLED	22196	P20807	VLEWLQLTMYA	22165	P19876	IEKILNKGSTN	22145
Q13368	DTHWVPVSWVR	22196	Q13620	KENPNQYNYIA	22165	Q13330	PVNDEPIVIED	22145
Q9UNT1	TPSEEAASPHS	22196	Q9UBR4	SWLDEVDHAQF	22165	P80520	NALMDGASGLM	22144
P53396	ISYVLPEHMSM	22195	O43318	RSQQQKRQGTS	22165	P07741	PVPFFSLLQYE	22144
P19878	TTDLESTRREV	22195	O00273	QNPKRARQDPT	22164	P33151	YGSDPREELLY	22144
P17752	ALAKVSRKPSI	22195	Q99504	SLHQALELDFL	22164	P24046	LFNLIYWSIFS	22144
Q92747	LESSIQGLRIM	22193	P04209	FGGGTKLSVLG	22164	P80421	WGQGTTVIVSS	22144
P29474	FDPPGSDTNSP	22193	O95382	RAGSTPVTSGP	22164	P43356	LHERALREGEE	22144
P05026	GRFDVKIEVKS	22192	Q92794	KQSLNGPYMRR	22164	Q9Y6L6	PSAGADSETHC	22144
P49327	SLAEPRVSREG	22192	P03891	LPISPFMLMIL	22164	Q15118	EPKDMTTFRSA	22144
Q02556	SFFRENQQITV	22192	P51861	IDWKTWIWWKT	22163	Q9Y3A4	LLRAQRKFRPY	22144
P08183	MVSVQAGTKRQ	22192	Q07507	MTEYDCEFANV	22163	Q15915	ALSSNFNEWYV	22144
O15382	GIRAHEWMFPV	22191	P42566	IALSKSEISEA	22163	P06731	MIGVLVGVALI	22143
Q01638	LSRKNPSKECF	22191	Q14773	LCKCLAMKSQA	22163	Q99661	SRQISSKKRPQ	22143
P25388	VRVWQVTIGTR	22190	P43489	QADAHSTLAKI	22163	Q13164	LLADLPDLQDP	22143
Q99542	TLSATETTFEY	22190	P78363	RAAGASRQAQD	22162	P14543	NTLGVDCIERK	22143
O76099	FFNGDITAGLS	22190	Q16671	NPQPACTLSPV	22162	O43272	LRTGNLFHRPA	22143
P56134	HLKHERLRKYH	22189	Q9Y5R5	YSYYVYIMNHL	22162	Q15825	LQPLLGNTGKS	22142
P20849	LERLTAAWLSA	22188	P51512	LYCKRSMQEWV	22162	P20265	PPHHGVQTPVQ	22142
P55273	DILQGHMVAPL	22188	P05408	PHFSDEDKDPE	22161	Q16674	VKTDKWDFYCQ	22141
P49281	NTMDADSLVSR	22188	P36894	AKMVESQDVKI	22161	Q00059	KKQRKYGAEEC	22141
P45984	DASTGPLEGCR	22187	P07438	KGSSEKCRCCA	22161	Q09666	EVELSVSTKKE	22140
P26038	TKQRIDEFESM	22187	O14841	WWMFILGAFGA	22161	Q15696	RDRTVQSPKSK	22140
P15291	TQITVDIGTPS	22187	P28332	EAVELMKTGKW	22160	P35670	LLNGRDEEQYI	22139
P12004	LAPKIEDEEGS	22187	O75508	SSPTHAKSAHV	22160	P08185	SLFLARVMNPV	22139
P08319	RCQEQFRILSD	22186	O14647	NLCQELFLGRK	22159	O95471	YPKSNSSKEYV	22139
O43520	GTAEYRRTGDS	22186	P45985	PATPSSPMYVD	22159	P10242	VNAFSARTLVM	22139
O76096	QHFEVPVLRCH	22186	P29475	SKKDTDEVFSS	22159	P20309	IFHKRAPEQAL	22138
Q14995	SEELLAFKVHP	22186	P15144	VVLQWFTENSK	22158	Q99643	TVLSSMGLAAM	22138
P53671	MQYGLTRDSPP	22185	P40259	KWSVGEHPGQE	22158	Q9ULX7	VFTSAQATTEA	22138
Q99102	HATPLPVTDTS	22185	P01258	RPHVSMPQNAN	22158	P11532	TPGKPMREDTM	22138
Q99733	DEDDAEINPKV	22185	P25089	SPPEETELQAM	22158	P19138	MPVPAAAGAQQ	22138
P50876	CSKGDDDPLPT	22184	P19877	ITSLEVDKAGR	22158	Q9UK45	IPNPFIQQQDA	22138
P08547	LETIILSKLSQ	22183	P12074	VNPLPTGYEDE	22157	O14770	NMGMDGQWHYM	22138
Q15388	SAQSLAEDDVE	22183	P05976	YEAFVKHIMSI	22157	P02818	QEAYRRFYGPV	22138
P55087	KDQSGEVLSSV	22182	Q15772	QCEARLEVRGE	22156	O15509	RIVAEEFLKNF	22137
Q9P0U1	PEPTVLSLLWG	22182	P12111	AKPGVISVMGT	22156	O14867	QQMTDKCTTDE	22137
P16615	TDTNFSDMFWS	22181	Q9Y217	SLEYGVARMTC	22156	P21854	ICEMTAFRFPD	22137
O14958	DNDDSDDDDDE	22181	O15130	SLAAPQRFGKK	22156	P02686	DSRSGSPMARR	22136
P39880	ASREEPIEWEF	22181	P24407	KRSSFFRCVLL	22156	Q15546	YRSPTDFMRHL	22136
P17050	YPIKNLEMSQQ	22181	P13994	PDHPAGARDGR	22155	P19105	ILKHGAKDKDD	22135
P34982	RLLDKHFKRLT	22181	P29274	DPLAQDGAGVS	22155	P37275	EQVSEEKTNEA	22135
P51858	EAPGIRDHESL	22180	O75173	ILRRRPWAGRK	22155	P11229	GSVHRTPSRQC	22134
Q92887	AGIENVNSTKF	22179	Q02221	VNPLPTGYEHP	22155	Q10570	LLETDRVTAHF	22133
P51817	PQKDLEIFKNF	22179	Q14314	KMMIRPKHFKP	22155	P17813	QSTPCSTSSMA	22133
P35443	FQTQNFDRFDN	22179	Q00444	KDSKMKSKEAL	22155	P42357	KSTKIPESEDL	22133
Q14137	SGADGTVRLFT	22179	P43631	DEQDHQEVSYA	22155	P20155	HNIKIIRNGPC	22133
P30411	HKLQDWAGSRQ	22178	Q14994	AMMPLLQEICS	22155	P54315	REDTLLTLTPC	22133
P12109	PPGHQGPPGPD	22178	P01298	AVPRELSPLDL	22155	P09848	SQQELSPVSSF	22133
P41180	GSTVTENVVNS	22178	P27105	GIIGAKHSHLG	22154	P43354	IDKLFLDTLPF	22133
P42658	TVTAKEDEEED	22178	P12271	GPQAQAENTAF	22154	P45452	RVMPANSILWC	22132
P52732	SRLPLRAQINL	22178	O15438	FYGMARDAGLA	22154	Q99550	PCHLYDYRFQG	22132
P51816	LCWLRIDAHLL	22178	O95156	YHSETPYLSSG	22154	O43462	VTLGLWMVTAR	22132
P03886	PITISSIPPQT	22178	P02649	TSAAPVPSDNH	22153	P07196	AGEEQAAKKKD	22132
P12643	QDMVVEGCGCR	22177	P22681	VSISSPAHVAT	22153	P16112	HPRRSRPSTAH	22132
Q13158	WNSDASTSEAS	22177	P11487	RLGSQLEASAH	22153	P02787	SLLEACTFRRP	22132
P30203	TIPKEGPGPAP	22176	P48029	SSKVVVVESVM	22153	P51825	FQQLQELTKTP	22131
O60673	APYLRQLLDQF	22176	P30807	FAAHSASLTVR	22153	O00358	PGGIDRFVSAM	22131
P52564	VASFVKLILGD	22176	P11678	PRLNLSAWRGT	22153	Q13257	SMVAYKIPVND	22131
P05937	DLALILCAGDN	22175	Q16763	KKRALRALRRL	22153	P01023	NAPCSKDLGNA	22130
Q14112	AVYPYCPTGRK	22175	P36406	QLVAAGVLDVA	22152	P51530	LCHILGDFQRE	22130
P32927	PWEVNKPGEVC	22174	P05305	ERYVTHNRAHW	22152	O43676	LESLNKDKKHH	22130
P50549	NPHPYNEGYVY	22174	P08574	KSRKLAYRPPK	22151	Q9Y6N1	EGHKLPVPGYN	22129
P16333	GEKLYLVKHLS	22174	P20393	EKLLSFRVDAQ	22151	O95424	SELDVFDAYLE	22129
P49447	TLRQGDSPGSQ	22173	O00483	YSKLKKERPDF	22151	O14732	QTINYGRDDEK	22129
Q12929	GVESFDEGSSH	22172	O14599	TGSVSKPRSQK	22149	P22307	NLQLQPGNAKL	22129
O60825	LRAQDMQEGAD	22172	P31040	CATVPPAIRSY	22149	O95180	PAPGGGADDPV	22128
P55160	YREVSRAFHLN	22172	O75417	ASWGELKDFDV	22149	O43143	EVIERTALKDE	22128
Q03692	SSFSGFLVAPM	22171	Q05810	VACKKACMLGQ	22149	Q16363	GAVSINSCPAA	22128
O14727	LGILYILQTLE	22170	Q99538	LSMDHVCLGHY	22149	P33992	RMQRKVLYRLK	22128
Q13790	DLDPGAGSLEI	22170	P19256	CDRKPDRTNSN	22149	Q13585	DVEDDPDEMAV	22128
P54132	RPFLKPSYAFS	22170	Q01860	VTTLGSPMHSN	22149	O15440	AAAENKVAVKG	22128
Q9Y281	NVVVSLEGKPL	22170	P35249	ATVMQQLSQNC	22148	O60356	NSERKKRGARR	22128
P30040	FQKKGAEKEEL	22170	P08133	KALLALCGGED	22148	P22792	HTGAGEGGLWG	22127
P16475	YEAFVRHILSG	22170	O96018	LLTGQEQPVYL	22148	P31994	ALEEPDDQNRI	22127

ID: ID from Uniprot Database (http://www.uniprot.org/) **Sequence**: Listed sequences match the Uniprot ID, but for the array experiments and for the computational predictions, the cysteines were changed to serine. **NA**: not available. **BLU**: biochemical light unit.

Table S1: Binding data from CAL peptide array.

ID	Sequence	BLU	ID	Sequence	BLU	ID	Sequence	BLU
Q15012	EKEPPPPYLPA	22127	P57103	GELEFKNDETV	22101	Q99967	VCKQQPSRVSC	22082
P56704	AERRREKCRCV	22127	O75607	ILPAKKQGGRP	22101	O43639	GEKLYLVRALQ	22082
Q12797	TPQQRRSLPAI	22126	P30531	AGSSTSKEAYI	22101	P43657	KSKIFDNESAA	22082
P11049	DHVYNRLARYR	22126	P11926	RAACASASINV	22100	O75153	AAKDPSPSVQG	22081
Q9Y4Z1	VVLVAPPLRVG	22126	O00757	LTCVQKNQAGS	22100	Q9Y6J6	NIGAAGFKMSP	22081
P08254	HTLKSNSWLNC	22126	Q13724	SLVLLAMAEDY	22100	P49006	GPTPASAEQNE	22081
P12883	RDIGTKGLNEE	22126	Q07820	GVGAGLAYLIR	22100	P57105	AWAFMRYRQQL	22081
P01185	EPFEPAQPDAY	22126	P35410	LPREHRVDVET	22100	Q99572	EGQYSGFKSPY	22081
O75110	FSPPSYSKLTS	22125	P18074	LKRIEQIAQQL	22100	P49788	LTSVRQWVRKT	22081
P20273	QENVDYVILKH	22125	Q05996	YLYEKRTVSNH	22100	P42127	ACSCRVLSLNC	22080
P54277	FHHLTYLPETT	22125	P21333	IPGSPYRVVVP	22099	P55008	PPAKKAISELP	22080
P51884	CLRVANEVTLN	22124	O43823	DAESKDAVPTE	22099	Q13075	WILPFSPIIQK	22080
P49279	LEEDQKGETSG	22124	P27695	DHCPITLYLAL	22099	Q9Y5A7	VENRKSATKKN	22080
P32242	KDQASWRFQVL	22124	Q9Y679	RFTERRAQEAD	22099	Q02641	LEGWGRGVYIR	22080
P09012	NNAMKISFAKK	22124	P20931	SLANMVEPCLY	22099	Q14232	AVSDELIKLYL	22080
P05451	DKFSFVCKFKN	22123	P36405	WVCKNVNAKKK	22098	P55345	KVGEKVFPIWR	22079
P26358	AKIKEEEAAKD	22123	P30042	MVRKVLELTGK	22098	Q92858	HSHYSDSDEAS	22079
O00574	HNVEATSMFQL	22122	P40692	LPDLYKVFERC	22098	P55289	EEESYNPDKVT	22079
P02746	SGFLLFPDMEA	22122	O75534	ERKIRQAGVID	22098	Q14209	DSYDLGDLLIN	22079
Q9UBR2	EEHCTFGDPIV	22122	P40394	SGQSIRTVLTF	22097	P54963	PVSQPSLVGSK	22079
Q02880	EEDDVDFAMFN	22122	P20674	STPEELGLDKV	22097	O00213	LKPKRLGAHTP	22078
O75531	GCLREWCDAFL	22121	P34903	ESAIKGMIRKQ	22097	P11912	LNIGDVQLEKP	22078
P21730	VDTMAQKTQAV	22121	P35749	DADFNGTKASE	22097	P34910	ESLPPPPAELL	22078
Q99426	FPEEDYGLDEI	22121	O95232	VCLFGNYRPHL	22097	P50895	RGGSGGFGDEC	22078
P55287	YGSKDTFDDDS	22120	Q9UBJ2	LKTIKNEDETS	22096	Q14108	TADERAPLIRT	22078
P24385	CTPTDVRDVDI	22120	P22570	DPQEMLRLLGH	22096	Q13508	SVSAINLFVAL	22078
O75381	PEGASNESERD	22119	P98194	VSSTSSSFLEV	22096	Q99435	CSVDPQCLQEL	22078
P43897	CGEGEEAAETE	22118	O60911	GIATAASYPNV	22096	Q12816	ASLGACGFSYG	22078
Q16769	LQVFVLEYLHL	22118	Q12873	HRFFKKVPEIQ	22096	P24588	SDDNKINNLLQ	22077
O00253	LGTAMNPCSRT	22117	Q9Y3B8	IENGENEKTVS	22096	P49914	DEVLYEDSSTA	22077
Q15326	AEHKRTCRRKR	22117	P42025	DGSRAIHRKTF	22095	P38571	NKIINLMRKYQ	22077
P14138	CPRCLFQEGAP	22117	P20645	SEERDDHLLPM	22095	Q13751	NGRVLYYATCK	22077
Q13451	AMEEEKPEGHV	22117	P21851	IYQVYDSILKN	22094	P36952	NIIFFGKFCSP	22077
P43363	SSATGSFSYPE	22117	O95342	YKLVTTGSPIS	22094	O15481	RATSSSSSQPM	22077
Q08209	DSNGSNSSNIQ	22117	P52888	VGGCEPEPQVC	22094	P34972	PDSRDLDLSDC	22076
Q00888	ITVKVSDWILP	22117	P32297	FLQPLMAREDA	22093	P27658	SSFSGYLLYPM	22075
P55263	GCTFPEKPDFH	22115	Q16787	GPVSLNGCPDQ	22093	Q16854	MREVNTFVKNL	22075
O00238	AKMSESQDIKL	22115	P25205	VMVSEGIIFLI	22093	Q99698	FYSFLSSYAAG	22075
P49411	MTEEEKNIKWG	22115	P56277	EENLTRKSASK	22093	O00470	NMGMEGQWHYM	22075
Q14392	RRQKFNQQYKA	22115	P16109	VFTNAAFDPSP	22092	P55157	PQPDSTSSGWF	22075
P06905	VLKLMGRGTKF	22115	Q05195	LQDSHKACLGL	22092	O95801	CYRFYSTRGTL	22075
Q01780	DRGFRYNWPQR	22115	P25189	KGLGESRKDKK	22092	P57077	RIQYQKRQGSS	22074
O95777	IRAEPLNSVAH	22114	O43805	PDQKSSGGRDS	22092	P33076	LQQQDSRISLR	22074
Q9Y4Z0	EKKPGRQAGKQ	22113	Q13216	EDAWSSSDEEG	22091	P54284	QRNRPWPKDSY	22074
P78395	EPILCPCFMPN	22113	Q16690	SRSPVATATSC	22091	Q04741	NGEDIDVTSND	22074
P42679	DGSTSPRSQEP	22113	P56270	VSSQPLPSQPW	22091	Q01740	ALLVAIFLIFL	22074
O60942	PPPPKRPRPLT	22113	Q15742	SVKVEAEASRQ	22091	Q9Y333	AARKEALQQKQ	22074
P54296	LIPASASAAGQ	22113	Q15843	LALRGGGGLRQ	22090	P25912	QSRKKLRMEAS	22074
P49790	RKIKTAVRRRK	22113	P02533	STHEQVLRTKN	22089	P35227	TVNGAPVPPLT	22073
P17677	EEEPEADQEHA	22113	P43359	ADLIHFLLLKY	22089	Q13015	ASIHSFELDLL	22072
P20292	STTISPLLLIP	22112	P22897	GNIEQNEHSVI	22089	O43687	QNGNDNENNRK	22072
P46926	KSQSSKKPYSD	22112	Q00266	FPWEVPRKLVF	22089	P39195	GVSHRARPPPA	22072
Q12889	IPENSAVDEEA	22112	P39656	FLHMKEKEKSD	22089	O43681	LLEPYKPPSAQ	22072
Q92778	NELPYICKFKV	22112	Q01954	KSLASSPSHLQ	22088	P16118	EEALDTVPAHY	22072
Q06250	PGVTQRPRTTE	22112	Q9Y463	RGVPQSTAASS	22088	P49593	PEPETQAPPRS	22072
P00813	MPPSASAGQNL	22111	P21781	KTAHFLPMAIT	22088	P07327	SGKSIRTILMF	22071
O00187	IPWIENIISDF	22110	P11047	GCFNTPSIEKP	22088	P29317	KDQVNTVGIPI	22071
P35318	APPSGSAPHFL	22109	Q07954	GPEDEIGDPLA	22088	Q99612	DHLALHMKRHL	22070
P08236	KSQCLENSPFT	22109	P43088	ESPVAEKSAST	22088	P32243	DQTSSWKFQVL	22070
P06881	AFGRRRRDLQA	22109	P04211	EYYCLLYYGGA	22087	P82251	MEVVPPEEDPE	22069
P46966	ILHLVVMNFVG	22109	O60882	SVVKSSSWIGC	22087	Q02388	VQSQGTGTAQD	22069
P13929	AGRKFRNPKAK	22109	Q29631	GSDESLITCKA	22086	P50454	RPKGDKMRDEL	22069
Q02509	GPLGIGPLHGR	22109	P42685	SSYSDANNFIR	22086	P07948	TATEGQYQQQP	22069
Q15848	FTGFLLYHDTN	22108	P04150	GNIKKLLFHQK	22086	P27361	ARFQPGVLEAP	22069
P57060	VFQMFFGVEGQ	22108	Q14802	PPLITPGSAQS	22086	Q14582	HCRRLGRPALS	22068
P06705	LDIHKKMVVDV	22108	P28482	ETARFQPGYRS	22086	P02776	YKKIIKKLLES	22068
P47224	FYVALERVSHE	22108	Q13795	VHRPPRQRDIT	22085	P05184	VESRDGTVSGA	22067
Q00007	NNLYIFQDKVN	22107	O95177	SSSRHLSRTQT	22085	Q14896	CECRLEVRVPQ	22067
O14682	AFVSTWKHLPS	22107	P11413	GTYKWVNPHKL	22085	P49257	SQQEAAAKKFF	22066
Q16655	SAEQSPPPYSP	22107	Q9Y2U5	ELLRHMFVHYH	22085	P35498	QEGKDEKAKGK	22065
P50222	DSDHSSEHAHL	22107	Q12986	EPIIDYFDVQD	22085	P27815	PGGGGSGGDPT	22065
P08172	MCHYKNIGATR	22106	P55209	KDQNPAECKQQ	22085	P28300	AYASGCTISPY	22065
Q16134	EGGGGPAYNGM	22106	O60566	KLTSPGALLFQ	22084	O96000	KAAKEAAAATS	22065
Q12866	DDSSEGSEVLM	22106	P02745	VFSGFLIFPSA	22084	P39189	LGLQAGATAPG	22064
P15382	NTHLPETKPSP	22106	Q9Y6J0	ETDEDDDYMDI	22084	Q13634	LYGEIESERTT	22064
P16299	ENHGTGNHTAQ	22106	P08217	IDWINSVIANN	22084	Q14511	FKRSLLEMATF	22064
P10275	GKVKPIYFHTQ	22105	P29322	MENGSLDTFLR	22084	O00311	LLHPFFKDMSL	22064
Q07021	LEDLKSFVKSQ	22105	P52756	KAMFARFIEME	22084	P51828	DTAKFQGLGLN	22064
P35240	AKSRVAFFEEL	22105	P80422	FGQGTDLTVLG	22084	Q99707	WLGPILGYDTD	22064
P51813	SIEPLREKDKH	22104	Q9UJ68	TGVSCPVGIKK	22084	P50406	LRPHPLGIPTN	22063
O43918	SMARPAAPFPS	22103	P13533	IGAKQKMHDEE	22084	O14753	VTSLLQGSPHL	22063
O14520	PLHESMALEHF	22103	P30876	SMSIAPRMMSV	22084	P41279	LVRGPPTLEYG	22062
Q00688	LTFEVELVDID	22103	P43681	LFLPPWLAGMI	22083	P24001	PQKCSEPQSSK	22062
Q9Y690	VALPEYHRKAV	22103	P12429	ITLLKICGGDD	22083	P51690	LCWCLREDDPQ	22061
P31327	HYRQYSAGKAA	22102	Q14831	KKYVSYNNLVI	22083	Q03701	ENMGSKFDNIA	22061
P01876	VVMAEVDGTCY	22101	Q16659	QTYSSILKHLN	22083	P48960	TRALRASESGI	22061
P09038	AILFLPMSAKS	22101	Q15014	VASAEYHRKAL	22083	Q08708	SRQNWPKGENQ	22061
P43355	EAALREEEEGV	22101	Q16645	LIFDVELLNLE	22082	O43246	QAPAQDPGHME	22061

ID: ID from Uniprot Database (http://www.uniprot.org/) **Sequence**: Listed sequences match the Uniprot ID, but for the array experiments and for the computational predictions, the cysteines were changed to serine. **NA**: not available. **BLU**: biochemical light unit.

Table S1: Binding data from CAL peptide array.

ID	Sequence	BLU	ID	Sequence	BLU	ID	Sequence	BLU
P29966	SPEAPPAEAAE	22061	P03901	DYVHNLNLLQC	22041	O14519	CLAETERNARS	22023
O60568	TRYIMVSFVDP	22061	P51575	TLGLQENMRTS	22041	Q99518	VVAFFCQLQWS	22023
Q01742	DRILTLTTNEI	22060	Q04771	DNSLDKLKTDC	22040	P57078	PAATLLRRSKT	22022
Q93088	ELFEKQKFKSQ	22060	P11150	IKSKTSKRKIR	22040	P41211	LQCDPSSRSQF	22022
P12645	PNMTVESCACR	22060	Q13469	EFSGPPARNQT	22040	Q13506	KVIKTEPEDSR	22022
P08686	GMGAHSPGQNQ	22060	O95867	SLAGLGLWLLH	22040	Q14973	YKGEDCSPCTA	22022
P22607	PAPPSSGGSRT	22060	Q02952	RESAKSELTES	22039	Q9Y5P8	YACGDEDLEPL	22021
Q14152	TDEDGWTTVRR	22060	P02760	DGDEELLRFSN	22039	P07451	INNRVVRASFK	22021
P14649	YEAFLKHILSV	22060	P21453	IMSSGNVNSSS	22039	Q13617	QASADEYSYVA	22021
P51810	GDPALPTHGDL	22060	Q03111	KLQSCLEAVAT	22039	P43357	LHEWVLREGEE	22021
O00459	APGPGPPPAAR	22060	P12259	LRLELFGCDIY	22039	P33316	GSGGFGSTGKN	22020
P24941	DVTKPVPHLRL	22059	P09960	AMLVGKDLKVD	22039	P54886	ENLPIPQRNTN	22020
P78369	PSKQFDKNAYV	22059	O75342	DPVLIENSISI	22039	Q13191	FPPPVSPRLNL	22019
Q14894	KLIYDSWSSGK	22059	Q15233	AEFAPNKRRRY	22039	P08246	PRDPDPASRTH	22019
O14733	VLSQPHLPFFR	22059	Q92870	LKQKRPVTEMP	22038	P49771	PSPQDLLLVEH	22019
O60885	SDSEDSETGPA	22058	P07355	KALLYLCGGDD	22038	Q13084	PAVVQKTASGQ	22019
Q16739	CGGTAEEILDV	22058	P55899	LKDVNVIPATA	22038	Q16798	WPKEAMNVQTV	22019
P56556	FLSKFYVGHDP	22058	O00566	RQDISVHKLKL	22038	Q10571	NRFGTFVAALT	22019
P02768	LVAASQAALGL	22057	O75414	YVAGTGGLGPA	22038	P49354	TENDSPTNVQQ	22019
P31415	NTEDDDDDDDD	22057	Q02640	SVVPQEQEHAM	22037	Q06190	KCGKLQSVDEE	22018
P24572	YEELVRMVLNG	22057	P12980	MEQTALSPEVR	22037	P13928	NALLSLVGSDP	22018
Q12908	KANGGFQPDEK	22057	P53985	EGGPKEEESPV	22037	Q9NVD3	ETLHSLQTAFT	22018
Q15517	GKIPCRSIRIS	22056	O75694	KSLQAKLERLH	22037	P00533	VAPQSSEFIGA	22018
P46094	GAFAYEGASFY	22056	P19971	PFAELVLPPQQ	22037	O00167	ALRHALELEYL	22018
Q14117	ATAGTRKQAHP	22056	P23109	NLIAEGLKSTE	22036	P50221	DGDSTASPSSE	22018
O43237	MVTNSSTENEA	22056	Q07075	REWFFNLLESG	22036	O15297	LHQHRKTVCVC	22018
Q12778	VKTTTHSWVSG	22056	P04083	EKILVALCGGN	22036	P54278	HIANLGVISQN	22018
O15234	CTKCFSTPKGR	22056	P24863	NGSQNSSYSQS	22036	P27338	LAHKRGLLVRV	22017
Q99547	KAKKMFLKPQD	22056	Q9UJX0	GLTLQWILTSR	22036	Q99250	DKGKDIRESKK	22017
P43630	RAPQSGLEGVF	22056	P24390	VLKGKKLSLPA	22035	P23368	ESASSPPVITE	22017
P19404	KGPGFGVQAGL	22056	P07954	WVKPKDMLGPK	22035	Q99218	ATDKTKQEEVD	22016
P51116	LELGSMVNGVS	22055	P48742	HPPEMNEAAVW	22035	P43320	WHQRGAFHPSN	22016
P28845	TSYNMDRFINK	22054	P33527	FYSMAKDAGLV	22035	P41217	GELSQGVQKMT	22016
P55084	GHAMIVEAYPK	22054	O15294	MIKPVEVTESA	22035	P41231	AGSENTKDIRL	22016
P49326	LAFFAIIIAYF	22054	P57739	EFNSYSLTGYV	22034	O15143	LESALKDLKIK	22015
P08758	KALLLLCGEDD	22053	P80419	WGQGTLVSVTS	22034	Q14508	CGKVSCVTPNF	22015
P26998	KWHKRGRFPSS	22053	P43360	LHEWALREGEE	22034	P01229	DHPQLSGLLFL	22015
P54760	PGGTGGPAPQY	22053	Q9NZN9	PPPSPGHSLQH	22033	Q9Y5N6	NAASAQKATAE	22015
P48304	KKFSFVCKFKN	22053	P15090	GVTSTRVYERA	22033	P30532	LIPVHIGNANK	22014
P43358	EAALLEEEEGV	22053	P21462	LPSAEVALQAK	22033	P55064	ERKKTMELTTR	22014
Q9ULC6	PFPFKWWNMVP	22053	O00255	LSFLKRQRKGL	22033	Q08462	SRSLSQSNVAS	22014
Q13507	EKLNPSMLRCE	22053	P49286	IIGVQHQADAL	22033	O15480	SKAKASSSSHA	22014
O43315	KPEKYELSVIM	22052	Q15013	QAPVTFKGFRE	22033	O75027	NSVKGCGNCSC	22013
P54317	EENVLQSLYPC	22052	P35414	IPYSQETLVVD	22032	Q9UP79	AKPCESQLCPL	22013
P22676	LEIVLCSEPPM	22051	Q14596	LNNNDWYSQRY	22032	P55201	GEQSSETSDSD	22013
P47928	VNKQGDSILCR	22051	O43772	MKFLNWATPNL	22032	O43306	TTYFLNGGPSS	22013
P11226	TSHLAVCEFPI	22051	P00505	YLAHAIHQVTK	22031	P23515	LLLNVVVMLAV	22013
P50548	TAQLSLEHRDS	22050	P01124	SLYSLQFAGGN	22031	Q9Y5K3	SSMSEGDEDEK	22012
P50440	RRRGTLQSYLD	22050	P56696	SISRSVSTNMD	22031	P31937	VFQFLREEETF	22012
P05013	RNLQERLRRKE	22050	Q13342	VDAESIQQMAP	22031	Q9UBQ6	GFPYANYKRKI	22012
P56642	NANAYFQSLIK	22050	P48163	EVQKIQTKVDQ	22031	Q13505	TLGMAEEDEEE	22012
P32245	LGGLCDLSSRY	22050	P41218	KKNKEGPMNVN	22031	P21810	RLAIQFGNYKK	22012
Q9ULZ9	ALWTAAQALTL	22050	P47898	SAFKNFFSRQH	22030	P18582	LCCGIRNSSVY	22011
P49321	EAGATVESTAC	22050	P35368	KSNMPLAPGQF	22030	P21291	GQGAGALVHSE	22011
Q14956	LLKNQEFKGVS	22050	P20648	PGSWWDQELYY	22030	P29692	QSVDIAAFNKI	22011
P07204	HVRTERTPQRL	22050	Q09161	AVFQQFCALQA	22030	P37286	FSEDEAQDMEL	22011
P49918	SVEQTPRKRLR	22049	Q16204	LLGPELHSPGF	22030	O43731	KGKKLSLPMPI	22010
P09603	TQDDRQVELPV	22049	Q14204	ITMNPGYAGNS	22030	O75352	AKPPHKQKKAQ	22010
Q05397	RLKMLGQTRPH	22049	P01774	FAHYGQGTLVT	22030	Q92934	NLGRGSSAPSQ	22009
Q13753	GCYNTQALEQQ	22049	O00482	LLIEMLHAKRA	22030	P26992	AAAATASSLLI	22009
O75439	QIRSNMCWLRD	22049	P35070	INEDIEETNIA	22029	Q93063	KLKSFPNIGSL	22009
O14678	GRWELMRIKVE	22047	P24903	PRPFQLCLRPR	22029	P53667	SGLPAHPEVPD	22009
P21709	RILCSIQGFKD	22047	P42892	PMNPPHKCEVW	22028	Q9Y6G1	MMILRLVLLLL	22009
O43262	LNTIKKEKKMT	22047	O43320	PSMSRDLFHYR	22028	P78325	RKQGAGAPTAP	22008
P15018	KQIIAVLAQAF	22047	Q16653	GQFLEELRNPF	22028	P26438	GLTWLTSNYKS	22008
P29803	ANPWIKFKSVS	22047	O00339	MEALENRLRYR	22028	P98198	PSGGADKPLKG	22008
P30825	RTPDGNLDQCK	22046	Q9Y5B8	EVQYFFKILDN	22028	Q99653	VEQKMSIRFLH	22008
P56178	HPLALASGTLY	22046	O00623	QHLIKLYSPEN	22028	P00001	LIAYLKKATNE	22008
P08218	NDWINSVIANN	22046	Q13796	LLDSLQPERGK	22027	P19957	GSCGMACFVPQ	22008
P43121	DQGEKYIDLRH	22046	O14525	PYNDYGDSKEI	22027	P32418	SLEAYCHIKGF	22008
P29218	QVIPLQRDDED	22046	P05023	PGGWVEKETYY	22026	P27986	LAYPVYAQQRR	22008
O15547	DTHLPTHSGSL	22046	Q13112	ENKGGTESLDP	22026	Q9Y3C3	AKEQIKWSLLR	22007
P19021	APLPALAPSSS	22045	P42898	PTQNARETEAP	22026	P08173	LCQYRNIGTAR	22006
P16233	REEVLLTLTPC	22045	O14646	TPEHTWSSRKT	22025	Q9Y243	PQFSYSASGRE	22006
Q9Y4Y8	VLYISTQKRRM	22045	Q92828	IKNLRMGSEQL	22025	P01884	QPKIVKWDRDM	22006
Q13496	SQMMPHVQTHF	22045	P22680	DIEFKYKFKHL	22025	Q02045	YVITHGEEKEE	22006
P55036	GKKDKKEEDKK	22044	Q16570	SSHLDTLGSKS	22025	P20594	LGERKGPPGLL	22005
Q9ULW5	REGRGASCCRP	22044	O00264	EPKDESARKND	22025	Q13329	LPGPLQDMFKK	22005
P53582	DSARPHFMSQF	22043	P35579	KADGAEAKPAE	22025	P49184	SLLSPQLCPAA	22005
Q99418	RISVKKKQEQP	22043	O00330	KANLENPIRLA	22025	O94772	SLGPALLWAGP	22005
P16671	SYCACRSKTIK	22043	Q9UNX4	KKREKLILTLT	22025	P55199	EYDQRQLQAWP	22004
P08253	FGSIKSDWLGC	22043	P48509	CLYRSLKLEHY	22024	P24530	NFRSSNKYSSS	22004
P06733	AGRNFRNPLAK	22042	P32321	SINSRPSQKLQ	22024	Q9Y6R4	HSFVKVCTDEE	22004
P14867	EPQLKAPTPHQ	22042	Q01726	RTLKEVLTCSW	22024	Q92982	QKPLMDMAPQQ	22004
P55085	SSSSTTVKTSY	22042	Q14982	GTLLAHFFIKF	22024	Q13520	PGSGAVEMESV	22003
P57723	KKAERQKFSPY	22042	P02775	QKKLAGDESAD	22024	P46092	PTETHSLSWDN	22003
Q99856	PSTSTSNNSLP	22041	P43699	SCSTLLYGRTW	22024	O43677	ILEYKRRNGLE	22003
Q13491	FKSREDCCTKF	22041	P35348	TISLSENGEEV	22023	P41159	MLWQLDLSPGC	22003

ID: ID from Uniprot Database (http://www.uniprot.org/) **Sequence**: Listed sequences match the Uniprot ID, but for the array experiments and for the computational predictions, the cysteines were changed to serine. **NA**: not available. **BLU**: biochemical light unit.

Table S1: Binding data from CAL peptide array.

ID	Sequence	BLU	ID	Sequence	BLU	ID	Sequence	BLU
P43115	STLMWSDHLER	22002	Q14129	QCDQKGSPVPP	21979	P04436	IFGSGTRLSIR	21960
P50583	EGHQFLCSIEA	22001	P12314	GVHRKEPQGAT	21979	P16860	SGLGCKVLRRH	21959
Q15319	KQKRMKYSAVH	22001	P36959	RVTQQHNTVFS	21979	P56177	FKKLMKQGGAA	21959
O00472	EFDQQQAESWS	22001	Q14814	KRMRLDTWTLK	21979	Q99500	NAALQNGIFCN	21959
Q93086	CPQLLEPHRST	22001	O95263	KCKSLRLPSDS	21978	O43248	RLQYFSGNPLL	21959
O15142	RVLEKLGVTVR	22000	P22888	ALLDKTRYTEC	21978	Q9UPM6	NRGEKVILFQY	21959
P15267	PLAGNPVSPTS	21999	P32780	TWQSRRLMKKT	21977	P01303	PRTRLEDPAMW	21959
P29400	ISRCQVCMKRT	21999	P20908	GFEVGPACFMG	21977	Q99462	CVRKSQETAFE	21959
Q14805	MPEDVLAERAL	21999	O75909	AAEPAPSQHLW	21977	Q16394	LRKKYRDIERL	21957
P14780	VTYDILQCPED	21999	Q13144	KEAEEESSEDD	21977	Q92902	RLWEASRIPLL	21957
Q14929	SGHHLLPQEVF	21999	P41134	CVPADDRILCR	21977	P23588	EDENEGEDYAE	21957
P42568	LQSYLETSGTS	21998	P34969	TTVEKKVMIHD	21976	P53779	EASAGPLGCCR	21957
P35219	LSDRVIRAAFQ	21998	Q9UQ03	LKNLRNSPKNC	21976	P01777	SAVWGQGTLVT	21956
P49716	FLPAAGTADCR	21998	Q13115	LHSPITTSPSC	21976	O95750	LEAVRSPSFEK	21955
O14949	EQRIRMTVPCL	21998	P21802	QYPHINGSVKT	21976	Q13304	NESSLSAKSEL	21955
P42024	DGARSIHRKTF	21997	Q02790	AGSQSQVETEA	21976	P23276	LLNPSSRCQLW	21955
P10911	MRPVSEMALLY	21996	P43034	VDQTVKVWECR	21976	Q99759	LLTHHFAQLMY	21955
P12524	LQKRIAYLSGY	21996	P33032	FRIACSFPRRD	21976	Q9UI95	LYVEERAHKGS	21955
P25112	LESEREARRLR	21996	P09237	LYGKRSNSRKK	21976	P32930	GAEEFQEMVHS	21955
P30419	MGAEKVGLVLQ	21995	O00217	AANIQADYLYR	21976	P00480	DYSPQLQKPKF	21955
P02656	PEVRPTSAVAA	21994	P49703	KKAARGGKKRR	21975	P16066	LLGERGSSTRG	21954
P12525	LQKRIEYLSSY	21994	Q03001	MTGISSLYYSS	21975	P78560	VDPSLLLHMLE	21954
P25208	QISGVQQIQFS	21993	O75956	CLAETERNART	21975	P22894	RGNKWLNCRYG	21954
O14548	ALYNASQPKNK	21993	P28472	FNLVYWLYYVN	21975	O00254	TRNHSTAYLTK	21954
O00214	GDIHLLEVRSW	21993	Q99551	EAKLKKLSRFA	21975	P48443	MEMLETPLQIT	21954
P10451	HELDSASSEVN	21993	P48454	HRSDQGKKAHS	21975	P29320	ETQSKNGPVPV	21953
O95782	RHLCELLAQQF	21992	Q9Y2Q0	YDTTKQRPDEW	21973	O15265	SSLLHQPKARP	21952
P54646	MCASLITTLAR	21992	P46055	GDDEFFDLDDY	21973	P27701	HSEDYSKVPKY	21952
P32320	SFGPEDLQKTQ	21992	Q13113	EEEGKVRSTPM	21973	O14944	VTSGDPELPQV	21951
P00156	SLIENKMLKWA	21992	Q00987	PIQMIVLTYFP	21973	O15360	DADLSQEPHLF	21951
P54289	WLVSGSTHRLL	21991	Q9ULV4	KLEQQMAKIAA	21972	P43365	LHEWAFREGEE	21951
P09093	IDWIEETIASH	21990	Q16626	LQARQASPAWK	21972	Q16281	SIGYSDLFCLS	21950
P35638	LIDRMVNLHQA	21990	Q14028	KMPEEREEKAE	21971	P35452	VVLREQALALY	21950
P24666	RCCRAFLEKAH	21990	P51674	TRSKERLNAYT	21971	Q14168	EPQWVPVSWVY	21950
P50579	CKEVVSRGDDY	21989	P04637	LMFKTEGPDSD	21971	Q01534	CGAIPCNTTRG	21950
P07498	TTTVAVTPPTA	21989	Q9Y295	EDEDVIQIVKK	21970	Q9Y2D0	KPKPATSQATP	21949
P16083	IPCTAHWHFGQ	21989	P23434	AYEKYIKSIEE	21970	Q15329	CDLFDVQILNY	21949
Q13474	SDVTANTLLAS	21989	O95168	GKLDRTFHLSY	21970	P15408	DSLNSPTLLAL	21949
Q9Y5R2	TYYKRPVQEWV	21989	O95050	CFIVARKKPGP	21970	P80192	PGGTGSSWGGQ	21949
Q15274	AKEVAPVPKIH	21989	Q15758	ATVASEKESVM	21969	O43808	TVMGLKRAHQH	21949
P54108	KASCNCSNSIY	21988	P29323	HKESNDCSCGG	21969	Q13077	MFLKCIVETST	21949
P51671	YLDQKSPTPKP	21988	P43268	QPFGPKGGYSY	21969	P01908	SVGASRHQGPL	21948
Q13233	LLKHPVFRTTW	21988	P14151	KKSKRSMNDPY	21969	O43261	YQMGDCCKEEI	21948
P43362	EEVLGEEQEGV	21988	P51608	DSRTPVTERVS	21969	P98170	VITFKQKIFMS	21947
O15350	IKEEFTEAEIH	21988	P04731	KGASEKCSCCA	21969	P48449	LYPERALAGHP	21947
P78314	LRHPYGYTGPR	21987	Q9Y4K1	TQVWEAMVLYT	21968	P12872	LSEMLPQHAAK	21947
O43747	EVNNFPPQSWQ	21987	P31371	PELYKDILSQS	21968	P00966	YHRLQSKVTAK	21946
O15178	ASWTPVSPPSM	21987	O75627	LALNIDCDLLG	21968	O00763	LLSTMDSPAST	21946
O43683	VLLLECKRSRK	21987	Q06413	KRMRLSEGWAT	21968	Q02318	KVGLQFLQRQC	21946
Q13163	LEERRSQQGPP	21987	O60287	CKDAASAASDA	21968	P40926	KKGEDFVKTLK	21946
P35658	SSVQGFGGWRS	21987	P17174	TSIHEAVTKIQ	21967	Q14849	RQRISELGARA	21946
Q99574	MNTSGHDFEEL	21987	Q9Y3D8	WIEQWIKDHNS	21967	P57056	SHGCESHPITF	21945
O15355	NSDKKKKAKRD	21987	P01040	DKNKDDELTGF	21967	Q92793	GDTLEKFVEGL	21945
P24534	QSMDVAAFNKI	21986	Q14409	LIGARYISGIP	21967	Q9UKT8	GSSFLAGEHPG	21945
P13727	CLRRLPFICSY	21986	P47929	GDVQLDSVRIF	21967	P11362	AQLANGGLKRR	21945
P38117	LVAKLKEIGRI	21986	P55001	AASCARSCGSC	21967	O60318	HLSALLDMVDI	21945
O00519	ERLMTPEKQSS	21986	Q02252	SPAVVMPTMGR	21967	P21673	KEYLLKMATEE	21944
O60858	VAEFVCKYKLL	21986	Q9Y2T4	IFQDKVNSDMH	21966	P51659	LQMILKDYAKL	21944
P33241	EKVLVEGGPAP	21986	O00115	MARKPSRAYKI	21966	Q13202	FSGSVEVIEVS	21944
O15525	TIVKSKTDARS	21986	P43361	IWEALSVMGAV	21966	P30793	TREEFLTLIRS	21944
P28039	IKQVFGDQGGH	21985	P10323	TTELPELTSTS	21965	P51888	MCFRLLQSVVI	21943
P41181	SPQSLPRGTKA	21985	Q03154	LASVPALPSDS	21965	P26437	LDWLSHELSKR	21942
P55061	MNEKDKKKEKK	21985	P78540	DESENQARVRI	21965	Q13370	IQVIEEADEEE	21942
Q16553	SLLPALLRFGP	21985	P98196	MLSQTSSSLSF	21965	P01031	EFAEDIFLNGC	21942
P31153	FPWEVPKKLKY	21985	P39748	TGAAGKFKRGK	21965	Q9UIV8	SILFFGRFSSP	21942
P29558	DHSPYTFQPNK	21985	Q12952	RRRKRKPKPGP	21965	P30990	PYILKRDSYYY	21942
Q16625	KMVGDYDRQKT	21985	O95968	TLGKIAEKCDR	21965	P49591	NRLQNMEVTDA	21942
P36955	FIGKILDPRGP	21985	Q15759	PKPPGSLEIEQ	21965	Q99972	MVTYDIKLSKM	21941
O75106	PDLPPFSYHGF	21984	P11217	RQRLPAPDEAI	21965	P09923	TLLLLGASAAP	21941
Q16568	TSCNSFLLKCL	21984	Q99965	EQPESESEPKG	21964	P54253	ICIEGRSNVGK	21940
Q92915	GGKPVNKSKTT	21984	Q13886	KRSKKALANAL	21964	Q15699	KEHTANISWAM	21940
Q9Y483	YLVEWEGATAS	21984	P11509	PRNYTMSFLPR	21964	P15311	TKQRIDEFEAL	21940
Q92569	PVHAQMPSLCR	21984	P06858	CHDKSLNKKSG	21964	Q93008	EEVSPPQTKDQ	21940
P15309	NSHQGTEDSTD	21984	Q13296	LIYDSSLCDLF	21964	Q13361	ENVDLQRPNGL	21940
Q16820	RPNLTLQNHAF	21983	Q02505	TSSMTTTETTS	21964	Q9Y6C7	DSELTSGLLAT	21940
P01036	MSLVNSRCQEA	21982	P80723	ANSDQTVTVKE	21963	P01889	SAQGSDVSLTA	21939
O60610	EEAKELVGRAS	21981	P49961	HKPSYFWKDMV	21963	P11171	VVHQETEIADE	21939
P04720	TKSAQKAQKAK	21981	Q92523	ADLFQVPKAYS	21962	P50052	SSLREMETFVS	21939
Q92636	DRQIIFWKLQY	21981	P08637	KFKWRKDPQDK	21962	Q02832	EVPGRRPLLHS	21939
P24844	ILKHGAKDKHD	21981	Q9UBU8	VAPPEYHRKAV	21962	O15520	SAHFLPMVVHS	21939
P11117	YRHVADGEDHA	21981	P41586	MSGLPADNLAT	21962	P07098	DIVSMISEDKK	21939
Q9UKJ3	QDLQHPPSHGT	21981	P49407	DGTGSPRLNDR	21961	O60361	YKSCAHDWVYE	21939
Q9UM01	EMPKQRDPKSN	21981	P14735	KPHINFMAAKL	21961	P51003	KRTSSPHKEES	21939
O15264	KDSRRRSGMKL	21980	P41236	SDQQQNKLRSS	21961	P23471	GNIAESLESLV	21939
Q99546	SGLPPSESGPW	21980	O00182	GGDIQLTHVQT	21961	P23141	EKPPQTEHIEL	21938
P23526	DGPFKPDHYRY	21980	P25963	DCVFGGQRLTL	21961	P29275	AGVQPALGVGL	21937
P40617	RKMLRQQKKKR	21979	P56559	RRKSLKQKKKR	21960	Q01432	MKSEEITALTN	21937
O76075	LKRKQPVRKRQ	21979	O95255	FYRLAQESGLV	21960	Q99217	STDKTKREEVD	21937

ID: ID from Uniprot Database (http://www.uniprot.org/) **Sequence**: Listed sequences match the Uniprot ID, but for the array experiments and for the computational predictions, the cysteines were changed to serine. **NA**: not available. **BLU**: biochemical light unit.

Table S1: Binding data from CAL peptide array.

ID	Sequence	BLU	ID	Sequence	BLU	ID	Sequence	BLU
Q9Y305	ATLRKDYLVEP	21937	O15218	PISPTQPLTPS	21914	O43921	TIPVLWTLLGS	21889
P26641	GKAFNQGKIFK	21937	P46736	EELMQELSSLE	21914	P56851	SIVAWTGMLIA	21889
P39060	CIENSFMTASK	21936	Q92185	PCEDTSLQPTS	21914	P14207	LALMLQLWLLG	21889
Q13437	LEKHGYKMETS	21936	Q16280	SLGYSDLFCLS	21914	Q02535	VISNDKRSFCH	21889
P43243	LAEERRQKKET	21936	Q13166	LAIMWFGTNTN	21914	P48730	ASSGLQSVVHR	21889
Q13635	CEERPRGSSSN	21936	P40145	QAEGTDKSDLP	21914	Q15784	MYEELNAFFHN	21889
P31947	EGGEAPQEPQS	21935	P07992	DVLHEPFLKVP	21914	P31946	GDEGDAGEGEN	21888
Q13412	CTSWYCKVKMS	21935	Q06828	LCLRLASLIEI	21914	O14791	NYKILQADQEL	21888
P35251	PRKGKGKSSKK	21935	Q9UI17	RLQKKGGKDKT	21914	P07384	LFKWLQLTMFA	21888
P56645	SFIIGRHKVRT	21935	Q9NYK5	QSKATEECTST	21914	P00403	KIFEMGPVFTL	21888
P28288	QITEDTVEFGS	21934	P05814	PLAPVHNPISV	21912	P30043	GHSTYPSHQYQ	21887
P00374	KYKFEVYEKND	21934	O75454	LFLEMLEAKVC	21912	Q13562	MSAQLNAIFHD	21887
P13378	AQELEEDRAEA	21934	P50152	PFKEKGSCVIS	21912	P28336	NGHSMKQEMAM	21887
P55344	VHECRRLSTPR	21934	P09238	HILKSNSWLHC	21912	Q15113	SQPVRAAASQD	21887
P56643	NTLSYFQSLIK	21934	P49238	HTSDGDALLLL	21911	Q9UFM2	PARLDGQGLAS	21886
O43614	AANGAGPLQNW	21934	P30279	STPTDVRDIDL	21911	P27797	EDVPGQAKDEL	21885
P56817	DDFADDISLLK	21933	P10588	STFNWPYGSGQ	21911	P43004	SVEEEPWKREK	21885
Q12837	KQKRMKYSAGI	21933	Q92837	RTGDGVLVPGS	21911	P22087	GVYRPPPKVKN	21885
P02675	SMKIRPFFPQQ	21933	Q13322	KLKHHCIRVAL	21911	P16144	STHMDQQFFQT	21885
P08195	EGLLLRFPYAA	21932	Q99732	QTPEIAAWSRA	21911	P10253	LMGEQFLVSWC	21885
O00192	DAKPQPVDSWV	21932	Q92882	AEDYLDDEDSD	21911	O14684	LQILWEAARHL	21885
Q14565	ITAGGIGDAKE	21932	Q16548	CEMLSLLKQYC	21910	P30499	GSDESLIASKA	21884
Q92914	PEASPSSPPAP	21932	O15394	IIQSKEDDSKA	21910	P13073	KWDYEKNEWKK	21884
O15117	DIADGCIYDND	21932	P12644	QEMVVEGCGCR	21909	O43602	LDDSDSLGDSM	21884
P36639	LDYTLREVDTV	21931	Q99595	SSPFGDYRQYQ	21909	P06899	TKAVTKYTSAK	21884
P36544	NFVEAVSKDFA	21931	Q16819	IIAILSQRPRK	21909	P10073	FSESTHLVQHW	21884
P18859	PKFEVIEKPQA	21931	P15907	ATLPGFRTIHC	21908	P98164	TANLVKEDSEV	21884
P43146	KQLNAITGSAF	21931	O43497	GLSSDPADLDP	21908	P16284	YSRTEGSLDGT	21884
P15941	NPAVAAASANL	21931	O43323	LLYRLAEELLG	21908	P42655	EALQDVEDENQ	21883
O75056	QKPDKQEEFYA	21931	P31277	RLQYFTGNPLF	21908	P31152	ALHQARGPAGQ	21883
Q07699	KENCTGVQVAE	21930	O00767	KRTGDGNYKSG	21907	Q04725	DKKATVYEVVY	21883
P01034	MTLSKSTCQDA	21930	Q15646	KKKGEALFPAS	21906	P11310	VAREHIDKYKN	21882
Q15910	YVGIEREMEIP	21930	P35250	LCQKTMAPVAS	21906	P17213	FLLFGADVVYK	21882
P21589	SLWAVIFVLYQ	21929	O43184	VPRSTHTAYIK	21906	P17538	IPWVQKILAAN	21882
O95447	SPTEGKRKIII	21929	O60755	VHGGEAARGPE	21906	Q14397	DPLEILEPDVQ	21882
Q05469	AGVDGGCGGRH	21929	O15403	EHRVHVQMEPV	21906	P81172	HRSKCGMCCKT	21882
Q00325	ESLKKKLGLTQ	21929	P20138	TSTEYSEVRTQ	21905	P01222	PQKSYLVGFSV	21882
P05186	ALALYPLSVLF	21929	O75461	QQSEELLEVSN	21905	P11475	KLFLEMLEAKV	21881
P01008	FMGRVANPCVK	21928	P13662	DMFRLALHNFG	21905	Q14642	HAHVHKCCVVQ	21881
P05160	STLSYQEPLRT	21928	Q06278	PGSYVPWNVPI	21904	O75556	YDSIWCNMKSN	21881
Q13002	DRRLPGKETMA	21928	P29972	DINSRVEMKPK	21904	P55058	STAPTPSTAAV	21881
O00158	SNPLLPDQKVC	21928	P55039	EHEDVIQIVKK	21904	P22695	NLGHTPFVDEL	21880
P17342	EDSIRSHFSVA	21927	P34741	YQKAPTKEFYA	21904	P10109	ARQSIDVGKTS	21879
P15085	TIMEHTLNHPY	21927	P41968	ILCGCNGMNLG	21903	Q99502	ALHHALELEYL	21879
P22794	GTEKLTNKQIG	21927	P15173	SVAFPDETMPN	21903	P28358	IRELTANLTFS	21879
O75896	HVDFPVILYEV	21927	Q14591	QRSDLVKHQRI	21903	P56597	LCHHPIVEEPY	21879
P27544	LRNGLVKDKRF	21927	P12236	LVLYDELKKVI	21902	P40937	QVTRDLIVAEA	21878
Q9UI54	KLVTLSNYLNK	21927	P10915	LYGVYCFRAYN	21902	P81408	SLNGGSRETGL	21878
O14658	VKKPGKKCTVF	21927	Q9Y3F4	CIFPSAPDVKA	21902	Q00013	SPQWVPVSWVY	21878
P49640	LDQREEVPLTR	21926	P28908	EDPLPTAASGK	21901	P31273	WKKENNKDKLP	21878
P48169	KDTMEKSESLM	21926	Q9UBA6	SWREALYGCHA	21901	Q12968	ESLDLGRSDGL	21878
Q15185	DSDDEKMPDLE	21926	P33981	SKTFEKKRGKK	21901	Q13951	LAVTGKKTTRP	21878
O75688	DAGTKMSGEKI	21926	P20172	IGRSGIYETRC	21900	P01011	FMSKVTNPKQA	21877
P01160	GLGCNSFRYRR	21925	P13804	VPEMTEILKKK	21900	Q14488	GLSVDRLVNGE	21877
P20290	NFDEASKNEAN	21925	O43763	AASVSGLASVV	21900	P43364	EDALREEGEGV	21877
P56747	GPSEYPTKNYV	21925	P20142	GNNRVGFATAA	21900	Q92832	CSVDFECLQNN	21877
P16930	AGKVLPALLPS	21925	P00695	DVRQYVQGCGV	21899	Q99471	ALGAAQATAKA	21877
Q99795	TGRESPDHLDQ	21924	O15496	FLCEPDSPKCD	21899	P00918	LKNRQIKASFK	21876
P18085	LDWLSNELSKR	21924	Q9UHM2	LGKFISTVHPI	21898	P07332	QELQSIRKRHR	21876
Q13495	GTEIRSYGNDP	21924	P30533	GRISRARHNEL	21898	P55198	DKGASANQEKG	21875
Q05639	KSAQKAQKAGK	21924	P42772	VAGYLRTATGD	21898	Q02080	SLQASSEKTQQ	21875
O00501	ATGDYDKKNYV	21923	Q16595	LSSLAYSGKDA	21898	O95677	ALHQALELEYL	21874
P20711	LAADVLRAERE	21923	P01760	QGTLVTVSSGS	21898	Q01469	VTCTRIYEKVE	21874
P12955	DKAFTPFSGPK	21923	Q02078	VKRMRMDAWVT	21898	Q13461	PGFASGLERYL	21874
O95219	WTNAKECFSKM	21923	P35542	RFRPDGLPKKY	21898	P54802	KYYPGWVAGSW	21873
P49748	GGVVTSNPLGF	21922	P55197	SPSMEIMQVRK	21897	Q29459	ETPEEKQTTIA	21873
Q08828	YLPSAAAGKEA	21922	P53680	LKQLLMLQSLE	21897	P17735	CAEGSQEECDK	21872
Q02978	NKAYKRLFLSG	21922	P41161	SSLPYAEGFAY	21897	P43490	QLNIELEAAHH	21872
P14060	RHKETLKSKTQ	21921	P03372	TGEAEGFPATV	21897	Q99715	PYNGQSYPGSG	21871
P20963	DALHMQALPPR	21920	O60925	IREMLMARRAQ	21896	P07358	SGPASETLDCS	21871
O95178	EELGIPPDDED	21920	Q12972	PGKKPTPSLLI	21896	Q01543	THVPSHLGSYY	21871
Q92696	ELLPSVSSVLT	21920	P56202	PDMKPRVSCPP	21895	Q14623	FHPLGMSGAGS	21871
P40933	VHIVQMFINTS	21919	Q01094	CDFGDLTPLDF	21895	P22234	QADKKIRECNL	21871
P21912	MATYKEKKASV	21918	P41212	LDEQIYQEDEC	21895	Q12888	HPKYKHDYVSH	21870
Q03468	EGIWKLKPEYC	21918	P78334	NVLYWLVCLNL	21895	P02590	DEFLEFMKGVE	21870
P08590	YEAFVKHIMSS	21918	P22760	NQYIEWLKENL	21894	P54107	KATCLCDTEIK	21869
Q14554	HERLGKKKEEL	21918	O14561	DYIADKKDVYE	21894	P06132	VHKHSRLLRQN	21869
O15534	PALPTAGNCTS	21918	P17509	SAEEEEEKQAE	21894	Q13422	ITRGEHRFHMS	21869
P48357	IMENKMCDLTV	21917	P15514	RQENGNVHAIA	21893	P14784	ELQGQDPTHLV	21869
Q06787	DGQQPLVNGVP	21916	P54764	MQQMHGRMVPV	21893	O15553	TICPVGGQGPD	21869
Q92859	LMKDLNAITTA	21916	Q00587	ADAEEDDEVKV	21893	P20023	VYSVDPYNPAS	21868
P07311	KLDYSDFQIVK	21915	P15803	YAYIYCLCAAV	21892	P47870	FNIVYWLYYVN	21868
P02652	FVELGTQPATQ	21915	P51948	DAFSGLFWQPS	21892	P10720	YKKIIKEHLES	21868
P29973	PGAESGPIDST	21915	P19875	IEKMLKNGKSN	21892	P17927	QTNEENSRVLP	21867
Q15828	SQLLKHNCVQM	21915	P14555	NKHCRGSTPRC	21892	Q99766	VLLIENADDLQ	21866
P28562	LQSPITTSPSC	21915	P40424	GPGSVHSDTSN	21892	Q92912	NGGKVVNQDST	21866
P15328	SLALMLLWLLS	21915	P49736	DLKRKMILQQF	21891	P04901	GSWDSFLKIWN	21866
Q12767	FETKLGMNSPF	21915	P56373	STDSGAFSIGH	21891	P49755	RRFFKAKKLIE	21866

ID: ID from Uniprot Database (http://www.uniprot.org/) **Sequence**: Listed sequences match the Uniprot ID, but for the array experiments and for the computational predictions, the cysteines were changed to serine. **NA**: not available. **BLU**: biochemical light unit.

Table S1: Binding data from CAL peptide array.

ID	Sequence	BLU	ID	Sequence	BLU	ID	Sequence	BLU
Q09428	SVFASFVRADK	21865	Q9UPW0	IQDDFDWDSIV	21837	P11388	YLEESDEDDLF	21812
P18849	KEKLEFILAAH	21865	P30500	GSDESLIACKA	21836	P50553	EQELLDFTNWF	21811
O75503	LPIRNKTLSGL	21865	P54619	ALVLTGGEKKP	21836	P35520	NFVAAQERDQK	21811
O15516	SLPDPSKVQPQ	21865	P46100	KNPGPSQGKSM	21836	P54198	CQEQLDILRDK	21811
O95741	CTLATTPSPSP	21865	Q13410	LIPTQPSQGAP	21836	P55318	GLYSRSLLNAS	21811
P25391	VFLHSCPGTES	21865	Q13066	TPEEGEKQSQC	21836	Q9UHI8	FIDFCTMAECS	21810
P54793	VSQPRGPNEKR	21864	Q13588	FPRSYVQPVHL	21836	Q13956	ELHELAQFGII	21810
P04839	GVHFIFNKENF	21864	P05015	TNLQKGLRRKD	21836	Q16539	PPPLDQEEMES	21810
P02750	GQTLLAVAKSQ	21863	P07237	DDDQKAVKDEL	21836	P22455	SSFPFGSGVQT	21809
Q15822	LFLPPFLAGMI	21863	P16455	ATSGSPPAGRN	21835	P02679	HLGGAKQAGDV	21809
P00395	HTFEEPVYMKS	21863	P01890	VQHEGLPKPLT	21834	P51993	YQTRGIAAWFT	21808
O43909	PHDKTKCFKFI	21863	P47710	NYEKNNVMLQW	21834	P19086	IQNNLKYIGLC	21808
P36969	LVIEKDLPHYF	21863	P41970	PVLLSSNSQKS	21834	Q02809	TRYIAVSFVDP	21808
P50550	VRAQAKKFAPS	21863	P53674	SFPVLATEPPK	21833	O75493	RLHVDGVPHGR	21807
Q99726	QCLRCQEPPQA	21863	P30101	PKKKKKAQEDL	21833	P25090	SPPAETELQAM	21807
O15055	RTPKKTKMDPP	21862	P54136	DTLGIKPGPRV	21833	P98160	QAGANTRPCPS	21807
O00168	SSIRRLSTRRR	21862	P12235	LVLYDEIKKYV	21832	P54826	SILLLLLGPLF	21806
Q15172	MHSILSNTSAE	21861	Q03828	PDQRDEAPLTR	21832	P07357	CPGRKVQTQAC	21805
P37268	VTEDYVQTGEH	21860	P56378	LKASAPAPGHH	21831	P04765	EEMPLNVADLI	21805
P05230	ILFLPLPVSSD	21860	P27540	PDLTMFPPFSE	21831	Q02575	YISYLNHVLDV	21804
P18858	EDSGSDPEDTY	21858	P13498	VNPIPVTDEVV	21831	Q92482	KLAHVKHKEQI	21803
P49916	CIRKRRLVAPC	21858	O43364	LTTIDLQHLNY	21831	O15551	LGTGYDRKDYV	21803
P35225	LKKLFREGRFN	21858	Q13232	WEDSAGHWLYE	21831	P02763	EKERKQEEGES	21802
Q14376	KQNPSGFGTQA	21857	Q13519	RRRTLHQNGNV	21831	P11802	SYLHKDEGNPE	21802
Q01113	SVLSKARSWTF	21857	P51955	QLKSRQILGMR	21830	P13584	HLKPLGPGSGK	21802
Q13131	RLYVGPEVDLW	21856	Q9Y2G5	SGHFHTVCLLV	21830	P11518	AKAKELATKLG	21802
P40616	EWLVETLKSRQ	21856	P43652	CFNEESPKIGN	21829	P20848	FIGKVVNPTQK	21801
P56179	HQDTMQRPQMM	21856	O15392	RRAIEQLAAMD	21829	P53611	QRVNVQPELVS	21801
P52803	LLFLLAMLLTL	21856	P48052	AIMEHVRDHPY	21829	P49763	CHLCGDAVPRR	21801
P14384	FLVSLLHIFFK	21855	Q99829	SAKDPAQAPQA	21828	Q01955	SRCQVCMKKRH	21800
P08243	TLTHYKSAVKA	21854	Q01151	GLVTPHKTELV	21827	Q16790	YRPAEVAETGA	21800
Q16828	NVYQVDSLQST	21854	P06734	RLPTPSAPLHS	21827	P06681	HLGDVLNFLPL	21799
Q02779	TVPLCGAHGSH	21854	P30740	SILFLGRFSSP	21827	P20930	RGGRQGYHHEQ	21799
P15531	YTSCAQNWIYE	21854	P32881	INLQKRLKSKE	21827	P14672	ELEYLGPDEND	21799
Q99406	IQQEKECVQTS	21854	O15479	EALKDEEKAGV	21827	Q16849	EVNAILKALPQ	21799
P06727	EQVQMLAPLES	21853	P22001	CVNIKKIFTDV	21826	Q04917	DQQDEEAGEGN	21798
Q06187	SNILDVMDEES	21853	P54756	KVQLVNGMVPL	21826	P04054	KNLDTKKYCQS	21798
Q99549	DENSGEFGIIL	21853	Q9UBL9	TPTDPKGLAQL	21826	P23470	SDPAESMESLV	21798
Q13438	EGTGDLDEFDF	21853	Q16781	AWTRLYAMNNI	21826	P54105	AGQFEDADVDH	21797
P43307	RAQKRSVGSDE	21853	Q16206	KFCGFEGLKLT	21825	Q99613	GYRQQQSQTAY	21797
P14927	ERKEREEWAKK	21853	Q03181	PLLQEIYKDMY	21825	O00746	DGGQHSSIHPA	21797
P54753	LQMNQTLPVQV	21852	O60268	LSDHIEQMATE	21825	P51805	EQIISLVSSDS	21797
Q14696	ENRAGNKREDL	21852	P13942	GVLLGPVCFMG	21824	Q01851	KQKRMKFSATY	21796
O60678	LNNSTQTYGLQ	21851	P49917	CELQEENQYLI	21824	P09769	AEPQYQPGDQT	21796
P19784	AVLSSGLTAAR	21851	P28324	PGPFSPDLQKT	21824	P01815	WGRGTPVTVSS	21796
Q92886	LHTTPCFIPYH	21851	P01215	CHCSTCYYHKS	21824	O14933	FTLRFGVDRPS	21796
O00591	NVFYWAYYMYF	21850	Q9NR22	ETSVSNDYKMR	21823	O60829	AKTKEAGDGQP	21795
Q9NR09	SSKELPSDFQL	21849	O60939	DGEGNPDDGAK	21823	P08107	SGSGPTIEEVD	21795
P02790	QNVTSLLGCTH	21849	Q9UBL6	PAGEASPGCTP	21823	Q15904	KGPTISLTQIV	21795
P02786	SGDVWDIDNEF	21849	P07900	DDDTSRMEEVD	21823	P17948	NSVVLYSTPPI	21795
P06307	RRSAEEYEYPS	21848	Q92913	GGKSMSHNEST	21822	Q9Y2Z9	LKEQIMAFASK	21794
P56880	NNSTHNLKDYV	21848	Q99571	YEQGLASELDQ	21822	O95622	AMIAKMNRQRT	21794
P52797	IAFFLMTFLAS	21848	P35462	FRKAFLKILSC	21821	P04632	IQEWLQLTMYS	21793
P51956	KRAGWQGLCDR	21848	P17568	GPGEVDPKVAL	21821	O43525	DSVWTPSNKPI	21793
P28330	ELIAREIVFDK	21847	P25101	TDRSSHKDSMN	21820	P14324	GLARKIYKRRK	21793
P10909	LQEYRKKHREE	21847	P00439	GILCSALQKIK	21820	P05111	PNLLTQHCACI	21793
P02511	EKPAVTAAPKK	21847	P18848	VRKARGKKRVP	21819	P55103	PDMVVEACGCS	21793
P06241	TEPQYQPGENL	21846	P78543	PSKNYVMAVSS	21819	P47712	FNKEFLSKPKA	21793
O43865	NGPFKPNYYRY	21846	Q07092	PPMKTMKGPFG	21819	P35238	PIKRGRSCILL	21793
P02741	GEVFTKPQLWP	21845	P03950	VHLDQSIFRRP	21818	P40938	FMEDGLEGMMF	21792
P52569	PFIFHEKTSEF	21845	P39059	IENSFMTDARK	21818	O43488	LVAHECPNYFR	21792
O95757	HTKSSGEMEVD	21845	P52943	YDRDPEGKVQP	21818	Q99075	EKVKLGMTNSH	21792
P39687	REPEDEGEDDD	21845	Q01844	EHRQERRDRPY	21818	P01779	FDVWGQGTKVS	21792
P07360	DQFHVLDEVRR	21844	P30307	QIALLVKDMSP	21818	Q03405	ARLWGGTLLWT	21790
P49585	AAYDISEDEED	21844	P46952	TQDPACKKPLG	21817	P05141	LVLYDEIKKYT	21789
P28067	IIYFRKPCSGD	21844	P00736	DWIKKEMEEED	21817	P51679	STMDHDLHDAL	21789
Q9Y689	LEWMMSRLKIR	21843	P01922	SVSTVLTSKYR	21817	P43026	EDMVVESCGCR	21789
Q15173	TPQVAASGGQS	21842	P01282	ESPDFPEELEK	21817	P02743	GYVIIKPLVWV	21789
P52298	RGGYGKLAQNQ	21842	Q99622	GPAAGGSVAAS	21816	P01814	WGKGTTVTVSS	21788
P10916	VHIITHGEEKD	21842	Q15084	VELDDLGKDEL	21816	P42785	RDFYDSAGKQH	21788
P53384	HQSKEENLISS	21842	P55317	GVYSRPVLNTS	21816	O00469	TRYIAVSFIDP	21788
Q13472	GHTRPFCPQNR	21842	P35527	YGGGSGKSSHS	21816	Q12947	SVCQDIKPCVM	21787
O43914	SDLNTQRPYYK	21842	Q11206	EMGAIKNLTSF	21815	Q92520	EMEGCIPQKQD	21787
P49789	AEAAALRVYFQ	21841	P55786	LLQRKASPPTV	21815	Q9NQP4	GSNINLEADES	21787
Q16512	FLDFDFVAGGC	21841	P10966	ARLRFMKQFYK	21814	P37287	GGENNEISETR	21787
P08123	FFVDIGPVCFK	21840	O00507	EEVSSPQMKDQ	21814	Q93091	KLVPVHLDSIL	21787
P42081	TSSCDKSDTCF	21840	P41440	LPSDGVQNVNQ	21814	Q00610	GQPQPGFGYSM	21786
Q92731	SKEGSQNPQSQ	21840	O95837	LQLNLREFNLV	21814	Q13085	RILSTMDSPST	21786
P27539	EDMVVDECGCR	21840	P31930	RIRSGMFWLRF	21814	Q92599	QPLRKDKDKKN	21786
P35558	ILALKQRISQM	21840	P27348	EECDAAEGAEN	21813	P50747	MLRNLILPKRR	21785
P07711	GIASAASYPTV	21839	Q9Y426	TMNGAPVEPCT	21813	Q9Y3C8	GVIQHKEKCNQ	21785
Q16829	NLFPLNTLEST	21839	P00915	PLKGRTVRASF	21813	Q12946	VTYQDIKPCVM	21785
O43247	ASVLSPSLISQ	21838	P46108	LDQQNPDEDFS	21813	Q16401	PVSTTAVEGAE	21785
P04541	EAQGEAAKSES	21838	Q9UN99	DDHDGGEGNNN	21812	P46098	LVMLWSIWQYA	21784
P04198	LLKKIEHARTC	21838	Q99933	RLQSTNFALAE	21812	P20941	EHTKIEEEDVE	21784
Q13033	AGADALAKVFV	21838	Q99626	TGGVLNPTVTQ	21812	P18440	VPKHGDRFFTI	21783
O95674	KGMLTSTTEDE	21837	O00716	KLPLVEDFMCS	21812	Q14259	LLRRQAQQAGK	21783
P54803	QFDNFLVEATR	21837	P12273	RFYTIEILKVE	21812	P53370	HTTPITSRVAR	21783

ID: ID from Uniprot Database (http://www.uniprot.org/) **Sequence**: Listed sequences match the Uniprot ID, but for the array experiments and for the computational predictions, the cysteines were changed to serine. **NA**: not available. **BLU**: biochemical light unit.

Table S1: Binding data from CAL peptide array.

ID	Sequence	BLU	ID	Sequence	BLU	ID	Sequence	BLU
P02489	REEKPTSAPSS	21782	Q02548	PPAAATAYDRH	21748	P51857	DHPEYPFHDEY	21720
P29312	DEAEAGEGGEN	21780	Q92620	APRRTPARFGL	21748	Q07001	YSYNVQDKRFI	21720
Q07817	VVLLGSLFSRK	21780	P32239	SYTTISTLGPG	21747	Q13609	TLRKKTKSKRS	21720
Q13895	KGFGRTPRPRW	21780	O15522	LASPALVSWNW	21747	O14975	YNAISAKTLKL	21720
Q12830	CVANPENLHWL	21780	O76031	GWPRQADAANS	21746	P40123	KLITEPAEIMA	21719
Q9UJU5	AAAAQAKWPAQ	21780	P01350	DFGRRSAEDEN	21746	P55211	FLRKKLFFKTS	21719
O75385	RLSALLTGICA	21780	P82094	QIDELLRQSLS	21746	O00292	RRLQHRPWCIH	21719
Q01433	EAGITMSPGPQ	21779	O43716	KLDEQEPFPHS	21745	O43715	GHGKEKPENSS	21718
O43684	DAETKPKSPCT	21778	P20749	PVPPSPAPGGS	21745	O60733	FQKLIQLLLSP	21718
P16422	EMGEMHRELNA	21778	O15540	DVVAVRHYEKA	21745	O14521	ICKAVAMLWKL	21717
P48546	NEASRELESYC	21778	Q12809	PLHRHGSDPGS	21745	O95833	LAAYRPAVHPR	21716
O60779	DPQSSSQVTTS	21778	Q13563	AGGNGSSNVHV	21745	P49247	GSVNMREKPFC	21716
P52565	WNLTIKKDWKD	21777	P12318	LPPNDHVNSNN	21744	P11474	LFLEMLEAMMD	21715
P55316	NQGSSSNPLIH	21776	P48146	FRKNFRSILRC	21744	Q13526	DSGIHIILRTE	21715
P01106	HKLEQLRNSCA	21776	Q15648	DDLMDVALIGN	21744	O08765	VAYSGENTFGF	21714
O76083	RDVKNSEGDCA	21775	P41226	DTAFPPLHYEL	21744	P42858	CLRNVHKVTTC	21714
P45954	NTIAKHIDAEY	21774	P25067	SSFSGFLLCPT	21743	Q02223	ATEIEKSISAR	21713
P09467	FLKVYEKHSAQ	21774	P30281	STPTDVTAIHL	21743	P51677	STAEPELSIVF	21713
Q92186	KLTVGQCDGAT	21773	O96017	TKRPAVCAAVL	21743	Q9Y5V3	NFGAIGFFWVE	21713
O15212	QAAKAGAPGKA	21773	P01778	SDVWGQGTLVS	21743	P11476	TPVKQSGGGCC	21713
P53814	RHELASRGKNV	21773	Q15102	GQGAPLLEPAP	21743	Q14999	CTATQSFSTFR	21713
Q15057	LNRFQQDSQKF	21773	P18206	LRWVRKTPWYQ	21743	P02810	PQGPPQGQSPQ	21712
Q92968	SIGKDGEKQDL	21772	Q00796	MLKCDPSDQNP	21742	O95807	FKFGRTEDLWQ	21711
P04628	CTHTRVLHECL	21771	P19429	GMEGRKKKFES	21742	P23197	SEDDDKKDDKN	21710
P35813	DTDSTSTDDMW	21770	P04818	PHPTIKMEMAV	21742	Q9NY72	NKENSAVPVEE	21709
P53350	SRSASNRLKAS	21770	Q16740	APAAEPVPAST	21741	Q03014	IEGDKSYFNAG	21709
P07108	KVEELKKKYGI	21769	P57058	FDMADGVKTQC	21741	P50402	HFMQAEEGNPF	21707
P02771	LISKTRAALGV	21769	P13797	ACLMGRGMKRV	21741	P21217	QTVRSIAAWFT	21707
P31689	HPRGGVQCQTS	21769	Q9Y6A2	GWQPAPPPPPC	21740	P51808	IVNVFAIAIVL	21707
Q13241	KNRYICKQQLI	21768	P01781	WGQGTLVTVST	21740	P50395	KRKKNDIYGED	21706
P10124	GQHGLEEDFML	21768	P35408	SETLNLSEKCI	21740	Q99678	LTQALANGPEA	21706
O75843	EVNNLPVESWQ	21767	P30086	VPKLYEQLSGK	21740	Q00973	FKHRLQCMTSQ	21705
O00141	FSYAPPTDSFL	21767	P53609	KQCSENVHIST	21740	P53675	APLVFDFDGHE	21705
P30273	YETLKHEKPPQ	21766	P39877	QYQYFPNILCS	21739	P05413	AVCTRTYEKEA	21705
Q9Y512	GVQFGAGIRFL	21765	P57086	IRRRTDVRITG	21739	O95257	NDWVPSITLPE	21705
Q9Y261	GVYSRPIMNSS	21765	Q04941	AAPTDPADGPV	21738	Q02643	RSAAKVLTSMC	21705
Q07869	PLLQEIYRDMY	21765	P19237	MFDAAKSPTSQ	21738	Q92959	EYNVQKAAGLI	21705
Q14738	EEFLTASQEAL	21764	Q99798	SALNRMKELQQ	21737	P56706	QYTKVWQCNCK	21705
P05089	HKPIDYLNPPK	21764	P34947	NHVSSNSTGSS	21737	P09496	LISLKQAPLVH	21703
O60840	LGDEMACVHAL	21764	P01253	TIEQEKQAGES	21737	P49366	MDAFMHEKNED	21703
P24462	AESRDETVSGA	21764	P11245	VPKPGDGSLTI	21736	Q9Y5M6	LSWYSFILLVL	21703
P15172	GANPNPIYQVL	21764	Q14201	HWINPHMLAPH	21736	P49761	RSFHTSRNPSR	21702
P36873	PRGMITKQAKK	21764	O00230	NFFWKTFSSCK	21736	P00390	PTSSEELVTLR	21702
P28289	GPIIPKCRSGV	21764	P31941	RLRAILQNQGN	21736	Q05901	PALKMWLHSYH	21701
P02647	LEEYTKKLNTQ	21763	P22061	TDKEKQWSRWK	21736	P14635	VQDLAKAVAKV	21701
P12272	TTSLELDSRRH	21763	P32322	LPRSLAPAGKD	21736	Q14534	LIYSEMKYMVH	21701
P25774	GIASFPSYPEI	21762	P06744	FIKQQREARVQ	21735	P01009	FMGKVVNPTQK	21700
P12104	GVEAKRIFKKD	21762	P02585	DEFLKMMEGVQ	21733	P49450	RRIRGLEEGLG	21700
P39030	RRNTLQLHRYR	21762	P51911	NHAHNYYNSA	21732	P48507	GYILQAKRRGS	21700
O43295	PNSSADKSGTM	21762	Q12933	FIKAIVDLTGL	21732	P21128	YIATAYIVSST	21700
P28676	YDDFLQGTMAI	21761	O14530	GKKYDSDSDDD	21731	Q9UMN6	CGAKRCRRFLN	21700
P55884	TEEIIPLGNQE	21761	Q06210	PRNLAKSVTVE	21731	Q93050	FEHIREGKFEE	21700
Q99956	TSDGAFELAPT	21760	P15170	GKVLKLVPEKD	21731	O15247	KEIENTYANVA	21699
Q14139	AERKQQKEQLE	21759	P01775	FAHWGQGTLVT	21731	Q08493	PGDLPLDNQRT	21699
P16452	SVTVVAPELSA	21758	P20742	PCSTDTEHGNV	21731	P04469	EYDSLYPEDDL	21699
P14550	GHPLYPFNDPY	21758	P55017	NQENVLTFYCQ	21731	P02708	AGRLIELNQQG	21698
O15528	ERSINLQFLDR	21758	P31607	SKNYNMMTVSG	21730	P32238	YSHMSASVPPQ	21698
P19419	PVVLSPGPQKP	21758	P20815	VDSRDGTLSGE	21730	P24468	SSFNWPYMAIQ	21698
P08238	DEDASRMEEVD	21758	O15371	EEEEEEEEEET	21730	P20071	LVFDVELLKLE	21698
P22459	CSNAKAVETDV	21757	P54922	LGSKEDTVISL	21729	Q93009	TYLEKAIKIHN	21698
P02671	VRMKIRPLVTQ	21757	P45880	FFPPEDDQNKG	21729	O43931	LARKLAVLTPG	21697
O95749	HLSKMFKEENE	21757	Q99439	YPPYYQEEAGY	21728	P15516	RGYRSNYLYDN	21697
Q15506	SLQNEEKEENK	21757	P01833	AAEAQDGPQEA	21728	Q9Y2J8	PFTFKWWHMVP	21697
P01210	MEKRYGGFMRF	21756	Q92839	RRRTGGYRVQV	21727	P21817	CFRKQYEDQLS	21697
P51787	TVPRRGPDEGS	21755	P50336	AVSVLGTEPNS	21727	P22105	PRNFRSPAGGG	21697
Q15393	KLEDIRTRYAF	21755	P07714	CLYSRLTICEF	21727	Q12766	GEEEEVAAVAE	21697
Q15847	SGIGKKFGLLK	21754	Q05940	PIGEDEESESD	21726	P20036	SGHDPRAQGTL	21696
P08572	ISRCQVCMKNL	21754	P15882	ELLIKNEDILF	21725	P01776	FDVFGQGTLVT	21696
Q9Y375	NSPELNPRLFK	21754	Q01362	DPGEMSPPIDL	21725	Q99697	SACQYAVDRPV	21696
P32019	EFIHQFLCNPL	21754	P98161	RAKNKVHPSST	21725	Q9UQ74	VTGHFTFTLYR	21696
Q9ULW8	PFSFKWWNMVP	21754	Q16513	FRDFDYIADWC	21725	P98177	EGLDFNFEPDP	21695
P49356	KDETSAEPATD	21754	P30566	SVMKVKAELCL	21725	O76074	INGESGQAKRN	21695
P52757	QILIENEDVLF	21753	Q14156	YEMKFPDLCVY	21725	P18505	FNVVYWLYYVH	21695
P53804	QNQELPSCSSR	21753	O43325	PVYLRSHDEVS	21725	P10263	RKEILRHGNSQ	21695
P29372	VDRVAEQDTQA	21752	P23760	GQYGQSKPWTF	21724	P50135	NNTLSFIVIEA	21695
Q13740	KLEENNHKTEA	21752	Q9UJU3	NLHRNEDSVLF	21724	P27708	MALLATVLGRF	21695
Q9UGM5	AQNASPLVLPP	21752	Q16181	LEKNKKKGKIF	21723	Q07011	FPEEEEGGCEL	21694
P40967	ENSPLLSGQQV	21752	O14627	PIEIQQVIVSE	21723	O15217	YVRTVYNIFRP	21694
P17676	PEPLLASSGHC	21751	P32455	KMRRRKACTIS	21723	O95139	PPMKEFPDQHH	21694
P20694	LARECTIEATA	21751	O75388	SSCPRGNAPRE	21723	O14625	LIIKKVERKNF	21694
P11511	TPRNSDRCLEH	21751	Q15116	RPEDGHCSWPL	21723	P30260	TQLHAAESDEF	21693
O60575	QDIQIMKDGKC	21751	Q07326	WNRKQLTYKNN	21723	P01568	KIFQERLRRKE	21693
P07585	VRSAIQLGNYK	21751	O15428	HPEDQGRREGL	21723	P28715	LRRARGRKRKT	21693
O75506	SENKIPATQKS	21750	Q92733	SRRQTQAKYGF	21723	Q9NYP9	ESKLSFATCKS	21692
P05546	FMGRVANPSRS	21750	P30041	GKKYLRYTPQP	21722	P31150	KQNDVFGEAEQ	21692
Q16537	RGLRRDGIIPT	21749	O95817	MTDTPGNPAAP	21722	P24606	IVITKGHFAMV	21692
O60548	SKVAGLSGCHF	21749	P43234	GIADSVSSIFV	21722	Q06546	ATASLQTEKDN	21691
O14810	LPGPLQDMLKK	21748	P23945	ILVPLSHLAQN	21721	O60240	RTHYSQLRKKS	21691

ID: ID from Uniprot Database (http://www.uniprot.org/) **Sequence**: Listed sequences match the Uniprot ID, but for the array experiments and for the computational predictions, the cysteines were changed to serine. **NA**: not available. **BLU**: biochemical light unit.

Table S1: Binding data from CAL peptide array.

ID	Sequence	BLU	ID	Sequence	BLU	ID	Sequence	BLU
Q9Y5W5	RRDPPESNYIW	21691	P57738	AANEPLKTHRE	21666	P07477	KWIKNTIAANS	21642
O75131	PKNPATKQQKQ	21690	O75631	RAEVYSSKLQD	21666	P29597	QGQAPSVFSVC	21642
P24855	SDHYPVEVMLK	21690	O43903	SASYKAKKEIK	21665	Q9Y3D9	EVVTFPGNPNT	21641
Q92806	CLPPPESESKV	21690	P32004	TSPINPAVALE	21664	O95340	LTDYYRSLEKN	21641
Q15628	LGLTDPNGGLA	21690	Q13946	SQLLPQENRLS	21664	P53025	KSTMGKPQRLY	21641
Q13061	ANSPGQKQQGQ	21690	Q14469	TADSMWRPWRN	21664	P04895	QRMHLRQYELL	21640
P27469	GRALSNRQHAS	21689	O15072	RRPTRSSTLER	21663	Q06323	KPRGETKGMIY	21640
Q15643	GLGPGGPGIFF	21689	Q02742	LRHKALETLKH	21663	P08919	ELYLQSLTAEH	21640
P55145	APKAASAPTDL	21688	P28827	YEVALEYLNSG	21663	O75348	PEIHENYRING	21640
P19526	DLSPLWTLAKP	21688	P20333	LGVPDAGMKPS	21663	P04438	WGQGTWSPSLQ	21639
Q02325	IRMRDAVLFEK	21688	P15374	LRFNAIALSAA	21663	P31268	DDDEEEEDEEE	21639
P08134	KNKRRRGCPIL	21688	Q9UHT9	AIRTGAAIFTQ	21662	O14746	ALPSDFKTILD	21639
O14604	TIEQERQAGES	21688	Q14161	MQKKLLGKDAN	21662	P20851	LKKAELKAKLL	21638
O14757	VSSQKVWLPAT	21686	Q9Y5M8	EVSPHVKTGTK	21661	Q99447	AQPLGERDGDF	21638
O43826	TKMGRVSKKAE	21686	Q14289	VLANLAHPPAE	21661	O75962	NFLQSRLLPRV	21638
P17152	CVKKIYELYAV	21686	P22083	SIRNLASWFER	21661	P21281	SEFYPRDSAKH	21638
P42323	IVLLLQCDIQK	21686	P51570	QAADGAKVLCL	21661	P38398	YLIPQIPHSHY	21637
P51811	QFLNAEDLCSA	21686	P49915	TSKPPGTTEWE	21661	O00762	TYSKQVTSQEP	21637
Q15011	LLPEGPPAIAN	21685	O75031	LEDLRTLEHNV	21661	Q14011	DSYDSYATHNE	21636
Q9UKR8	YVAQAGLELLA	21685	P04792	AQLGGRSCKIR	21661	O00295	QAFSICLSSFN	21636
P37231	PLLQEIYKDLY	21684	O43252	LTEYYKSLEKA	21661	O00526	FIIALALGSRK	21636
P01225	PSYCSFGEMKE	21683	Q9Y3C0	ESSDSESSFSD	21660	P05067	PTYKFFEQMQN	21635
Q14106	SQQFQPVVLAN	21683	O00116	PNNIFGNRNLL	21660	P13671	MEILHPGKCLA	21635
Q13510	LRDCPDPCIGW	21682	O60383	EDMIATKCTCR	21660	Q11130	VYEDLEGWFQA	21635
P46093	QVQLKMLPPAQ	21682	P02304	RQGRTLYGFGG	21660	P21796	HKLGLGLEFQA	21635
O75791	FPANYVAPMTR	21682	P11086	VFFAWAQKVGL	21660	P11387	AIDMADEDYEF	21635
P35568	ISFQKQPEDRQ	21682	P38435	ESNPDPVHSEF	21660	P30039	PIFIADAFNVR	21635
O94903	VKAPLEVAQEH	21682	Q92974	RRSSPQAMPCT	21659	O00755	CSERTEMYTCK	21635
P24410	NKPKVQCCQNI	21682	Q13522	PPLDSKGANSV	21659	P18507	NLVYWVSYLYL	21634
P53365	GAEKPSWLEEQ	21681	Q99814	GDLLRALDQAT	21659	O14843	GTGGQVACAES	21634
P15121	SHKDYPFHEEF	21680	P06737	LSNESNKVNGN	21659	P22064	ALNLEKDSDLE	21634
Q92935	QRKKYRSLEKP	21680	Q10469	HELCKSYRRLQ	21658	O94759	QKAAAEFGAHY	21634
Q99928	NLVYWVGYLYL	21680	P13224	LVAERAGTDES	21658	P49754	GPGSAILEMKK	21634
P07902	GQKDRETATIA	21680	Q9UBM1	RQKASGSHKRS	21658	P29354	RNYVTPVNRNV	21633
P29034	NDFFQGCPDRP	21680	P16070	NLQNVDMKIGV	21657	Q13867	PAWDPMGALAE	21632
Q04724	DKKATVYEVIY	21680	P16383	LDHLKSLIKED	21657	P31644	EPVIKGAASPK	21632
P00750	YLDWIRDNMRP	21680	P26678	LLLICIIVMLL	21657	P04898	IKNNLKDCGLF	21632
O75648	PEDGPGLSPLL	21680	P08918	ELYLQHLTALH	21657	P13051	SGKKPIDWKEL	21632
P20827	LLLPLLLLQTP	21679	P34932	SDKKLPEMDID	21656	Q15050	GGGQRPGGKRRK	21632
P25686	DSLWEEKRGVS	21679	P48739	GSVRGTSAADV	21656	P02751	DVQADREDSRE	21631
P07226	LDQTLNELNCI	21679	Q9Y458	FAKGFRDTGRN	21656	P78367	LSTCAAAAGTQ	21631
P08670	NETSQHHDDLE	21679	Q14657	EWQDPGRPLES	21655	Q9UHV9	AKASSAGVLVS	21630
P04217	SDPVELLVAES	21678	P42765	QGIAVIIQSTA	21655	Q15036	FAFEGIGDEDL	21630
P18846	TLKDLYSNKSV	21678	O43570	PATKMETEAHA	21654	O00257	LTVTFKEYVTV	21629
Q12934	SDKKKSGEKSS	21678	P35575	QVLGQPHKKSL	21654	O43316	KATPTHFSHWP	21629
P52789	VACRIREAGQR	21678	P28069	FSISKEHLECR	21654	Q14694	YLLYYRRVDLL	21629
P13645	VGESSSKGPRY	21678	P25940	EGEGGAGGEPG	21653	Q9UPY5	ILEVVPEEDKL	21629
P36941	RGPRNQFITHD	21678	O60658	MKLRNLRPPPE	21653	O95479	VWYMDYDAFLG	21628
Q13595	SRSRSYSPRRY	21678	Q10981	IAADLSPLLKH	21653	P48061	LEKALNKRFKM	21628
P31944	QSTLRKRLYLQ	21677	P28072	PKFAVATLPPA	21653	P23771	HHPSSMVTAMG	21627
P15863	LHGLPIPASTS	21677	Q07157	ANCVSVLIDHF	21652	P17275	QLLLGVKGHAF	21627
Q16647	HDVPVRYRIRP	21677	P35637	EHRQDRRERPY	21651	Q9UBV4	CESMTDVHTCK	21627
P17900	CIKIAASLKGI	21677	Q01415	PGGGALVLLEA	21651	P49638	DYLSSISESIQ	21625
P11230	TYHLPPPDPFP	21676	O94782	STPYLLFYKKL	21651	Q9UL15	YLDLKSDEWEY	21624
P53367	GVDAPSWLEEQ	21676	P23582	DRIGSMSGLGC	21650	P01861	TQKSLSLSLGK	21624
P25685	SRTVLEQVLPI	21676	Q9Y2I7	PDHWTGLGLNC	21650	P14136	KESKQEHKDVM	21624
O60493	KSYTPSKIRHA	21676	P48145	LRQLITCRAAA	21650	P09210	SLEESRKIFRF	21624
P41439	AGAPSRGIIDS	21675	P52945	SVAPRRPQEPR	21650	P10163	PRPAQGQQPPQ	21624
P08151	PGETEFLNSSA	21674	Q00978	PEQQAAILSLV	21650	P01585	TFCQSIISTLT	21623
P12079	ILGYTRRATSW	21674	P50607	LSSFDSKLACE	21650	Q9Y5X3	LQSCIDLFKNN	21623
P26367	PDMSQYWPRLQ	21674	P41002	EEDMNLGLVRL	21649	Q04323	AVLIVAKKCPS	21623
P42694	SGSNGFYSYFK	21674	O14626	QKEKLRCENNA	21649	Q12948	SGAFVYDCSKF	21622
P49902	DDDEEEEEEEE	21673	O95985	DKMSALAAYFV	21649	O95390	PGMVVDRCGCS	21622
P09758	ELGELRKEPSL	21673	P31940	PTEVQEPIITA	21649	P05014	TNLQKRLRRKD	21622
P52952	GVSTLHGIRAW	21673	Q9Y696	AYSDVAKRLTK	21648	P98077	HLLLVDPEGVV	21622
Q99614	INFVQNPNNNR	21673	P49289	TAGAIMETNLK	21648	P51854	IIVAVKCMLLN	21622
P41597	PEASLQDKEGA	21672	O95816	TLQQNAESRFN	21647	O60232	EALRSLQQLQH	21621
P50238	GRGGAESHTFK	21672	P20718	LPWIKRTMKRL	21647	P52566	WNLSIKKEWTE	21621
Q99819	WGLCICQDWKD	21671	P41221	CTEIVDQFVCK	21647	P13472	TIEQEKRSEIS	21621
P06730	SGSTTKNRFVV	21671	Q92947	ITGIQAFTASK	21646	O14813	KPGPALKTNLF	21620
O60637	YELLITGGTYA	21671	Q14410	LIGARYISGVP	21646	P07996	FSDLKYECRDP	21620
P43235	GIANLASFPKM	21670	Q15127	GALHVYSFGSD	21646	O14514	GQDIIDLQTEV	21619
P98174	PPESPQTRDKT	21670	P48059	YEISIGAEEKT	21646	Q16589	FKVAQTLCFPS	21619
P22301	IEAYMTMKIRN	21670	Q99705	ADEERTESKGT	21645	O15315	EGLVLQAYGNS	21619
Q9Y2G7	LTYHQRIHNVT	21670	P14616	DCSPQNGGPGH	21645	Q9Y5P3	KQRLATFFPRK	21619
P33897	QGPGGLQGAST	21669	Q15765	RNKDDSTKNKA	21645	P49792	RRITITECGQI	21619
P47902	SPMPVKEEFLP	21669	O14681	SPAKLKATAGH	21645	P00734	WIQKVIDQFGE	21619
O43189	YLVEWGGGGIF	21669	P15154	VKKRKRKCLLL	21645	Q9Y6I4	EHQAKAGSDKL	21619
Q9Y6C5	LSSRGPGPATG	21669	Q92737	GALHPARCSLM	21645	P31933	GGQGEALLNTT	21619
Q06418	LQQGLLPHSSC	21669	P13521	EHIAKRAMENM	21645	P41238	TGLIHPSVAWR	21618
O60830	APGYPSYQQYH	21668	O95411	HKLNLRGKAWG	21645	P09972	AQSLYIANHAY	21618
P81277	LEGGAMSSQDG	21668	Q9Y5B6	IWTDRPCVVFS	21644	P09497	LMSLKQTPLSR	21618
Q92609	SGFTIVSPLDI	21668	P17535	QLLPQHQVPAY	21644	Q07343	IATEDKSPVDT	21618
P51636	FSSVSLQLSQD	21667	P31639	AVAVFLWGFYA	21644	Q08499	ACVIDDRSPDT	21618
P01024	TESMVVFGCPN	21666	Q93068	YQEQTGGHSTV	21644	P32298	PSEKEVEPKQC	21618
P52849	LREELQHSSLG	21666	P23025	MCGHELTYEKM	21643	P14210	KIILTYKVPQS	21618
O00233	LLGCNIIPLQR	21666	P19447	HVHPLFKRFRK	21643	Q15907	KPNKLQCCQNL	21618
Q9UQ72	GVTGYFTFTLY	21666	P35453	KIVSKLKDTVS	21642	P23497	PPSTRKKVTIK	21618

ID: ID from Uniprot Database (http://www.uniprot.org/) **Sequence**: Listed sequences match the Uniprot ID, but for the array experiments and for the computational predictions, the cysteines were changed to serine. **NA**: not available. **BLU**: biochemical light unit.

Table S1: Binding data from CAL peptide array.

ID	Sequence	BLU	ID	Sequence	BLU	ID	Sequence	BLU
P18847	IQQIKEGTLQS	21617	P27924	VETATELLLSN	21597	P43487	EETKEDAEEKQ	21575
P49759	HPFFDLLKKSI	21617	O95972	EGMIAESCTCR	21596	O60704	QNSTSSHLGSS	21575
O14793	PAMVVDRCGCS	21617	Q99853	PASALHSVAVH	21596	O76071	AFWKYQRPEGL	21574
O00270	PPDFNPRDSYS	21617	O60628	VSDIEAVRASW	21596	P50213	EEICRRVKDLD	21574
Q9Y277	HKVGLGFELEA	21617	P12544	NWIIMTIKGAV	21595	P08476	QNMIVEECGCS	21574
P30047	GQTLVWCLHKE	21616	P18084	KFNKSYNGTVD	21595	P53041	YANTLLQLGMM	21574
Q02083	QANLSLEALFQ	21616	Q9Y487	SSKFNNDDSVA	21595	P20472	VDEFSTLVAES	21574
Q09470	CVNKSKLLTDV	21615	P10070	AEESKFLNMMT	21594	P01100	DSLSSPTLLAL	21573
Q00169	KDPVKGMTADD	21615	O75364	SPCQYAVERPV	21594	P16402	TKAKKAAPKKK	21573
P49760	KLWDSSRDISR	21614	Q13103	RHRARINTDFE	21593	P28062	DLLHQYREANQ	21573
O14610	PFKEKGGCLIS	21614	Q12774	TAKLQLVEQQA	21593	P17787	LHSDHSAPSSK	21572
P50148	LQLNLKEYNAV	21614	P05108	FWPFNQEATQQ	21592	P01574	FINRLTGYLRN	21572
P39905	RKHSAKRCGCI	21614	P40818	SLGPRVTDVAT	21592	P00790	ANNQVGLAPVA	21572
P09681	DQTNLCRLRSR	21614	P14314	EAPTEDDHDEL	21591	Q9Y5K1	LPNKLKFGGWI	21572
P50552	FVQELRKRGSP	21614	Q99680	EKRLVPQVVTD	21591	P56554	AKQIVQKSLGL	21572
P30556	TKKPAPCFEVE	21613	P50452	CILFCGRFSSP	21591	Q93034	ESDINTFIYMA	21572
O95832	KPAPSSGKDYV	21613	P13805	AGKGRVGGRWK	21591	Q93098	GGAAHKPGRKP	21572
Q15155	RQAKKQKTRRT	21613	Q16082	EEEEEAAIVEP	21590	P36888	LLSPQAQVEDS	21571
P18124	DQINRLIRRMN	21613	P01742	EYNGGLVTVSS	21590	P56159	LSTLLSLTETS	21571
Q13829	RQLGHQSTHRD	21613	O95825	NQGRKKQVVQF	21590	P40197	FRKLIRERALG	21571
Q9UK80	YQLMQEPPRCL	21613	P29401	AQAVRGLITKA	21590	Q03013	LYTRVAVWGNK	21571
P41223	CTHCGCRGCSG	21612	O95858	AGTGCCLCYPN	21590	P29043	RLKGDKMRDEL	21571
P09630	REETEEEKQKE	21612	P49459	SAIVEQSWRDC	21590	O43416	FLKQESFPTSE	21571
P51149	RAKASAESCSC	21612	P09544	APKNADWTTAT	21590	P01744	WGQGTTVTVSS	21571
P12319	NPHPKPNPKNN	21611	O60269	SCCGCSGAAPE	21590	O43511	VQDEAMRTLAS	21571
Q9UBS5	CDGSRVHLLYK	21611	Q03113	LQENLKDIMLQ	21589	O60861	TGNIRPVDMEI	21570
P16871	YVTMSSFYQNQ	21611	P02538	TSSSSRKSYKH	21589	O75459	TPEEDEGQSQP	21570
P13232	WNKILMGTKEH	21611	P28070	WDIAHMISGFE	21589	P30550	INGNICHERYV	21570
Q9Y2B9	QPQSSDGTTSS	21611	P50914	APAPKASGKKA	21589	P33551	FRRPLPKKPKK	21569
P81489	PQEGNKSRGAR	21611	P09494	LDQTLLELNNM	21589	P13639	IPALDNFLDKL	21569
Q02040	SPSRHRSTWNR	21611	O00418	KAEEAWAQMEE	21588	P29777	IAKNLRGCGLY	21569
P49190	EGCQGETEDVL	21610	P49795	LQGPSQSSSEA	21588	P48637	GVAVLDNPYPV	21569
P15336	APSSQSQPSGS	21609	P30968	FDPLIYGYFSL	21588	Q15390	MKALIENVSDS	21569
P51681	STGEQEISVGL	21609	Q14240	EEMPMNVADLI	21588	O96001	DDEKDGDKIAI	21568
Q07352	PIFSRLSISDD	21609	Q03527	KQEGTPEGLYL	21588	P49773	LGGRQMHWPPG	21568
P48995	TSKYAMFYPRN	21609	Q16881	RSGASILQAGC	21588	P30536	HGWHGGRRLPE	21568
P10746	IRKALQPHGCC	21607	Q16831	SYFIKKKLSKA	21588	Q13200	VILRKNPNYDL	21568
P25092	LNTTDKESTYF	21607	Q12765	DCVDTEIKFFK	21588	Q9UHP3	LSLSRTPADGR	21568
P80108	GALHVYSLGSD	21607	Q13286	LPLHDFLCQLS	21587	Q14576	VSFKTSKQHKA	21567
P05187	TLLLLETATAP	21607	O60636	CCAIRNSRDVI	21587	O15063	GERRPAYLPQY	21567
P53794	LGIFMFVYFSL	21607	P08754	IKNNLKECGLY	21586	P01743	SEDTAVYYCAR	21566
P56847	PGLCQHKVGAR	21607	O00178	QGACVTPASGC	21586	Q14669	AREGQQSFHLS	21566
Q9UGP9	DKTIKLWKSDC	21607	P24386	TNLGNLEESSE	21586	P03973	CGKSCVSPVKA	21565
Q9UMQ6	LEQWLQMTMWG	21606	P30542	DEDLPEERPDD	21585	P49715	SSLVKAMGNCA	21565
Q13439	LMFTSPRSGIF	21606	P01903	KSNAAERRGPL	21585	P06276	YTSKKESCVGL	21565
P01906	SVGASRHQGLL	21606	P37140	PPRTANPPKKR	21585	Q64252	PNWATQDSGFY	21565
Q13087	ANSTMGSKEEL	21606	P35237	GILFCGRFSSP	21585	O75293	QWVPYISLQER	21564
Q99453	AKAALVKSSMF	21606	Q02543	RFTTKRPNTFF	21585	O14893	FDQRDLADEPS	21564
P22891	RYSLWFKQIMN	21606	P07478	DWIKDTIAANS	21585	P48544	LGGSREARGSV	21563
Q92995	GYMYFYRRIPS	21606	Q01167	PAAVREKGVQN	21584	Q13882	LSSFTSYENPT	21563
O95847	KIREMSGVSPF	21606	Q04671	LLVAHVVVGWN	21584	P31431	YKKAPTNEFYA	21563
Q93073	HSDSNYTTQTT	21606	Q04844	PDLPYAPCIQP	21583	P36897	SQLSQQEGIKM	21563
Q9Y2Z2	CDADRLQEREL	21606	P78537	VYKGQLQSAPS	21583	P51669	IAREWTQKYAM	21563
O60609	TLPLILLLSLW	21605	P57678	RQTLLQKMSSF	21583	Q92546	APGPSTSTITI	21563
P22732	LKELPPVTSEQ	21605	P50897	WFYAHIIPFLG	21583	O75487	ILFLVMQREWR	21562
Q9UKU6	LFQWLGKALRH	21605	P22674	LVPGPAAGPLP	21583	P51587	KQDTITTKKYI	21561
P02023	GVANALAHKYH	21604	P20160	GVLNNPGPGPA	21582	P54652	ASGGPTIEEVD	21561
P12324	LDQTLLDLNEM	21604	P31274	MKKMNKEKTDK	21582	O14829	DLMKPDVTNLG	21561
O14764	VNVIYWAAYAM	21603	Q16342	FVWKQDVTDTP	21582	Q05932	VLKLLEPALSQ	21560
P41247	VKFLLKENWFE	21603	P04085	KKRKRKRLKPT	21582	P53539	DPLNSPSLLAL	21560
P15515	GDYGSNYLYDN	21603	P51800	ISNLTNPPAPK	21581	P26572	PTWEGYDPSWN	21560
Q9UII2	KQKIKMLKHDD	21603	Q92847	SRAWTESSINT	21581	P20936	QNQYTKTNDVR	21560
P11309	HLHSLSPGPSK	21603	P05412	QLMLTQQLQTF	21580	P49662	MTRYFYLFPGN	21560
Q14449	KLKHYCARIAL	21602	P15391	GGGRMGTWSTR	21580	P40306	VEETVQAMEVE	21560
P02008	VVSSVLTEKYR	21602	P07199	QAGVRGLGHQS	21580	O88386	GGVTGWKSKCC	21560
P56846	VAAGPNPTSVM	21602	P15976	TSTTVVAPLSS	21580	Q08188	IKAMLSIDVAE	21560
P04075	SESLFVSNHAY	21601	P14902	STTEKSLLKEG	21580	P17480	SSGDSSDSDSN	21559
P15407	DPLGSPTLLAL	21601	P24158	TLRRVEAKGRP	21579	P54855	LAKTGKKKKRD	21559
Q16445	KDTMEVSSSVE	21601	Q9Y606	LEGSEGDGDTD	21579	Q13637	TLRAENKSQCC	21558
P26583	EDEEEEDEDEE	21601	Q9UF47	KEGSRSYCTDS	21579	Q92908	RPDSWCALALA	21557
Q9UL18	HQDTLRTMYFA	21601	P00519	SVKEISDIVQR	21578	P36269	DLRKSGEAAGY	21557
Q15631	NKETAAACVEK	21601	P22466	AAASSEDIERS	21578	P31270	EKKINRDRLQY	21557
P08047	LQSINISGNGF	21600	O00155	AQAANTASASW	21578	P48051	DVANLENESKV	21557
Q15417	DYQYSDQGIDY	21599	Q12988	VVEVKDPVGTK	21578	P01761	QGVLVTVSSGS	21556
P07919	CVAHKLFNNLK	21599	Q14571	TPHVNHHMPPH	21578	Q05472	FGKKKGPNANS	21556
Q14684	PRRRPRAMDFF	21599	Q92990	HVTTNGLQDHS	21577	P08069	RALPLPQSSTC	21555
P23416	KIIRHEDVHKK	21598	O75311	RHEDIHHQQQD	21577	O15225	GESASILGAVT	21555
P49863	IKSNLVPPHTN	21598	P01762	WGEGDLVTVSS	21577	P35232	AGQSVLLQLPQ	21555
Q02747	EICAYAACTGC	21598	P78337	ASGLNACQYNS	21577	Q13371	QKDLQEKISGK	21555
P10266	AKKRASTEMVT	21598	Q08379	DENDEVKITVI	21576	Q07020	RGRRASRGYKN	21555
P28074	DLHEKYSGSTP	21598	Q16550	AYKSRDTAIKT	21576	Q9NSI6	ENVLDFNGCTL	21555
P18031	LCYRFLFNSNT	21598	P25116	LNNSIYKKLLT	21576	P05062	TQSLFTACYTY	21554
O75954	HIHRTGKKYDA	21598	P02766	STTAVVTNPKE	21576	P11464	MTVEVSDWTVP	21554
P45378	PAKGKVGGRWK	21598	Q01081	RSRDRERSGRF	21576	P04629	QAPPVYLDVLG	21554
Q13362	RADELASQDGR	21597	Q09328	KGQVALCKDCL	21575	P26651	LPIFNRISVSE	21554
P02748	GLPALEFPNEK	21597	P35052	LALTVARPRWR	21575	P08962	VKSIRSGYEVM	21553
P00491	MASIPLPDKAS	21597	P51124	SWIRKVTGRSA	21575	P38405	QRMHLKQYELL	21553
P07101	DTLAHALSAIG	21597	Q99811	SLHHSQVPTVN	21575	P51161	VTYERVSKRLA	21553

ID: ID from Uniprot Database (http://www.uniprot.org/) **Sequence**: Listed sequences match the Uniprot ID, but for the array experiments and for the computational predictions, the cysteines were changed to serine. **NA**: not available. **BLU**: biochemical light unit.

Table S1: Binding data from CAL peptide array.

ID	Sequence	BLU	ID	Sequence	BLU	ID	Sequence	BLU
O43653	LGLLLWGPGQL	21553	P35218	SFQATNEGTRS	21530	P10264	NCPSPQAAVQQ	21509
P42166	CKVIKKRGNKH	21553	P43694	LADSHGDIITA	21530	P20618	IREETVSLRKD	21509
P35442	FSDLKYECRDI	21553	Q08648	PRWARGCSTGN	21530	O15537	MELLECVSKCA	21509
O15016	LASLTYWDKVP	21553	Q05519	DHHEEDMDMSD	21530	P29017	FKKHCSYQDIL	21508
O15544	PLSNFAFSYFP	21552	O43280	LPSLLLSLLPW	21530	P49721	LDNISFPKQGS	21508
P04216	SLLQATDFMSL	21552	P37235	LQCDPSSASQF	21530	Q99536	VLLVPGPEKQN	21508
O95670	PQVHPNYRISA	21552	O75390	GLMKFVDSKSG	21529	P21754	RTASHPVSASE	21508
Q15007	ELSAWKFTPDR	21552	Q9Y625	CIVLALQRLCR	21529	P01591	TALTPDACYPD	21507
Q9Y3Y2	VSPHLSYALTC	21552	P78417	QNSPEACDYGL	21529	Q15650	RRRLSVLQDLD	21507
Q9Y3C1	DSLQKRKMEVE	21551	P01033	CTWQSLRSQIA	21529	O00299	LAYEQVAKALK	21506
P06340	IMGTYVSSVPR	21551	P50616	SNQQFQPVMAN	21529	P31943	ENSSDFQSNIA	21506
P34981	CLASEVSFSQS	21551	P45379	RGKAKVTGRWK	21529	Q14589	QTAPQYSPKIN	21506
Q99598	TEMIDQEEGIS	21551	P47985	EFTSDDMVIVG	21529	P22352	RRQAALGVKRK	21505
P49842	TSGTLLRLPET	21550	P16991	SAPEAEQGGAE	21529	P06326	WGQGTLLIVSS	21505
O15552	EGMPSSDFTTE	21550	P07093	AVLFMGQINKP	21528	Q14088	QEANSKTSCPC	21505
Q15906	PRALRQKIVIK	21550	P52306	SLTEQRLTVES	21528	Q99611	DSSNASSEPSS	21505
Q15375	MLHLHGTGIQV	21549	P05538	PRGPPPAGLLH	21528	P16403	VKPKKAAPKKK	21504
O14607	FTLALSLSSSS	21549	P23246	EYEGPNKKPRF	21528	O60542	QLSAAACGCGG	21504
P35611	FLKKSKKKSDS	21548	P52657	DGKNTGSNTTE	21528	P26368	DPDSYHRRDFW	21504
Q08117	THQEDDGEKSD	21548	P01848	NLLMTLRLWSS	21528	Q9UL59	HLHNNHRRGNL	21504
P48506	KYSGSKTDSSN	21548	P25325	DVISEGRGKTH	21528	P24592	GSSSCPTGSSG	21503
P21266	KMAQWGNKPVC	21548	Q9UH06	YERKKYGFKKR	21528	Q9UJT0	VQDLPRLSIAM	21503
Q16676	VENFTARISNC	21547	Q9Y530	RYIYYLDWMWS	21528	P24752	GGASAMLIQKL	21503
Q99490	VGRADAPVALV	21547	O75715	ILAYLKQFKTK	21527	P40337	QERIAHQRMGD	21503
P26373	EAAEQDVEKKK	21546	P25788	KEEDESDDDNM	21527	Q93097	APKKAEWLDQT	21502
Q07283	YIQEQRSQYRP	21546	O00327	PVDFSDLPWPL	21526	P42684	CVQEISDVVQR	21501
Q13432	NKADYSYSGTP	21546	Q05925	TTTVQDKDESE	21526	O14558	SAQAPPPAAAK	21501
Q16186	KKDEEEDMSLD	21545	Q14654	PKFSISPDSLS	21526	P18283	PDIKRLLKVAI	21500
P09105	HVISALVSEYR	21545	P49903	IEVAHKWPLKT	21526	P30712	QAMLLRIARIP	21500
P08129	TPPRNSAKAKK	21545	Q92562	MYREYIRNRYL	21526	O14830	KDSGCSSPGAH	21500
Q9Y543	PPCGPGLWRPW	21544	P23415	KIVRREDVHNQ	21525	P23763	IVVVIVIYFFT	21500
Q99801	HCVGSWSPAFW	21544	P55895	AKKSFLRRLFD	21525	P54274	KLKLISSDSED	21500
P36537	FARKGKKGKRD	21544	P03979	LFGSGTTLVVT	21525	P18887	PHQLYGVVPQA	21500
P10589	FNWPYMSIQCS	21543	O14523	LCGIPGSLSAG	21525	P30679	LARYLDEINLL	21499
P28799	PLRDPALRQLL	21543	P30153	AQEALTVLSLA	21524	P05161	RGGGTEPGGRS	21499
Q99835	DTELMDADSDF	21543	P09471	IANNLRGCGLY	21524	P25098	PLVQRGSANGL	21498
O43193	TETSANVKTMG	21542	P01913	HSGLQPTGFLS	21524	Q9Y6D0	RGPSPPPMAGG	21498
P23083	SDDTVVYYCAR	21542	P45974	GYIYFYQRVAS	21524	P31391	KRIPLTRTTTF	21498
Q06136	REKSENADKTA	21541	Q92561	SCKTCNISVGR	21524	P00938	PEFVDIINAKQ	21498
P51878	LTRDFYLFPGN	21541	P22748	MLACLLAGFLR	21523	O75333	GVPGAWPGLPV	21498
P16562	KATCLCENKIY	21541	Q9UMX6	LAQQRRKSAMF	21523	Q13072	PEDGTALCFIF	21497
P55851	MAACTSREAPF	21541	P46091	TKNLCLLETAQ	21523	P33764	KDCPSEPPCSQ	21497
P07911	LLSATLTLTFQ	21541	Q01085	AGYGMASYQTQ	21523	Q92750	YSRALYLALLK	21497
P07099	IRKFLSVLERQ	21540	Q9Y2K6	EQKIEAETRAV	21523	P23567	SAIVEQSWNDS	21497
Q14863	NTSKLNVFQIP	21540	P01912	HSGLQPRGFLS	21522	O95399	ETPDCFWKYCV	21497
P04281	PPQGDKSRSPR	21540	P07510	GDPRPYLPSPD	21521	P32314	AKTQNQKQRKK	21496
P23411	LPPGLAVKELK	21540	O95450	EMRKKEMLGKF	21521	P05155	VFMGRVYDPRA	21496
Q15645	IEGFLQALSLA	21540	Q16602	NVLLKPENLYN	21521	P01569	ANLQERLRRKE	21496
Q9Y5P2	IEEVPGTKGSP	21540	P06468	LDQTLLELNNL	21521	P49720	ITTRTLKARMD	21496
P01771	WGQGVLVTVSS	21539	P38607	DIQQAFRNLED	21521	Q99460	EPPEPFEYIDD	21496
P04155	IDVPPEEECEF	21539	P06493	FNDLDNQIKKM	21520	O76024	DFFFFPFLSAA	21496
Q13653	PVLLGRLPFPH	21538	P25763	EPKKSRRCVLL	21520	O75695	FYNFADIQMGI	21496
P52951	SQHQQLEQARP	21538	Q14344	LHDNLKQLMLQ	21520	O95337	GRSPPGTSGST	21496
O14880	SGLGSGPKCCH	21538	P13807	SPTSSLGEERN	21520	P78333	ISVVMLLPGIW	21495
P48741	NRLVNHFMEEF	21538	P46597	GAIYDAILARK	21520	P49643	EGLEDYFSEDS	21495
P12751	AIRRLKELKDQ	21538	P06280	NTMQMSLKDLL	21519	Q16288	KATPIYLDILG	21495
P57075	AFNWRNWISGN	21538	P78396	LMEPPAVLLLQ	21519	O15529	GTGGQVACAEN	21494
P26374	NLESPEKHLQN	21537	P49674	QTSVPFDHLGK	21519	Q13636	KPTMQASRRCC	21494
P39027	HTGGKILGFFF	21537	Q13488	LSPFTFAATDD	21519	P52788	VFYTVWKKAKP	21494
Q13227	QHSQNPRFYHK	21536	P57081	KMRPGEATLSC	21518	P51571	LAFSAKSHIQA	21494
Q08257	SGATGKMILLL	21536	Q9Y4E6	AHDGKEHRFMV	21518	P21580	NECFQFKQMYG	21494
P49427	DDEDDSGTEES	21536	Q14691	IRQGVLEHILS	21518	P51580	LFEKLYLLTEK	21494
P35544	TLEVAGRMLGG	21536	P36404	DDISSRIFTAD	21517	Q15649	GIVEPSQNEES	21494
P01780	MDVWGQGTPVT	21535	O95429	AILEKLEKKGL	21517	Q9Y4E8	DIENENCMHTN	21494
P10696	TLLLLGTATAP	21535	P30711	KLMPWVLAMIR	21517	O75330	RAPMECQESWK	21493
O00422	APPPSGRMRPY	21535	P11465	RIGLLPLLNPT	21517	Q14320	PEKKWDKYTIR	21493
P30154	AQEAISVLALA	21534	P08708	GMNFKTPRGPV	21517	P35998	FSATPRYMTYN	21492
Q16772	ALEEARKIFRF	21534	O75317	SGYILFYQSRD	21517	O95859	SFNTHFEMEEL	21492
P02814	PGIFPPPPPQP	21534	P21181	ETQPKRKCCIF	21516	P48788	EGRKKMFESES	21492
P31939	LAHTNLRLFHH	21534	P01148	LIEEETGQKKI	21516	O95696	GEPTSDLSDID	21491
Q9UN79	PPVPSGFSGSM	21534	O76070	EVAEEAQSGGD	21516	Q00535	QHPYFSDFCPP	21491
Q99727	LRKEFVDIVQP	21534	Q04206	DFSALLSQISS	21516	Q14457	LAWVSSQFYNK	21491
O75643	VKEAETDSDSD	21534	P08727	HYNNLSASKVL	21515	P28065	LGNELPKFYDE	21491
Q12849	ELFLNSCPKGK	21533	P05154	NILFLGKVNRP	21514	P11488	IKENLKDCGLF	21490
O60841	IVELKKVFEII	21533	O43236	HKIQKQMKENY	21514	P32971	VLSIFLYSNSD	21490
Q9Y342	AATSQMAGGYA	21533	P11441	VTETMEKGFSK	21514	P08575	GPASPALNQGS	21489
P50453	SILFCGRFSSP	21533	Q9UK10	DTLLSLFLNDT	21514	P07492	REGRNPQLNQQ	21489
P30054	YAKDIGFIKLD	21533	Q92729	ALEYLEGLESR	21513	O15372	FMAQALQEYNN	21489
Q99541	QETQRSEHKTH	21532	Q02094	DSVYWKVPKTR	21513	P04156	LISFLIFLIVG	21489
P34925	TEFHAALGAYV	21532	Q14781	TSVGFFNLRHY	21512	P19438	AALPPAPSLLR	21489
P40200	SDLPYHEMETL	21532	Q14119	LPQALPTSPPW	21512	P51965	MARQWTKRYAT	21489
P53007	VKLLNKVWKTD	21532	P51654	ISVVCFFFLVH	21511	Q15041	KLLKQKEKKNE	21489
O75841	GTMFYWSRIEY	21532	Q9Y4H2	PAPCPTTYAQH	21511	P49682	SETSEASYSGL	21488
P42331	VKSMKEPKTEA	21532	P47210	EKNMSIKKLWK	21511	P55347	GLVLENSDSLQ	21487
P48167	FFNVIYWSIYL	21531	P35626	PSLCHRNSNGL	21510	P07737	EMASHLRRSQY	21487
P48556	IEYARQLEMIV	21531	P45973	AENKEKETAKS	21510	P42167	NFLHVDPRKSN	21487
Q9Y5X2	CSPPEDGLCPH	21531	P43080	EGADEAAEAAG	21510	O14788	YFGAFKVRDID	21487
Q9Y2L1	GPKKKKMKLGK	21531	P48552	GSVLTIKKESE	21510	Q9UMR3	FRDSSRLTDIE	21487

ID: ID from Uniprot Database (http://www.uniprot.org/) **Sequence**: Listed sequences match the Uniprot ID, but for the array experiments and for the computational predictions, the cysteines were changed to serine. **NA**: not available. **BLU**: biochemical light unit.

Table S1: Binding data from CAL peptide array.

ID	Sequence	BLU	ID	Sequence	BLU	ID	Sequence	BLU
P41182	TDLPPELPKAC	21486	Q92558	DSEFDEVDWLE	21463	Q16651	LGLLSPWLSEH	21442
O75419	KFLDALISLLS	21486	P16389	YVNITKMLTDV	21461	O94822	STCPLCRETFF	21442
O95249	CTILLLLYAFH	21486	P16104	GKKATQASQEY	21461	Q99929	LLDFSSWLGGY	21441
P36551	EVLRHPRDWVR	21486	P01236	LKCRIIHNNNC	21461	P06396	PLDRAMAELAA	21441
P17096	GISQESSEEEQ	21486	Q9UBK7	TPSEEVASPHS	21461	Q15654	QELSATVTTDC	21441
Q07325	KSQRSRQKKTT	21486	P27449	GLIVALILSTK	21461	Q9Y316	VSYAAGALTVH	21441
Q14695	KRGQSETFNIC	21486	P24557	KNGVYIKIVSR	21460	Q13057	SLPNTLVFGQH	21441
Q16635	EQLHNHLQPGR	21485	P51966	TKKYGEKRPVD	21460	O15541	EDPDEDAIPIT	21441
P09327	QQNLKKEKGLF	21485	Q15853	NLEMVGEGTRQ	21460	Q14627	PKMIPEFFCDT	21440
P19440	DSRKGGEPAGY	21484	P10620	AYRLLKSKLYL	21459	P47992	QSTNTAVTLTG	21440
Q14199	RKKRSKDFSGQ	21484	P01914	HSGLPPTGFLS	21459	P28347	AQHHIYRLVKD	21440
P22304	QGGDLFQLLMP	21483	P09429	DEDEEEDDDDE	21459	P21205	LFLEVFEDQEV	21440
P40238	SYLPLSYWQQP	21483	P20701	EKDSESGGGKD	21459	O14556	DLLRYMFSRDK	21439
Q9Y3B3	DKRTTTTRVGS	21483	Q02878	TNGIYPHKLVF	21459	Q92786	SPNCLQELLHE	21439
P11172	AWEAYLSRLGV	21482	Q99969	QFAFSKALPRS	21459	P01270	DVNVLTKAKSQ	21439
P01137	SNMIVRSCKCS	21482	P28161	VFTKMAVWGNK	21458	P51693	NPTYRFLEERP	21438
P08582	ALAARLLPPAL	21481	P28001	KTESHHKAKGK	21458	O15263	GLPGTKCCKKP	21438
Q9Y5T5	QAYLLFYERIL	21481	O75367	YVQEMAKLDAN	21458	O75879	IKEILEKKLSL	21438
Q14699	EVRELGTVEEN	21481	P41273	IPAGLPSPRSE	21458	P02096	AVASALSSRYH	21438
P39086	GIRKQSSVHTV	21479	Q15633	LQYLKIMAGSK	21458	P04062	YSIHTYLWHRQ	21437
P14770	GLLCATTEALD	21479	P57087	NDFKHTKSFII	21458	P40222	ELRSLCCSICS	21437
Q15695	RDRTVQSPQSK	21479	Q9UHD4	QWQQKGRLHSY	21457	P55808	NCFRTHEPENV	21437
P06133	FVRTGKKGKRD	21479	O15488	RIQEKLDRFLQ	21457	Q9UPC5	AVKIQSSSKST	21436
P48060	QLKYPNLVLLD	21478	P26927	VDWIHKVMRLG	21457	P55795	ENSSDYQSNLA	21436
P56915	EEGKSDLDSDS	21478	O43242	EMAEDDDDSFP	21457	P01857	TQKSLSLSPGK	21435
P51784	SPPSSEFMDVN	21478	P53803	RTKRLVVFDAR	21457	P13862	SNFKSPVKTIR	21435
P21283	YKIDCNLLEFK	21478	P08112	SNMIVKSCKCS	21457	Q9Y5K2	TEWIEKTVQAS	21435
P17405	AQSLWPRPLFC	21477	P09936	RFSAVALCKAA	21457	O00631	LMWLLVRSYQY	21435
P31358	ANAIIHLFCFS	21477	P08263	SLEEARKIFRF	21456	Q99961	SYVEVLVPLPQ	21435
P78415	ITKMTLTQVST	21477	Q9UBX0	AKKNFNTNLLE	21456	P20396	RAAWVREPLEE	21435
P20336	QQVPPHQDCAC	21477	O43426	KASPTLDFTER	21456	O95935	LSSSQVSAHMV	21435
Q13285	LLIEMLQAKQT	21477	Q16762	SRVSQGKSEKA	21456	P16662	FARKAKKGKND	21435
O14609	DIQSYTTDFSF	21477	Q07131	IVSGNLRILDH	21456	P30530	SPAAPGQEDGA	21435
P80404	NIFSDILADFK	21476	O60908	IMAAPSAWFLE	21456	P49910	QRIHMRENLLM	21435
P51451	TATERQYELQP	21475	P23769	PHPSSMVTAMG	21455	Q9UEY8	KKNKKKEKVEA	21434
P02100	AVAIALAHKYH	21475	O43502	RKRSRDPEEEL	21455	O95394	GGIGERPQPGF	21434
O75874	NLKIKLAQAKL	21475	P08886	QGGQQAGGGCC	21455	O00303	QIALNEKLVNL	21434
P43686	KKDEQEHEFYK	21475	P50748	EILKMFLSGLS	21455	Q15109	PEAGESSTGGP	21434
P04003	SARQSTLDKEL	21474	Q92851	RPPMRRWSSVS	21454	Q13642	QVYCPDCAKKL	21434
O60583	DSLLSAQGMNM	21474	Q13523	ALQHAFIQEKI	21454	P55327	EPLPEKTQESL	21433
P29016	YMRRRSYQNIP	21474	P43378	SGQNLLAVESQ	21454	Q9UMW8	AYLLVYMKMEC	21433
Q06547	MTRLQTNKEAV	21474	Q15286	TKNSKRKKRCC	21454	P15498	NYVEEDYSEYC	21433
P11021	GEEDTAEKDEL	21474	Q00341	APKTLPWGPKR	21454	O15091	PTKWLCLHQKT	21433
Q92604	NIIQYFYHCLF	21474	Q13347	DPQYFEFEFEA	21453	Q16559	VPSPGPSHHIP	21432
P09341	IEKMLNSDKSN	21473	P37290	LRMMDGDLSSP	21453	P16157	HSKDHTSTPNP	21431
P50219	PPRPSHQPAPQ	21473	Q16665	EELLRALDQVN	21452	P56180	MTSSDVVAGSD	21431
P16949	SKDPADETEAD	21473	P17861	RHQPSWKPLMN	21452	Q02547	RAKIDNYIPIF	21431
P30926	ASEGPYAAQRD	21472	P29992	LQLNLKEYNLV	21451	P15822	DDDEDRLVIAT	21431
P55259	VLLTVLLAWLF	21472	P27694	LVMSIRRSALM	21451	Q14549	QHQQMEQGARP	21430
Q99878	PKKTESHHKTK	21472	Q92993	TPKDWSKRGKW	21451	P10144	VHWIKKTMKRY	21430
Q93099	TPNSRNPAEPN	21472	P17013	KHQRTHTGQKP	21451	P53801	EENPYARFENN	21430
O75822	GGYVQDYEDFM	21472	P56377	PRSVLEEIGLT	21450	P46779	KRKRTRPTKSS	21430
P08519	VTWIEGMMRNN	21471	Q9UKS7	SHIVRGEHTFH	21450	Q02763	AGIDCSAEEAA	21430
Q09155	ARPGASPTPAC	21471	Q13686	EVKRARINPDS	21449	O43504	GITVAVHKMAS	21430
Q9Y285	EPRPPPTQEAA	21471	P05549	KSSDKEEKHRK	21449	Q14140	LDHIMEVLVGS	21430
P20366	RSAMQNYERRR	21471	P43304	PIPVDRSCGGL	21449	Q92524	KLESKLDYKPV	21429
P35916	ESRHRQESGFR	21471	P22415	GLEVVIKNDSN	21449	P47901	DGEGTAETIIF	21429
P33172	PSKKPVADYFL	21470	P42285	RDIVFAASLYL	21449	P51684	ADNDNASSFTM	21428
Q16620	KASPVYLDILG	21470	P53990	SRRFEELKKKT	21449	P32248	VEAETTTTFSP	21428
O75787	YRMTNQKIRMD	21470	P46439	LFGKSATWNSK	21448	Q04609	QAAAETLSEVA	21428
P15880	QRTQAPAVATT	21469	P11142	ASSGPTIEEVD	21448	Q03393	TDNNIVVYKGE	21428
Q15397	GIEILLEKLST	21469	P48147	IARCLNVDWIP	21448	P40855	PGASGEQCLIM	21428
P01019	LGRVANPLSTA	21468	Q15814	LPEEERNIQWD	21448	Q99714	IRLDGAIRMQP	21427
P18433	IDAFSDYANFK	21468	P37288	SKSIKFIPVST	21448	P06400	DSMDTSNKEEK	21427
P53816	VMFSRNKRQKQ	21467	Q13065	NCFLNLSPRKP	21447	Q15771	SISYLTCCNFN	21426
P35716	EANFSDLVFTY	21467	P52823	SHIKRTSHESA	21447	P28370	KTPMVKFSAFS	21426
P07814	AKYYTLFGRSY	21467	P55916	MKVQMLRESPF	21447	O43435	VAGRTAGDRLC	21426
P32970	ETFFGVQWVRP	21467	Q14004	VKSEEDRSRWA	21446	P54219	EDSDEEPDHEE	21426
P35125	ESDYEKYSMLQ	21467	Q9P0M6	YVQEMAKLDAK	21446	P17031	EHQRVHTGEKP	21426
Q92187	LKLTTGKCVKQ	21466	P27987	LTEMSQDAPLA	21446	O95843	DKAGLGKVKMK	21425
Q15382	ASQGKSSCSVM	21466	P51665	DVKKEEKKEKK	21446	O43765	TPSASNDDQQE	21425
P19065	ILIIIIVYFSS	21466	P01375	SGQVYFGIIAL	21446	P23381	MTPRKLSFDFQ	21425
O60664	GITEKAPEEKK	21466	O15482	SKDSGPPADGP	21446	Q99757	QLEAFLKKLIG	21425
P52735	PSTYVEEEGIQ	21466	Q06545	LPGVLCRSHPK	21445	P30291	KMNRSVSLTIY	21425
P10412	AKPKKAAAKKK	21465	P41235	PQPTITKQEVI	21445	Q9ULK2	TNRTGRIRTLP	21425
P08833	PNCQIYFNVQN	21465	Q92674	RAEGINCNNQY	21445	P08571	LVLLQGARGFA	21424
P55210	SMLTKELYFSQ	21465	Q9UL17	AEGQFYNYFPN	21445	Q99958	AAPYSYDCTKY	21424
Q9UD71	PQRPSPSEPGR	21465	Q08623	QPELFGLPSYE	21444	Q92769	KGTKSEQLSNP	21424
P35612	KKSKKKEKVES	21464	P20719	SMAAAGGAFRP	21444	P28360	AHVGYSMYHLT	21424
P32302	ESENATSLTTF	21464	P00995	TSILIQKSGPC	21444	Q15391	GNTTLESTDTL	21424
P06126	LALWFRKRCFC	21464	Q15262	YDVALEYLESS	21444	P17080	TTALPDEDDDL	21424
Q02127	VTDAIGADHRR	21464	P38606	DMQNAFRSLED	21444	P07602	AVEHCKRHVWN	21424
Q15583	AEMELQAKLTA	21464	P09002	CTGDCWPFRWS	21444	P15157	DWIHHYVPKKP	21424
P29144	PIMYPPDYCVF	21464	P10912	VSTDQLNKIMP	21443	P78552	VLIENLKKASQ	21423
Q92890	GQSLRKKGRKP	21464	Q99081	LSETTNPMGHM	21443	O14818	ENEKKKQKKAS	21423
P23443	SKRPEHLRMNL	21463	Q9UHL8	NSRLQRERANM	21443	P07988	SPLQCIHSPDL	21423
P22531	PCQPKCPPKSK	21463	O00559	KEQNKIGVKLS	21443	Q13207	RALSPGRESPK	21423
P56703	TEKRKEKCHCI	21463	P05204	AQKAEGAGDAK	21442	P25787	TEVKDYLAAIA	21422

ID: ID from Uniprot Database (http://www.uniprot.org/) **Sequence**: Listed sequences match the Uniprot ID, but for the array experiments and for the computational predictions, the cysteines were changed to serine. **NA**: not available. **BLU**: biochemical light unit.

Table S1: Binding data from CAL peptide array.

ID	Sequence	BLU	ID	Sequence	BLU	ID	Sequence	BLU
P23467	EYHRDPVYSRH	21422	P09486	KQKDIDKDLVI	21402	P17481	WKKENNKDKFP	21381
O14653	VVMFLVVQYLT	21421	P12081	KRRTGQPLCIC	21402	P01111	QGCMGLPCVVM	21381
P18564	EKQKVDLSTDC	21421	O15119	AKPDRSRSASP	21402	O60880	REDPDVCLKAP	21381
P01112	PGCMSCKCVLS	21421	P23193	CNECGNRWKFC	21402	Q03188	ESVLLFTQIKR	21380
P01850	VLMAMVKRKDF	21421	P52209	GGTVSSSSYNA	21401	P04440	RRSKKVQRGSA	21380
P22309	RVKKAHKSKTH	21421	P05556	TTVVNPKYEGK	21401	P17812	TELKFPSINHD	21380
P26378	VSFKTNKAHKS	21420	Q99437	ILQTSRVKMGD	21401	P18754	TVLLVKDKEQS	21380
P19075	SMVLYCQIGNK	21420	P15151	SSQDPQTEGTR	21400	P04053	LDYIEPWERNA	21380
Q03431	ALLQEEWETVM	21419	P11498	LEGDDLILEIE	21400	P17980	AKKKANLQYYA	21379
O75771	EQSATLQGDQT	21419	P35717	LRARHLRDYPD	21400	P26022	IQPHGGAQYVS	21379
Q92556	PSNYDFVYDCN	21419	Q15008	RVQKLSRVINM	21400	Q15031	RTALINFLVQD	21379
P54840	GKKKLHGEYKN	21418	Q92537	YTDDIPLLKEA	21400	P78381	LPKLLTKVKGS	21379
P31271	KKVINKLKTTS	21418	P55107	PNMSVDTCACR	21399	P01919	IRQRSRKGLLH	21378
P18850	HVVSTIPESLQ	21417	P00354	VDLMAHMASKE	21399	Q9ULC3	NRNPFSSCSIP	21378
O43837	SVIGHLQTKGS	21417	P27918	PACKDPEEEEL	21399	P18577	FWKFPHLAVGF	21378
Q03169	SRPLFSLIKVG	21417	Q05209	KGPRDPPSEWT	21399	Q16650	ISGYYGFYSHS	21378
Q16222	NGVHELVKNGI	21417	P25791	IYEWTKINGMI	21399	Q99735	LNIAKKLRRQF	21377
O00744	CKVTEWVNVCK	21417	O75949	PALEEGLTREE	21399	P01563	TNLQESLRSKE	21377
Q9Y284	LQNPQPMTPPW	21416	P21506	YQTNHIRENAY	21399	P13726	SWKENSPLNVS	21375
P01343	RGSAGNKNYRM	21416	P17516	DHPDYPFSDEY	21398	Q15672	MEGAWSMSASH	21375
P29350	KEKSKGSLKRK	21416	P01825	WGQGSLVTVSS	21398	O15078	EEESPVNFPIY	21375
P10082	VRSRSEGPDLW	21416	P04070	DKEAPQKSWAP	21398	P13796	ACLMGKGMKRV	21374
P20132	EQLGMTNRLPK	21416	P22735	ETIPMASRGGA	21398	P42830	QKILDGGNKEN	21374
P50749	LEEIAETPATI	21416	P40225	YTHSQNLSQEG	21398	Q92574	MDYNETHHEHS	21374
Q14839	EPTPQQVAQQQ	21415	P25490	ILTHAKAKNNQ	21398	P49454	ESKGSENCKVQ	21373
Q9UDY4	KEVLRKHLPAS	21415	O15342	ETIWYLKYHWP	21398	Q9P003	LYSMILALIND	21373
P20337	TPPLLQQNCSC	21415	P19622	TTAKEGKSDSE	21397	P80217	PRTAGPSSLHL	21373
P15927	DDDHFKSTDAE	21415	Q06203	LTGKYPVELEW	21397	O15056	ALQVFDPLAKT	21373
P51668	HAREWTQKYAM	21415	P98175	KTMVTRFNEAQ	21397	P01918	IHHRSQKGLLH	21372
O60811	PSEELELHLCC	21415	P49815	ISSVEDFTEFV	21397	P07225	CPSVWKKTKNS	21372
O95096	HPLVQAQQWTW	21414	Q08722	FKESKGMMNDE	21396	O00411	EQVKRSTYFFS	21372
Q14651	CLMGKGLNRIK	21414	Q13003	ACSTSLAPVFP	21396	Q15836	IIIIIVWVVSS	21372
Q16822	LEALERRVHKM	21414	O75821	LNVEWAKPSTN	21396	O60765	NHYKIHIEEDP	21372
Q15238	GIGRLPLLNPI	21414	P15260	YRPTEDSKEFS	21396	P41732	RFITANQYEMV	21371
P48380	RRDCGVIARVP	21414	Q16576	VTTSELEGQGS	21396	Q15056	PREEVVQKEQE	21370
O95343	SVTSSDSECDV	21414	O60810	SFYDLEADQYC	21396	P35244	QFYPLGIVQHD	21370
Q15750	DHGEQSVVTAP	21414	Q13794	NLISKLFCSGT	21395	O14894	GDCRKKQDTPH	21370
Q9UI12	SEQPQTAARS	21414	O14735	ALDAADRAKKK	21395	P31942	REMEEGEYEEA	21370
P49336	PPQYSHQTHRY	21413	P57059	PCSLGTFVLVQ	21394	P21695	IHCLQNHPEHM	21369
P09211	VNLPINGNGKQ	21413	P47986	ISREWTQKYAM	21394	P38646	EQKEDQKEEKQ	21369
P49840	TDATPTLTNSS	21413	Q05086	AITYAKGFGML	21394	P01344	GAPPEMASNRK	21369
P20933	NQPTEEKVDCI	21412	Q9Y2D2	DPKPAGNPTKA	21394	O43776	LYPRFVQRCTP	21369
P06756	PHENGEGNSET	21412	P34062	DAHLVALAERD	21393	P17542	MLPAADGAGPR	21369
P26010	PRFQEADSPTL	21412	P23468	EYLGSFDHYAT	21393	Q01831	AASHLFPFEKL	21369
P42681	LRAVTEIAETW	21412	Q9Y5S9	RRSRSPDRRRR	21393	O14802	DTNEFHIPLVT	21368
Q9UKP6	RPAPEGPRAPA	21412	P21675	IAGDSDLDSDE	21393	NA	WGQGDLVTVSS	21367
Q15389	STTMMIRPLDF	21411	Q93075	LRENTSRLYSL	21393	P29373	DVVCTRVYVRE	21367
P40429	TEVLKTHGLLV	21411	Q03924	STLTPHKTIHI	21393	P29377	EFQVLVKKISQ	21367
Q07244	NSVKQYSGKFF	21411	P78423	CGSNSYVLVPV	21392	O14524	CPAITQNNFLT	21367
P10646	IAYEEIFVKNM	21411	O60907	DGSVCVLDLRK	21392	P11215	SEGGPPGAEPQ	21366
P31483	SGYRVAGYETQ	21411	P09110	GAAAVFEYPGN	21392	O95716	APAPQPSSCSC	21366
O00294	LSSFDGKLACE	21411	Q00613	EPPKAKDPTVS	21391	Q16890	SRRTKEEELQC	21366
P17066	PSTGPIIEEVD	21410	Q15714	QPASQGSGPTA	21391	Q99684	RRHRETQHGLK	21365
Q9UBY9	VQQTFRTEIKI	21410	P22314	VEVPYVRYTIR	21391	P16401	PKAKKAAAKKK	21365
O60613	EEFLSEKLERI	21410	Q05877	AVVQFEQHKRH	21390	Q9UKV0	YDPLMLKKPNS	21365
P10600	SNMVVKSCKCS	21410	P42574	SMLTKELYFYH	21390	Q01804	EISDSEDDSCK	21365
Q15653	ERQGSPAGGSG	21410	P30048	ASKEYFQKVNQ	21390	P01766	YYGSVWGQGTL	21365
P04035	DLQGACTKKTA	21409	P35523	TDEEDEDELIL	21389	P31260	IRELTANFNFS	21365
P19823	VPQLYSFLKRP	21409	P56537	SMRDSLIDSLT	21389	Q13185	TWHSCPEDEAQ	21364
P00747	VTWIEGVMRNN	21409	Q92748	RKYQEMTGQVW	21389	Q15849	IITKYQAYDVS	21364
Q13049	YSYHLRRYSTP	21408	Q08378	DLSMTQKDKFM	21388	O95947	GYLDVGSKPMY	21363
P04280	PQGGRPSRPPQ	21408	P17693	AAVLWRKKSSD	21388	P36021	LPGSPNPEEPI	21363
Q14188	EDDEEDSSSPE	21408	P52926	ETSSQESAEED	21388	P10159	EAAVAIKAMAK	21362
P14317	LFPANYVKLLE	21407	P49768	MDQLAFHQFYI	21388	P51151	HRKPKPSSSCC	21362
P34931	PATGPTIEEVD	21407	Q12913	TTFGKTNGYIA	21388	P38567	LFLIISSVASL	21361
O95755	ESKRPSSLGCC	21407	P15918	DSLESQDSMEF	21388	Q13418	IVPILEKMQDK	21361
P48307	SRIRKIRKKQF	21407	P31629	KDPSSEKSQLH	21388	Q15235	GPCHGNQTESH	21361
Q9UDW1	WKHIKHKYENK	21407	P10071	KRLQPTEINRS	21387	P23469	IDIFSDYANFK	21361
P36543	FGANANRKFLD	21407	O14842	AARTQGGKSQK	21387	P54578	RVEIMEEESEQ	21361
O75325	ETLLPPLSQNS	21406	P06401	GMVKPLLFHKK	21387	O14929	YRRVIERLAQE	21360
P35247	CGEKRLVVCEF	21406	Q15431	KKLKEAEKLFV	21387	P04554	CRTRKRTCRRH	21360
Q13156	TVDREHFKSAD	21406	P19532	SPGGLSSAPSP	21387	Q15651	QKTESVDNEGE	21360
Q92843	LVTVGAFFASK	21405	P05106	STFTNITYRGT	21386	P30518	ASSSLAKDTSS	21360
Q9Y5L2	KGLPDHPSRSM	21405	O00198	LAAWLLGRRNL	21385	Q9NNW5	GQGLEVYNWYD	21360
P35475	VPRGPPSPGNP	21405	P10599	EKLEATINELV	21385	Q12788	PVTPESSSGRM	21359
O60749	EAFLPEAKAIA	21405	O95067	KDLASPLIGRS	21384	Q92664	MLSTVAVLTLG	21359
Q9Y3A5	KDVEEGDEKFE	21405	Q00889	PNTWFQEILLL	21384	P35590	YAGIDATAEEA	21359
P32246	STGEHELSAGF	21404	P06366	RRKGGRRGRRL	21383	Q15006	DMLETLQITQS	21359
P07203	EALLSQGPSCA	21404	P21731	PQPPEQLGLQA	21383	O60320	AVFGADVLDQV	21358
Q16777	GAPVYMAAVLE	21404	Q01995	TGYGRPRQIIS	21383	P50591	EASFFGAFLVG	21357
O15533	STCKDSKKKAE	21404	Q93074	PQPSTNIFGRY	21383	P48049	EPRPLRRESEI	21356
Q00534	PSQNTSELNTA	21403	P47756	LVEALKRKQQC	21382	Q99456	SSQVQEIEELM	21356
P05231	LQSSLRALRQM	21403	P55212	TKKLHFFPKSN	21382	P25789	KKEKEQKEKDK	21356
Q99719	LQRMKQQMQDQ	21403	P35625	PDKSIINATDP	21382	P48651	KSKVTNGVGKK	21356
Q9NP85	LNPKKKDSPML	21403	Q14154	LERSVVRLGFG	21382	Q9UBS0	KSKRGRGRPGR	21355
O75469	LMQELFGITGS	21403	Q99873	ELSCSTDYRMR	21381	P10619	MFSRFLNKQPY	21355
P21980	KGFRNVIIGPA	21403	Q92754	KTLEKMEKHRK	21381	Q15293	GEDLTKNHDEL	21355
O14807	TGTHKLQCVIL	21402	P01818	WGQGTKVAVSS	21381	P51809	GGFTWPSCVKK	21355

ID: ID from Uniprot Database (http://www.uniprot.org/) **Sequence**: Listed sequences match the Uniprot ID, but for the array experiments and for the computational predictions, the cysteines were changed to serine. **NA**: not available. **BLU**: biochemical light unit.

Table S1: Binding data from CAL peptide array.

ID	Sequence	BLU	ID	Sequence	BLU	ID	Sequence	BLU
P55789	WRDGWKDGSCD	21354	Q14689	QLDPIYVAYNM	21329	Q13887	HLALHMKRHQN	21306
Q92481	KTGDKEEKHRK	21354	P08603	GKLEYPTCAKR	21328	P29374	PPQNVLAVECR	21306
P40928	YPGEEDGGNGG	21354	Q06710	PPTTATAFDHL	21328	O43927	LPVPVFKRKIP	21306
Q04637	FKWLREVRGGV	21353	P35287	EPQPQREGCGC	21328	P05452	QLPYICQFGIV	21306
Q16557	GTGHLPGLNPL	21353	P43308	RKYDTPKTKKN	21328	P48735	KSNLDRALGRQ	21305
P10827	CEDLAGNAASP	21353	O95715	AWNEKRRVYEE	21328	P49848	QPNSGSPQPAP	21305
P02749	WKTDASDVKPC	21352	P42768	GDEDEDDEWDD	21328	P23258	PDYISWGTQEQ	21305
O15499	PGVKKSPKGSC	21352	O60543	RSQAKGRFTCG	21327	O15027	RIGQRKHLVLN	21305
P09488	VFSKMAVWGNK	21352	P51553	IRVINGRAVEA	21327	P17033	EHQRIHTGETP	21305
P22492	EVNVRKATSKK	21352	O75840	DHLALHMKRHI	21327	P29460	SWSEWASVPCS	21304
P08397	LDVARQLNDAH	21352	Q13118	IALPPTPAPTQ	21327	P57052	KFRKSKKKKRY	21304
P49589	GLKTTKDCPQW	21352	Q13442	GKRMQSLSLNK	21326	P09526	KARKKSSCQLL	21303
P52655	IFSKAIGDAEW	21352	Q15181	DVDKWFHHQKN	21326	O00399	TMKGSSTPVKN	21303
Q14157	PPYKHFWTAES	21352	P05783	SETNDTKVLRH	21326	P50613	EQGGLPKKLIF	21301
P13765	VSRAVLLPQSC	21351	P56705	CQRLVELHTCR	21326	P35900	SSEVKEVEENI	21301
P50991	ILKIDDVVNTR	21351	O00555	EPYSESDDDWC	21325	P06454	DTKKQKTDEDD	21301
P16471	LDPACFTHSFH	21350	P01571	TNLQKILRRKD	21325	Q06732	YECGKSFCMNS	21301
Q15561	AQHHIYRLVKE	21350	P14373	GNHGHSMETSP	21325	Q15842	TPEGNQNTSES	21300
O60507	LKEKPQTEQVE	21350	P07437	EDFGEEAEEEA	21325	P10636	EVSASLAKQGL	21299
P22460	LCLDTSRETDL	21349	P19484	DEGPPGYPDRN	21325	Q14588	NIRELSEGGSS	21299
P24593	QCHTFDSSNVE	21349	P17027	QHQSVHSEGKS	21325	Q03933	PLDSDMPLLDS	21298
P31144	YLGQHVHLGGR	21349	P51686	LLETTSGALSL	21324	P18065	EARGVHTQRMQ	21298
Q9UBK9	GLQNFPEKPHH	21349	P25786	PAEKADEPMEH	21324	Q15544	PNSKHKKIIFF	21298
P52744	YKCEECGKAFN	21349	P07029	FCFITFNQEEP	21324	P03986	RTAFCCNGEKS	21298
P08247	QGAPTSFSNQM	21348	P16035	PKQEFLDIEDP	21324	P35236	YAGQLPEEPSP	21297
P36402	SLSMSSSSSPA	21348	P52737	GPPYKCMWESL	21324	P22670	RGLFVQALPSS	21297
Q14186	DDDFNENDEDD	21348	Q15269	GGRDSEEEMLA	21323	P46778	LLEPIPYEFMA	21297
P01374	PSTVFFGAFAL	21348	P23131	PRIASNAGSIA	21323	P09629	DRAEAEEEEEE	21296
Q13569	GTQEQEEESHA	21347	Q13107	GDDEACSMDTN	21323	P13489	EKDKPSLRVIS	21296
P10828	FPPLFLEVFED	21347	Q06481	PTYKYLEQMQI	21321	O43819	RHMAAFRSVLS	21296
Q9Y5K8	AEEKDEDLLFE	21347	O43194	AAENGFQEHEV	21321	P49221	INAQKIVLITK	21296
Q92889	AEVVSKGKGKK	21347	Q92522	PSVPKVPKGRK	21321	Q9UK11	DIILSLFLNDT	21296
Q99676	LNKHQRLHPGI	21347	P98176	TTISQVAPGED	21321	P28749	LQDVVSERANH	21295
P17482	MKKMNKEQGKE	21346	O76061	DEQSEYSDIRR	21321	P51531	EQSEGSGTDDE	21295
O14901	GSPLVSMPASA	21346	O95544	FEEEEEEEEEG	21321	P49590	VAEIQKRLSES	21295
P38936	KRRLIFSKRKP	21345	Q9Y6M4	RRKRKTIQRHK	21320	P00749	SHTKEENGLAL	21295
P01834	PVTKSFNRGEC	21345	P14868	MFPRDPKRLTP	21320	Q14206	RRPGLPPSVSN	21295
P11473	LVLEVFGNEIS	21344	P15923	LSEAHNPAGHM	21320	Q06124	VGLMQQQKSFR	21294
Q9Y3B4	EKYGINTDPPK	21344	P10075	LIQHQRVHYRE	21319	Q9Y3F1	NQYFELAKFLA	21294
P52738	FNYHSFLTEHQ	21344	P49642	LLKKSDLQKDF	21319	Q12815	PVGSAAPQGSP	21294
P30991	TESESSSFHSS	21343	P01118	KKKSKTKCVIM	21319	Q13838	IDISSYIEQTR	21293
Q13308	ALGDSTVDSKP	21343	P25120	RGTKTVQEKEN	21319	P04843	VTKIDHILDAL	21293
P10114	DDPCCSACNIQ	21343	Q00994	HDHHDEFCLMP	21318	P35548	TPVGYGMYHLS	21292
Q14257	LHDDYFYHDEL	21343	O75570	ELLDEHLKSAK	21318	P31269	KKINKDRAKDE	21292
O14981	YSLENFMHSLK	21343	P02778	AVSKEMSKRSP	21318	P07949	SAAKLMDTFDS	21292
P25874	SKSRQTMDCAT	21343	Q92917	CTKEANITTPR	21318	P37837	TERMFNAENGK	21292
P43403	GSTQKAEAACA	21343	P55089	QNRIIFDSVGK	21318	Q16864	ARGMFTAEDLR	21292
P13762	HSGLQPTGLLS	21342	P08118	KKTCSVSEWII	21317	P01769	WGEGTLVTISS	21291
P25106	TEYSALEQNAK	21342	P48378	RSDPNHSLQGI	21317	O43657	RAITNNQYEIV	21291
O75069	MTQCLQEERYR	21342	P49798	ADCASLVPQCA	21317	Q9Y6Q3	VKTHSEDKSHE	21291
P05019	RNAECRGKKGK	21341	O76039	TYHENAALTGK	21317	P54255	QEDGKSHRQRS	21290
Q16514	RMALIRKTTKK	21341	O75083	DASVKEWTITY	21317	P35858	RDLSEAHFAPC	21289
P55072	YTEDNDDDLYG	21341	P00751	EKLQDEDLGFL	21316	Q14493	TEPLRDFSAMS	21289
P21246	KEGKKQEKMLD	21340	P55010	NKDDDIDIDAI	21316	P48643	DDIRKPGESEE	21289
P37173	IPEDGSLNTTK	21340	Q14012	TELSPTLPHQL	21316	O75562	PIDDKRWNLKA	21288
P35968	DSGTTLSSPPV	21340	P28066	KEELEEVIKDI	21316	P30511	GSGVSLTANKV	21288
Q14149	VVEQMSEISST	21340	P98179	SGGNYRDNYDN	21316	Q14774	PEPAQGALGCL	21288
Q92820	ISSFQQCYIFD	21339	Q15032	DFHILERASSQ	21316	Q99594	AQHHVYKLVKD	21288
P05215	DSYEDEDEGEE	21339	P13716	TPQLLQWLKEE	21315	Q92539	PIPEVDLDDLS	21288
Q14191	MDKTKRGGLFS	21339	P13747	DSAQGSESHSL	21315	P19367	VGVRLRTEASS	21287
NA	WGQGTLVTISS	21338	Q9Y580	SRDGKWRSSRH	21315	Q04756	IRPPRRLVAPS	21286
O43399	GDKPLSDPAPF	21338	P14118	KTLSKEEETKK	21315	Q12923	EEQKQQPQLLK	21286
Q13162	AGKLKYFDKLN	21338	Q9Y490	KFLPSELRDEH	21315	Q9Y4P3	ETLKSLGALKK	21286
Q06643	GKTFFGAVMVG	21338	P04275	ECKCSPRKCSK	21315	Q15562	KVETERAQLED	21286
P17947	GGLAERRHPPH	21337	P19544	QRNMTKLQLAL	21315	O43548	GYRNVYVDFAL	21286
Q9NSD9	SSLEINIGPFL	21337	P09565	LAGVGVPGLPV	21314	P17035	CHRRSHIDEKP	21286
P26640	DEAIALFQKML	21337	O43251	ATADLPPTEVT	21314	Q9NZL3	LHQNVHVGEKP	21285
O14773	FPALLKTLLNP	21337	P02403	KRAAVAASSSS	21314	P05107	TTVMNPKFAES	21284
P03992	IRQRSQKGLLH	21336	P35367	KTFKRILHIRS	21313	P22102	ENGKICWVKEE	21284
O75360	PLSLEPSKSWN	21336	P01764	AEDTAVYYCAK	21313	P29083	MGQRMFEDLFE	21284
Q00887	PCHGDLTESQS	21336	P19012	GQVVSSHKREI	21313	P01266	LQEPGSKTYSK	21284
P57082	GMGTVENWTDG	21336	P41250	QETGKKETIEE	21312	P25311	AQPLVVPWEAS	21283
P11686	TLCGEVPLYYI	21335	P11684	MEKIAQSSLCN	21312	O95239	SGCSPIEEEAH	21282
O14957	PYINGKFKKDN	21334	P49751	LQQSMPPCEPP	21312	P05543	FLGKVVNPTEA	21282
Q92901	EKETPETSGDL	21333	P10074	ITAAVPEDCDT	21311	O15273	SRSMSQEAQRG	21281
P19388	AGRYITYRLVQ	21333	O00219	PEQYSLAFAEV	21310	Q15054	QVSITGFFQRK	21281
Q9Y5M1	ENLVPATVWDG	21332	O14921	LLKTMQSNNSF	21310	P08887	DISNTDYFFPR	21279
P30038	PLGDWSYAYMQ	21332	O95988	LVLTYQPERKD	21310	P49841	NAASASASNST	21279
P42680	ELVECEETFGR	21332	P34913	DARNPPVVSKM	21309	P17931	IDLTSASYTMI	21279
Q9NYT6	RESTQEKKSIK	21332	P18621	KLKKQKLMARE	21309	O15539	VRSEFYQELIK	21279
P04553	RPRYRPRCRRH	21331	P35273	HRASHYQAHYT	21309	P43351	QDMKKRKYDPS	21278
Q15569	TPSLQLPGARS	21331	Q03167	QSTPCSSSSTA	21308	P49796	LINQKKMSPPL	21278
Q15004	LQPDHTNDEKE	21330	P20962	KRQKTENGASA	21308	O95707	KKFKAKGTIDL	21278
P06127	SDYDLHGAQRL	21329	P51786	SWRCTMKKASH	21308	Q15051	ENLFIGGTKPP	21278
Q03164	CGAKKCRKFLN	21329	Q01484	SDTEQSEDNNE	21307	O43493	HQWIVLLSFQL	21277
P54577	WRSTQMQTACM	21329	Q15046	TLESTTVGSSV	21307	Q14687	WPRGYLKGYPR	21277
P24821	FRNLEGRRKRA	21329	Q15049	SLAPSMETHNP	21307	Q15669	RLFSINECKIF	21276
O00258	KVVAIVLHPFS	21329	O15226	GHYELVMPQAN	21306	P51991	GGSGGYGSRRF	21276

ID: ID from Uniprot Database (http://www.uniprot.org/) **Sequence**: Listed sequences match the Uniprot ID, but for the array experiments and for the computational predictions, the cysteines were changed to serine. **NA**: not available. **BLU**: biochemical light unit.

Table S1: Binding data from CAL peptide array.

ID	Sequence	BLU	ID	Sequence	BLU	ID	Sequence	BLU
P13196	SGLSKLVSAQA	21275	P20702	GTQTPSPPSEK	21251	P29316	ALDVANKIGII	21213
Q9UNH7	QNCLAVLNGDT	21275	P17028	NAEKLLNVVKV	21251	Q15053	NQQGPHTPSIP	21213
Q9Y5X1	LRQALSRFPVM	21275	Q15833	LDKKLEDIALP	21250	Q92622	LALEAAVLEAT	21213
Q01658	SSQDEEDDDDI	21275	P13987	PFLAAAWSLHP	21249	O75916	EKEVICPWESL	21212
P37840	EEGYQDYEPEA	21274	P29074	FVKPLTTSTNK	21249	P20226	YPILKGFRKTT	21212
Q9NY65	SFEEENEGEEF	21274	Q08999	LQDVANDRGSH	21249	O95522	LLNACCQGGFI	21212
P52742	SLTKHQRTHTG	21274	Q99593	VGMVPEWSDNS	21249	P00797	RNNRIGFALAR	21211
O60563	EPPPPLPPLPK	21273	P20061	GENLEVRWSKY	21249	P15814	VEKTVAPAECS	21210
P30519	LAAGLLAWYYM	21273	Q92819	KGQQYDMVLDV	21248	P00568	SQVCTHLDALK	21210
P48549	KLRKMNSDRFT	21273	O43474	DHLALHMKRHF	21248	P41220	KPQITTEPHAT	21209
P07333	PLLQPNNYQFC	21273	P78332	RVMFARYKELD	21248	Q15436	HLKKLAVSSAA	21209
Q9UNH6	LHLEEASEDKP	21273	P32969	SEKGTVQQADE	21248	Q03519	QEGKLQKLAQL	21209
P47897	TVTLKEDPGKV	21273	Q16534	LAKYEARHGPL	21247	P49757	SDLQKTFEIEL	21209
Q13428	KKKKKTAEQTV	21273	P17987	EDAVHSGALND	21247	P04233	VTKQDLGPVPM	21208
O15393	DWIYRQMKANG	21273	P17030	VHQRMHTGEKP	21247	Q14541	SHQHLSKQKQL	21208
P24864	GKKQSSGPEMA	21272	P09001	CQPGAPSITFA	21246	P26012	LNAHETFRCNF	21208
Q92598	NEKNSVNMDLD	21272	Q15776	HSGEKSESISV	21246	P35241	TKQRIDEFEAM	21208
P07305	KSSAKRAGKKK	21272	P00737	QDWVQKTIAEN	21245	Q9Y473	CSYSVKGFTKQ	21208
Q13610	GSRSSDTPMES	21272	Q16143	QEEYQEYEPEA	21245	Q13319	GTKHWTMNLDR	21207
O75347	RLVLDSVKLEA	21272	P13682	LMRHHRTHLVE	21244	P13646	SGRRTSDVRRP	21207
P12270	GRGGINRGNIN	21272	P01767	FGKGTTVTVSS	21243	P27635	GPLDKWRALHS	21207
P52743	ALHAEHQKIHF	21272	Q09028	EGSVDPEGQGS	21243	Q9UKW4	WFPSTYVEEDE	21207
P51617	QGPEESDEFQS	21271	P24928	AISPDDSDEEN	21243	P01589	QRRQRKSRRTI	21206
P01166	KNFFWKTFTSC	21271	P46199	IQAKTSWDPGF	21242	P52199	RKDKAKSCTVM	21205
Q13907	QFVDHEKIYRM	21270	P55073	EQLHGARPRRV	21242	P17257	SGYLLGKVGNT	21205
P57729	KVASCSGCAKS	21270	P19827	AYTDYIVPDIF	21242	O00445	PPDRVRLLPAP	21205
P31025	RQSETCSPGSD	21270	P17936	EDVHCYSMQSK	21241	Q9Y2L8	INTLSVEGSLL	21205
Q15034	ALDNYEGFSLA	21269	P06870	KWIEDTIAENS	21241	Q14116	RSIMFTVQNED	21204
Q16600	SHQRVHKKDPR	21269	P10153	PVVPVHLDRII	21240	Q13237	PDELSGWDKDF	21204
P17037	QHQRVHTGKKP	21269	P00746	SYAAWIDSVLA	21239	O00139	KQINPKRPRAL	21204
Q13546	LLSSLIYVSQN	21268	P08631	TATESQYQQQP	21239	P30408	FCCSHQQQYDC	21204
P04004	YWLGCPAPGHL	21268	Q9UBN7	NKFGEDMPHPH	21239	O76090	WALENRDEAHS	21204
Q9Y6F9	RGHRQESVQLE	21268	P46940	IFLLNKKFYGK	21239	Q9Y3D7	EDREKWQMPHT	21204
Q14153	GELDIEQIEKN	21268	Q01664	REEPSGDGELP	21239	O00165	DLFLGRWFRSR	21203
Q13263	QELSGGPGDGP	21267	P20340	EQPVSEGGCSC	21238	Q01581	PEAAVISNGEH	21203
P51522	FHAGKKSNTCN	21267	Q9Y651	DPYPAAYAAAL	21238	P51153	DKKNTNKCSLG	21203
Q16667	SRDSQSRSVSR	21266	Q99816	ARKTAGLSDLY	21238	P98171	PQGLDTTPKPH	21202
P25021	EVTAPQGATDR	21266	Q12901	DILLSLFLNDT	21238	Q92543	PGNSKRMGVSS	21202
Q9Y3A6	SLFEDKRKSRT	21266	P35269	INDKMHFSLKE	21237	O43830	NKCGKTYSHKS	21202
Q9UL36	AGEWQALTHVF	21266	P13164	MLQIIQEKRGY	21236	Q01201	GGLLSPGPEAT	21201
Q15078	DKKRLLLGLDR	21265	P49895	EVRAVLEKLHS	21236	P21549	AALQHCPKKKL	21201
P38919	DEMPMNVADLI	21265	Q15545	LQEELESLLEK	21236	Q9Y5X0	GCKVNTAPQES	21200
P01116	GCVKIKKCIIM	21265	P04196	VSMFFTHTFPK	21234	P07686	LYAGYCNHENM	21199
Q13671	PEAEGSRAAEE	21265	Q16760	ELSRSAPAVEA	21234	P52848	TWLREDLQNTR	21199
Q9UKI3	YYCSVGYGFSP	21265	O94806	APNPDDMEEDP	21234	Q9Y581	EKRSSLVTKIY	21199
P31267	SGEDSEAKAGE	21264	P49802	SKRLTSLAQSY	21234	P52815	LEAVGGTVVLE	21199
O95754	APLATCDETSI	21264	O75324	LMTPNGPEVHG	21234	Q08945	SSEDSASGSDE	21199
Q9UK13	TSVSLCGRKAI	21264	P17483	PGRPNGGPRAL	21233	P30520	GKSRESMIQLF	21198
P20823	FISTQMASSSQ	21263	P51159	SEEKEKGACGC	21232	P34096	GNPQVPVHFDG	21198
P54829	YEKQLSHQSPE	21263	P01583	ITDFQILENQA	21231	P15622	ECGTPSVHAGL	21197
P06749	RGKKKSGCLVL	21263	Q92730	KKEKAKSCSIM	21231	P51798	RGLEELSLAQT	21196
Q00403	FDTPVDKLPQL	21263	O00212	RRITQGFCVVT	21230	P52741	RHQKVHTAGRL	21196
P36508	TLETTVSESGC	21263	Q99962	NYVEILVALPH	21230	P04687	EGEGEEEGEEY	21195
P17706	GWRLFFQQNAL	21262	Q92526	MIEFKINPSRR	21230	Q16836	KKTGEGFYKYK	21194
O94762	DALVTFCEELG	21262	Q03403	FFPKSVEDCHY	21229	P09067	SLATAGSAFQP	21194
O43593	KVAVGTLQEAK	21261	P49767	VPSYWKRPQMS	21229	P29084	LKDYSDITSSK	21194
P54868	QHRRKYARRPV	21261	Q14693	FENQDIHSASA	21229	P49750	SMFKTFGKDSG	21194
Q92782	KEKASAYITLT	21261	Q12962	GINVKKPHYFT	21228	O14978	RLMSHQRTHTG	21194
P01911	RARSESAQSKM	21260	P50750	TTNQTEFERVF	21227	Q9Y5A6	QRVHTGEGEAP	21193
O43665	AAKRASRIYNT	21259	P01233	PGPSDTPILPQ	21227	P09326	TVPTILGLLLT	21192
Q06633	VGATDLPGQEW	21259	O75578	EEEKREEKLEQ	21227	P78426	LLHASEPESS	21192
P07476	KQEVQWPPKHK	21258	P49810	MDTLASHQLYI	21227	P17964	DEGCCSACVIL	21192
P37802	MTGYGMPRQIL	21258	Q92540	QRGQGTMNPPH	21227	P17600	LRKSFASLFSD	21192
Q15697	LHQHHRLHHGD	21258	O43182	FSSSLPYLMFL	21225	P78371	PRKRVPDHHPC	21192
Q02224	SGKDVPECKTQ	21257	P49765	PDTCRCRKLRR	21225	Q9Y3E2	LGGNKKTLGTP	21192
Q9ULV5	SYLGPEASPSP	21257	P01562	TNLQERLRRKE	21224	O94761	LATEELLQVAR	21191
P13693	FFKDGLEMEKC	21257	Q15770	KPTMQSSRRCC	21224	P13683	VTTELNIREST	21190
Q16465	SLLPPLGSHGA	21257	P23677	LIGILASLAER	21223	NA	WGQGTLVTVSS	21190
P56524	DEEPMEEEPPL	21256	P01824	WGQGTTVHVSS	21222	Q99665	QLKMRCDSLML	21190
P48382	QEHKDPKATPP	21256	P05362	PMKPNTQATPP	21222	Q13547	AKGVKEEVKLA	21189
P49758	LYSNTPLAKRP	21255	P15884	MGDASNHMGQM	21222	P10072	NLIRHQRTHSG	21189
Q13509	DEEESEAQGPK	21255	P54725	NFLLSQNFDDE	21222	Q9Y6H5	SSASKGKNKAA	21189
P02788	PLLEACEFLRK	21255	Q08116	LLNDLQANSLK	21221	P49368	RQGGAPDAGQE	21189
Q93038	EDLRSRLQRGP	21255	Q15542	LVLAAGAYSPQ	21221	P23435	STFSGFLVFPL	21188
P19397	QIDKTSQTIGL	21254	P14652	STLCAIDLQFP	21220	P17858	TRRTLSMDKGF	21188
Q07654	FKPLQEAECTF	21254	P17082	KDKKGCHCVIF	21219	O14782	SLRPATVADHE	21188
P51157	NPPRSSMCAVQ	21254	P56192	PPEAPKGKKKK	21219	Q00577	LLQGEEEGEED	21188
O00287	QRQQQFPGTSM	21254	Q10587	VSKYETKYGPL	21219	P13984	YRHYQGEEKSD	21188
P04844	LAQQAVKRTAH	21254	P46939	CPNVPSRPQAM	21219	Q92785	KASIYQNQNSS	21187
P20062	GETIELRLVSW	21254	Q14147	TATEAETTRTR	21218	Q13643	VLCQGCGSQAGP	21187
O15164	LKSIEERQLLK	21254	Q13651	LPLISSLQSSE	21217	P17032	INVVNEGNYSG	21187
O75144	ALVCPSVPGAT	21254	O14817	CQVVKADTYCA	21217	P35680	SSSKQCPLQAW	21186
Q9UIY3	LFGIESKSSDS	21254	Q13702	CRRSSMKPGFV	21216	P78411	PYELKKGMSDI	21186
P00492	ISETGKAKYKA	21253	O15258	GKEDAGKAFAS	21216	P51610	SAPKKSKADGQ	21185
Q15543	RKAFDEANYGS	21253	Q06587	ELCYAPTKDPK	21216	P78413	LPKAGGKPFCA	21185
Q13360	KRIHNGQKLHE	21253	O96004	PQQVWALELNQ	21215	Q99708	IFSPKGKEQKT	21185
P40189	QTVRQGGYMPQ	21252	P54727	NFLLQQNFDED	21215	Q00839	PWSQHYHQGYY	21185
Q16594	DDDDDDDYDNL	21252	Q9P2Y4	PGEAGLGGQER	21215	O00194	GEKPPEKKCIC	21184

ID: ID from Uniprot Database (http://www.uniprot.org/) **Sequence**: Listed sequences match the Uniprot ID, but for the array experiments and for the computational predictions, the cysteines were changed to serine. **NA**: not available. **BLU**: biochemical light unit.

Table S1: Binding data from CAL peptide array.

ID	Sequence	BLU	ID	Sequence	BLU	ID	Sequence	BLU
P46783	GFGRGRGQPPQ	21184	Q92781	WVLPKPAQAVY	21146	P18827	QKPTKQEEFYA	21105
Q07960	ELFPSPDPSGL	21183	Q9UGU5	LDNIAYIMPGL	21145	Q9Y5J8	MKRVQQSSGPA	21104
O75190	GKEQLLRLDNK	21182	Q02877	YKEETIEKMQE	21145	O43670	RPPVMSQGGRY	21104
P20339	TQPTRNQCCSN	21182	P42677	TEGCSFRRKQH	21145	Q05084	KTDKEHELLNA	21103
O60895	VWRSKDSEAQA	21182	P31321	QRYNSFISLTV	21144	P29622	FLGKVVDPTKP	21103
P49241	DGYEPPVQESV	21182	Q06609	INADGVGDAKD	21144	Q16566	VPQQDVILPEY	21103
P37243	LPPLFLEVFED	21182	P29459	IDRVTSYLNAS	21143	P15621	ERTHTGEKPYE	21103
P35789	RHEIIHTGEKP	21182	P10644	QQYNSFVSLSV	21143	O15550	TLAPPLPSASS	21101
P08729	TASASRRSARD	21181	P05129	SPTSPVPVPVM	21143	NA	FGQGTRLQIKR	21100
Q92692	KGFVMSRAMYV	21181	P11801	VDPGARMTALQ	21143	P50120	DQVCRQVFKKK	21100
O60809	RIFFGPVPCPN	21181	P05217	EFEEEAEEEVA	21143	P51790	ANQDPASIMFN	21099
P17040	NHQRTHTGEKP	21181	P06865	VGFCEQEFEQT	21142	Q99832	AGRGRGRGRPH	21099
P49406	IWKEIEASKRS	21180	Q92764	PICVPCPGGRF	21142	P49207	LKAQAQSQKAK	21098
P32754	NMETNGVVPGM	21179	P48775	DSSYFSSDESD	21142	P52740	CNLAQHKKIHT	21098
P04645	IRSMPEQTGEK	21179	P00450	TVLQNEDTKSG	21141	P25398	DVIEEYFKCKK	21097
P14866	KLCFSTAQHAS	21179	P48547	AGRKPLRGMSI	21141	O14593	FQSNLVPADPE	21096
P08779	SSSSFSQGQSS	21178	Q13557	FSGGTSLWQNI	21141	P50479	AVYPNAKVELV	21096
Q9Y496	KPETVIDSLLQ	21177	Q04864	SDSFPYEFFQV	21141	Q9UPN9	LKSDERPVHIK	21096
Q92930	HASSDVERMIL	21177	Q04695	SSREQVHQTTR	21140	Q14146	AKHEGEKRYTA	21096
O96020	PKSTEKPPGKH	21175	Q9Y3U8	AAMRKAAAKKD	21140	P42575	HLYLFPGHPPT	21095
Q01344	PGVETLEDSVF	21175	P50990	GKKDWDDDQND	21140	O60894	VWQSKRTEGIV	21095
O43345	KKIHAGEKLYK	21175	P02753	YCDGRSERNLL	21139	Q9Y5W7	QKEVTSVTSWM	21095
Q01813	GQLEHVQPWSV	21174	P81605	AAQGENAGEDP	21139	Q03518	AMVQAPADAPE	21095
P22557	NMGPQYVTTYA	21173	P26639	EFRSKQAEEEF	21139	Q9Y2P7	NLLKHQNVHKG	21095
P30654	GSSSFSQGQCS	21172	Q14202	ELGRPGEEDLD	21139	Q13516	GSLPRLTSDAK	21094
Q9UDV7	PRQLPPPPERD	21172	Q9Y624	GEFKQTSSFLV	21138	P42771	DAAEGPSDIPD	21093
P14653	SPEASPSSVTS	21171	P01603	FGQGTKVDLKR	21138	Q92813	RHWLEKNFSKR	21093
P04090	GCTKRSLARFC	21171	P98168	LITVTGSSFLV	21138	P22750	AQAVAPQPCGC	21093
Q92784	YIWMEKRHRGP	21171	P36980	GKLVYPSCEEK	21137	P38570	LKSENLLEEEN	21092
Q14585	GKKLCELETIN	21171	P26447	EGFPDKQPRKK	21137	Q9Y337	KWIQETIQANS	21092
P17658	AYAEKRMLTEV	21170	P40198	CRMDHKAEVAS	21136	Q92545	PWSNSHFPHEN	21092
P51797	QARLRQHYQTI	21170	P14678	PPPPGMRPPRP	21136	Q9UMY4	DRNYVPGKVRQ	21092
P42701	LEDGDRCKAKM	21170	Q30201	AMGHYVLAERE	21134	P35713	SAVYYSACISG	21092
O43167	PQPTPLGQEQS	21170	Q9UKX5	PGLDPTPKVLE	21134	P01584	ITDFTMQFVSS	21091
P05156	VGRPFISQYNV	21169	Q92804	DYRNDQRNRPY	21134	P24723	NFSYVSPELQP	21091
P57737	SFEGVDEDEWD	21169	P46781	GAGAGDDEEED	21134	P30622	WATNCNDDETF	21091
Q15973	TGEKPYKCKKM	21169	P52736	AGQPSDSLYSL	21134	P10265	NQEREGIGYPF	21091
P35275	VINMVKPLHNS	21169	Q14586	AHQRSHTREKL	21134	NA	FGEGTRLQIKR	21090
P54257	GLGPSHLDMNY	21168	Q13976	PDDNSGWDIDF	21133	Q9UJU2	GTGPRMTAAYI	21090
P28068	GSNYSEGWHIS	21168	O95521	FIGPTPCPSCG	21133	O15211	ATGRKIARALF	21090
P17010	IMRHHKEVGLP	21168	P46060	RHSLLQTLYKV	21132	P51532	EDRSGSGSEED	21090
Q92833	IYIYFFVIIIF	21167	P14798	LTYCFNKPEDK	21131	O95201	GALATPPPAPT	21090
O76095	LEKVRKQIESI	21167	Q13123	ADGVEVKRPKY	21130	P05386	SDDDMGFGLFD	21089
P32929	KAAHPPSGIHS	21166	Q14258	AGATLSICSPK	21130	P48729	TDKSKSNMKGF	21088
P35610	RPRSWTCRYVF	21166	P17075	GVEVEVTIADA	21129	P35326	PCQSKYPPKSK	21088
Q99867	YQDATAEGEGV	21165	Q02985	REGIVEYPRCE	21128	O14628	SHLTVHESIHT	21088
Q9Y2Q1	NHSSNLTKHNS	21165	P11597	HLLVDFLQSLS	21127	P48230	CCGCCGGDGPV	21087
Q06730	GESPDDILNVQ	21165	P20839	HGLHSYEKRLY	21127	P57730	DPQLASKMGLH	21086
O15379	HDNDKESDVEI	21164	P35239	SQQNKSQCCSN	21125	P20338	TQAPNAQECGC	21086
Q14584	ERRHADERLSA	21164	P43405	RLRNYYYDVVN	21124	P28751	RKRRKMRQRSK	21086
P17025	VHQRTHTGEKP	21164	P20810	TTEETSKPKDD	21123	P52747	IQQGETPGLDD	21085
O75312	EGYEAGLAPQR	21164	Q9Y6T7	LVKRTRNRSKE	21123	Q12894	SRVRDKRADIL	21084
O95300	PQHVWALELKQ	21163	P26045	EEGLVQMLDPS	21123	Q16774	EEIKKAQRTGA	21084
Q12999	KDPRANPSAFL	21163	P12018	MEPTAARTRVP	21122	P46013	TTRSHRDSEDI	21084
Q9Y3E5	IDKVTGHLKLY	21163	P51572	AVDGPMDKKEE	21121	P09661	EMEEDTVTNGS	21084
P17036	QHQRIHTGENP	21163	P52198	HKDRAKSCNLM	21121	P40227	IMRAGMSSLKG	21084
P78406	AAEELKPRNKK	21162	Q9Y6Z2	LFQHWEGSIPT	21120	P07288	KWIKDTIVANP	21083
P07998	VHFDASVEDST	21162	Q9UL58	KHQKLHTRDKS	21120	Q05655	VNPKFEHLLED	21083
P05114	DEAGEKEAKSD	21161	Q15928	SHLNKHKKIHT	21118	P30050	INSGAVECPAS	21082
O95602	TGLFELKQPLR	21161	P52746	APAAPHTGPEG	21118	O15127	SSAAQGAFQGN	21082
P51957	QLKFFEENMNF	21161	P09455	VVCKQVFKKVQ	21117	P50053	KCGLQGFDGIV	21081
P49756	ETEAKKIGLVK	21160	P31350	TENSFTLDADF	21117	P52434	RVYLLMKKLAF	21081
O15067	NARNWTLEGSC	21159	P15692	ERTCRCDKPRR	21116	O95409	GLSSNFNEWYV	21081
P39019	AGQVAAANKKH	21159	P35274	ECNQCGKAFAQ	21116	Q16880	NGHIKHEKKVK	21080
P46777	FLRAQERAAES	21158	P47804	SPQKREKDRTK	21115	O15066	LYPQSRGLVPK	21080
Q13106	SLIKHQRIHSR	21158	O43566	HGSALHALSVP	21115	O15492	SSCSLDEPSHT	21080
Q9UQL6	AEEPMEQEPAL	21157	Q92766	ADGASQHVGME	21115	P51508	SVLSMHRNIHT	21080
P09601	VATVAVGLYAM	21157	Q9UNY5	RRVHARKEPSH	21115	Q15942	CRKCHTARAQT	21080
P20151	KWIKDTIAANP	21157	P51959	THLPTIPEMVP	21114	P08514	PPLEEDDEEGE	21079
P52739	NEDRTALPVLE	21157	Q03426	DSRVQQALDGL	21113	P01596	FGPGTKVDIKR	21079
Q13105	PTAPECPPPAE	21157	P52272	REIDVRIDRNA	21113	O43531	LSQNKVGSQNY	21079
O15535	HQKIHTVAELV	21157	P23921	ENRDECLMCGS	21112	P10276	SNRSSPATHSP	21079
Q14721	GAHGSTRDQSI	21156	P33763	AYNDFFLEDNK	21112	P10826	NSGVSQSPLVQ	21078
P78330	DFVELLGELEE	21156	O75820	QHQKLHTAWMQ	21112	P17041	ANLVTHQRIHT	21077
Q16384	EEISDPEEDDE	21156	Q05823	GGASGLASPGC	21111	Q01628	VIPVLIFQAYG	21076
Q14590	PFKCEECGKEF	21156	Q9UBX7	VDWIQETMKNN	21110	P07910	GEDDRDSANGG	21076
P49752	RDKGLVNRGRG	21155	P13631	GQGKGGLKSPA	21109	P51449	PGGWSPPALWK	21076
P54819	KATCKDLVMFI	21154	P51523	NHQRTHTVKKS	21109	Q14002	MIGVLAGMALI	21075
P51606	PAPTPACRGAE	21154	P05388	SDEDMGFGLFD	21108	Q92973	LKERLAAFYGV	21075
Q13398	QHQRVHIGEKP	21154	P21579	EEVDAMLAVKK	21108	P78368	RRKRKSLQRHK	21074
O95125	RHQRTHSEKTS	21153	Q08334	LGTPPGQGPQS	21107	P10113	KKPKKKSCLLL	21074
P51788	GSPSDSDDKCQ	21152	O76013	SSREHVQSRPL	21106	P10660	ASTSKSESSQK	21074
O76011	PCGTSQKGCCN	21151	Q15459	LALKERGGRKK	21106	P08865	TEWVGATTDWS	21074
P05787	LVSESSDVLPK	21151	O00268	HSLLLYKAFLK	21106	O60225	EEISDPPEDDE	21074
Q9UQR1	SPDATTGQTFG	21150	Q15131	TSEGQSKRCKP	21105	P22090	DKRLATKQSSG	21073
P01765	WGEGTLVTVSS	21149	Q06033	VHTDYIVPNLF	21105	Q9UIJ7	PQRSQKASVTP	21072
Q16552	TCVTPIVHHVA	21149	P04808	GCTKRSLAKYC	21105	Q92753	LFNPDCATACK	21072
P51814	HSGEKRYKASD	21149	O15514	DDIQTKRSFQY	21105	Q9UNL2	GLIALLSTGSK	21072

ID: ID from Uniprot Database (http://www.uniprot.org/) **Sequence**: Listed sequences match the Uniprot ID, but for the array experiments and for the computational predictions, the cysteines were changed to serine. **NA**: not available. **BLU**: biochemical light unit.

Table S1: Binding data from CAL peptide array.

ID	Sequence	BLU	ID	Sequence	BLU	ID	Sequence	BLU
Q03721	WGFPKHKDVPL	21070	P31948	KLMDVGLIAIR	21032	P20042	GKRAQLRAKAN	20985
P07307	EKRRNATGEVA	21070	P22614	HFRPAGLPEKY	21032	O95218	QKRNNVLKFTS	20985
P09651	SSSSYGSGRRF	21070	Q9UDV6	ALEPGRPNGLL	21032	P46695	STFLQQHPAAF	20984
P10398	ACLLSAARLVP	21069	P01607	FGQGTKLQITR	21031	P05198	DAEEMEAKAED	20984
Q03252	PRTTSRGCYVM	21069	P18615	DVYKENLVDGF	21031	P27352	HEHITANFTQY	20984
P17034	QHQRVHTGERP	21069	Q9Y584	AFSAAIDYYLR	21030	Q04759	FMNPRMERLIS	20983
P46063	MPDMNVTKFSN	21068	P02433	PNARLRSEENE	21030	P08621	ENGYLMEAAPE	20983
P10155	DVIRNFTLDMI	21068	P04430	FGQGTKVQIKR	21028	O60417	DSFLKAVPSQK	20983
P52435	VAIKDKQEGIE	21068	P08910	SDTEQVEADLE	21027	P35711	DSENHIAGQAN	20983
Q13554	HCSGAPVAPLQ	21067	Q9Y2P8	IGFSNLSKTLK	21027	Q14142	TTRPASSPSTT	20983
P78563	PTEQDQFSLTP	21067	P25815	CHKYFEKAGLK	21026	P05112	IMREKYSKCSS	20982
Q92953	SLQERGGASLK	21066	P42766	LYPLRKYAVKA	21025	P01601	TYYCQQYNSYP	20982
P12268	HSLHSYEKRLF	21066	P22626	GSGGYGGRSRY	21025	Q04912	PRPLSEPPRPT	20982
P09683	GAAAEGTLRPR	21066	P10301	KKGGGCPCVLL	21025	P25111	GGDAPAAGEDA	20982
P03952	SDGKAQMQSPA	21064	Q06731	QRTHIGEKPYE	21024	P17097	RLTQHQKIHMG	20981
P38663	KVSAPRVGGKR	21064	Q13568	GPWPMHPAGMQ	21023	P33176	VAVRGGGGKQV	20979
O95416	DPYSSAHATAM	21064	Q92504	VIMMVLIAHLE	21023	Q9P0G3	RSWIEETMRDK	20979
P09382	GDFKIKCVAFD	21063	P13598	AWRRLPQAFRP	21022	O43623	KHEESGCCVAH	20979
Q16666	SSMETSPDFFF	21062	P47813	IGDDDEDIDDI	21022	Q9Y2S0	DQKASRNESTF	20978
O15160	FLDELDAVQMD	21062	P17024	REHERTHTINR	21022	P52433	SLMDDYLGLVS	20978
P09132	GSKKGKGKKKK	21062	P51687	AWHRVHVYVSP	21021	O75437	THTGEKSYKYE	20978
P98182	THSACKTRKQK	21062	Q9UKR0	VDWIRMIMRNN	21020	Q13591	YFTDLNNYDEY	20977
P05113	GVMNTEWIIES	21061	Q15746	EGEGEGEEEEE	21019	Q9ULJ3	FSLWSHEQTHN	20977
P23396	PPAMPQPVPTA	21061	P46776	KSVGGACVLVA	21019	P41584	DWGVDELIITD	20976
P17081	GSRCINCCLIT	21061	Q14593	RHKRNHMGEKS	21019	P23297	VACNNFFWENS	20976
Q01826	GNTDINTDLKD	21061	Q03936	LSQLTQHKKIH	21019	P49458	KEARNVTMETE	20976
O15231	IHCGKCYEKLF	21061	O95721	IKSTERKVRQL	21018	O00458	RDKRADVGEFF	20975
Q14005	QSKETTAAGDS	21060	P17038	LTTPQTFSNIK	21018	P35712	ARLSKIHLEKY	20975
O00629	SANVPTEGFQF	21060	P18510	MVTKFYFQEDE	21016	Q14641	CDDGTSVKLCT	20974
P10516	SDSGVQPPASS	21059	Q14624	GVEISCWSVEL	21015	P14793	TNNLRPKKKVK	20974
P17017	RHERTHNAEKP	21059	O75897	TDTRLTFHFQF	21015	P35325	PCQPKYPPKSK	20973
P17020	QHQGVHTGDKP	21059	P51504	QSKIHSGGKNL	21015	P26951	LVTEVQVVQKT	20971
Q14003	LNANAAAWISP	21058	P08526	KNKWFFQKLRF	21014	Q9UIC8	GNELGLKEITY	20971
Q9Y6K8	QLCTAIDSIIF	21058	P56279	EDMLLELLPDD	21013	P14778	KLQREAHVPLG	20970
P29375	KLPMEDLKETS	21058	O43896	APDLKESGAAV	21012	Q9Y5Q6	GCSMTDLSALC	20970
O94810	ACGPGGGDGVA	21058	P05060	QKIAEKFSQRG	21012	P01600	FGQGTRVEIKR	20969
P23942	GAGAGQAPEAG	21057	P00540	VDLTSLKAELG	21011	P98155	ISVVSTDDDLA	20969
P46782	DELERVAKSNR	21057	Q13243	RSRSRSVDSGN	21011	P17022	HHEKIHTGEKP	20969
P80098	HLDKKTQTPKL	21057	P24394	VSVGPTYMRVS	21010	O00505	TANLQTKEFNF	20967
Q9UK12	DIILSLFLNDI	21057	Q9Y5W9	DPGQLETVLEK	21010	O75845	KLFNGEFTKTE	20966
Q02156	GFSYFGEDLMP	21056	P80162	QKILDSGNKKN	21009	Q9UHB9	GYIKGIFGFRS	20966
P12947	KNLQTVNVDEN	21056	P24278	TQENVDTILVE	21009	Q00537	HGKNRRQSMLF	20965
Q14192	DILCPDCGKDI	21056	P23743	PRSTNFFGFLS	21008	P01602	TYYCQQYNSYS	20964
P09913	LIPSASSWNGE	21055	P52333	PEGKHHSLSFS	21007	O95475	SITSSDSECDI	20964
P57727	IHEQMERDLKT	21054	Q9Y259	KGQLTSVHSSS	21007	Q15306	HRSIRHSSIQE	20963
P39023	KKDRIAKEEGA	21053	Q92876	TNWIQKTIQAK	21005	O60333	LSRRCPSQSKY	20963
Q03395	EAPPKEDLSEA	21053	P01611	FGGGTTVDIKR	21005	O95650	PAEKSLSLGTQ	20963
P51793	ANQDPESIMFN	21052	O95235	LLKSGPFGKKY	21005	P01616	FGGGTNVEIKR	20963
O60481	GLPPNFNEWYV	21052	P39028	LYKGKKERPRS	21004	O95751	DDDDEEEEDDY	20963
P49019	SLEKQLGCCIE	21050	Q9Y3T9	ELEDLQLSEDD	21004	P36578	KPTTEEKKPAA	20963
Q15437	HLKKLAVSSAC	21050	P42262	YNVYGIESVKI	21003	P22362	KMLRHCPSKRK	20963
P34991	VRKENQWCEEK	21050	P52597	YSGQNSMGGYD	21003	P01597	FGPGTKVEMTR	20962
Q03923	KIIHTGEKLQI	21050	O43264	ERRAAALAKIK	21003	Q99963	NYVEVIVPLPQ	20962
Q00536	RPAFRVVDTEF	21049	Q04984	RDGDILGKYVD	21002	P41743	NPLLMSAEECV	20961
P52436	EKLLNYAPLEK	21049	P09896	DKKRKGQVIQF	21002	P01609	FGQGTRVENKG	20961
P17540	VPPPLPQFGKK	21048	Q16587	RNFSLGSKPRN	21001	Q05952	TKTRSSGWKSN	20961
P57682	LALHRKRHMLV	21048	Q99543	EQVLNASRAKK	21001	P01608	FGGGTKVDFKR	20960
P35397	SEFEPAMQIDG	21048	O00410	QAAIQELLNSA	21000	P16632	ANVGAGKKPKE	20959
O43390	FYQDTYGQQWK	21048	P57071	VDVLRDHIHVH	21000	P09058	YLRKIKARKGK	20958
Q07002	GRGKNRRQSIF	21047	P05000	LRSKDRDLGSS	20998	Q07955	SPRHSRSRSRT	20958
P37108	TAATTAATAAQ	21047	Q08170	SRSRSRSHSRS	20998	P27144	KITPIQSKEAY	20957
O15503	AMGVPEKPHSD	21046	P51795	ANQDPDSILFN	20997	P05162	GFNMSSFKLKE	20957
P51815	RRREACLVSPN	21046	P17301	DEIDETTELSS	20997	Q99584	RKKKDLKIRKK	20956
P08700	AQQTTLSLAIF	21045	O15126	AAQNAFKGNQI	20997	P10914	LPSIQAIPCAP	20955
P23352	PHHYKPSPERY	21045	P47914	KRTQAPTKASE	20996	P08648	AQLKPPATSDA	20955
P12277	QAIDDLMPAQK	21045	P36954	TAPHCGHRWTE	20996	P09234	PTRPGMTRPDR	20955
P52294	QCEAPMEGFQL	21044	O00161	ANARAKKLIDS	20996	P13647	TTSSSRKSFKS	20954
P01308	CSLYQLENYCN	21044	O75346	LNVPPLLISIR	20996	Q07866	QQQQWPGRRHR	20954
P19387	HEIQSDVLTIN	21044	Q14974	TKELRKLKNQA	20995	O43615	DISASSTEQIL	20953
Q02546	YESSTASALVA	21044	Q92985	ECFLMELEQPA	20995	P11800	TVKDLISRLLQ	20953
O00124	VLILEEKEQTN	21043	P49908	NQAKKCECPSN	20995	Q16637	QKEGRCSHSLN	20953
Q14807	GLAAGQRCGAS	21040	Q13151	GGGGGYGGSSF	20994	P35243	PQKVKEKMKNA	20952
P51148	NPASRSQCCSN	21040	P55773	KLDTRIKTRKN	20994	P35908	AFGSSVTFSFR	20951
P17098	ASMLFDIREST	21040	O43736	EFIVETKICQE	20992	P09329	SVSYLFSHVPL	20950
P29376	PQNLWNPTYRS	21039	Q9Y5J6	AEQPGVSPSGS	20991	P49888	KESTLKFRTEI	20950
P12750	DKRLAAKQSSG	21039	P04271	TTACHEFFEHE	20991	P17019	KHKIIHTGEKP	20949
Q13571	GGPAPPPYSEV	21037	P32942	EAMGEEPSRAE	20990	O95838	TMEEILEESEI	20948
Q14587	MHQRTHVDDKH	21037	Q15323	PRCGPCNSFVR	20990	P06315	YYFCLQHDNFP	20948
Q02386	PSSEDSHRKTR	21037	P02383	GAAPRPPPKPM	20990	O95059	LSGNSRELVLD	20947
P31997	MIGVLARVALI	21036	Q9NZV5	GLRRGLPLLQP	20990	Q9NRM2	KQSSILNQLLL	20947
Q14532	MPCSPCPQGRY	21036	Q14190	LGASVIITNGR	20990	Q16816	AVLLSLAEEDY	20946
P11174	ATHSSRFIPLK	21035	P04643	AAGTKKQFQKF	20989	P39026	QKYDGIILPGK	20944
Q15835	SSSKSGMCLVS	21034	P17008	GARARYQKSYR	20989	P01613	FGVASKVESKR	20943
O14771	LPAGPSACAHR	21034	P05109	HKKSHEESHKE	20989	Q01650	QKLMQVVPQET	20943
P41091	GVTIKPTVDDD	21033	O00442	CQGIGMTNPNL	20988	Q01629	IIPVLVVQAQR	20942
Q13349	FSCVAPNVPLS	21033	O15370	PSSIADLVFTY	20988	O75600	VEVGRLHGALP	20942
P47736	ASEQHMPQLGC	21033	O14709	TNESKIEIQKI	20988	Q03938	SSTLATHKKIH	20941
Q13228	PGGDCSSDIWI	21033	P23821	DVNFEFPEFQL	20986	P52429	TSDQEDIKATE	20940

ID: ID from Uniprot Database (http://www.uniprot.org/) **Sequence**: Listed sequences match the Uniprot ID, but for the array experiments and for the computational predictions, the cysteines were changed to serine. **NA**: not available. **BLU**: biochemical light unit.

Table S1: Binding data from CAL peptide array.

ID	Sequence	BLU	ID	Sequence	BLU	ID	Sequence	BLU
P26440	VIGRAFNADFH	20938	P80697	YVDGVEVHNAK	20881	Q14141	QTLKRDKEKKN	20830
P17181	KTSEELQQDFV	20936	P10721	SSQPLLVHDDV	20880	P13866	TVAVFCHAYFA	20830
Q15477	DIVFAASLYTQ	20936	O14896	SMQLPPALPPQ	20879	P23152	SRSRSRSNERK	20826
Q13325	LTALCELRLSI	20935	O60259	DWIKKIIGSKG	20879	Q9NPC8	PMSANLVDLGS	20825
P05771	SYTNPEFVINV	20933	Q14563	THEFERAPRSV	20879	P12757	QILKSSKTAKE	20824
P06702	HHKPGLGEGTP	20932	Q02447	LVTVSGNETME	20879	P13624	NMKGMMGFNNM	20824
P07557	RSASEPSLHRT	20931	P19525	KSPEKNERHTC	20876	P57057	ATGDQVPFKEQ	20823
P06703	LALIYNEALKG	20930	P17023	TGEKPYSCKVC	20876	P18583	VYIVYLSDVFV	20823
P08578	DNITLLQSVSN	20929	P02545	RTQSPQNCSIM	20874	Q15811	KLTTDMDPSQQ	20822
P49588	FAQLRLGDVKN	20929	P46976	NIKRKLDTYLQ	20873	P04049	TLTTSPRLPVF	20822
Q14847	GMLPANYVEAI	20928	Q14525	SRCGPCNTFGY	20873	P00338	TLWGIQKELQF	20822
Q9Y5J9	RFAQIVQKGGQ	20926	P13010	GGDVDDLLDMI	20873	Q9NQX7	FVVETLICGVV	20821
P17014	VHYRTHSGEKP	20925	P35265	AKADGIVSKNF	20873	P46020	EFLPHSICAMQ	20821
O14879	RELLSNSEQLN	20924	P54920	KTIQGDEEDLR	20873	Q16719	TSILDSAETKN	20821
Q05481	ILANTVKPLLY	20924	P49862	KWINDTMKKHR	20872	O00338	EGTSINFCMEL	20820
O00186	PKDKVSLIKDE	20923	P04433	VYYCQQRSNWP	20872	P51170	QLTDTQMLDEL	20817
P52292	VQDGAPGTFNF	20922	O95613	QILLQRNPATR	20871	Q13247	SRSRSRSSSRD	20817
P01579	QMLFRGRRASQ	20922	Q15493	VKGIAPYSYAG	20869	O15041	HYRLPRHTLDS	20817
Q00532	TKKLNYRFPNI	20921	P36896	SQLSVQEDVKI	20867	P42704	QQLRKLRENSS	20816
P01598	FGQGTKVEVKG	20921	P17612	NEKCGKEFSEF	20866	O15532	VAAIKAALAQG	20812
Q9Y5W8	QTTQAPSLQKR	20920	Q08881	RQLAEIAESGL	20865	P46977	KDLDNRGLSRT	20812
P17016	AHQRGHTGEKP	20920	Q9UI43	QYHGRKGTVKQ	20865	P31749	PQFSYSASSTA	20811
P12956	LLEALTKHFQD	20918	P37088	ASSSTCPLGGP	20865	P20700	PRASNRSCAIM	20811
P06239	TATEGQYQPQP	20918	P43330	VVLRNPLIAGK	20865	Q9NSI8	VHKIIITEPSD	20811
P31949	FLKAVPSQKRT	20918	P22694	TEKCAKEFGEF	20864	P08842	DREKQDKRLSR	20811
Q13449	AASLLCLLSKC	20916	P07195	LWDIQKDLKDL	20864	P21583	LQEKEREFQEV	20809
Q15357	SIIMLEALERV	20916	Q13596	EAFLPEAKAIS	20864	P01286	LLQKHSRNSQG	20808
P28702	MEMLEAPHQLA	20916	Q04837	IFLSDQTKEKE	20863	Q14749	CYFIHVLKRTD	20806
P19013	IIGTTTLNKRR	20915	P49441	VQNLAPAETHT	20862	O14828	AGAAENAFRAP	20806
Q16629	PRRSASPERMD	20915	Q92503	SQSHSSSMISR	20862	P23327	ALADMLETPEP	20806
P55822	EGEEPGEDEDS	20915	Q16613	GHPFLRRNSGC	20861	Q9Y5W3	DHLALHMKRHM	20804
P13500	HLDKQTQTPKT	20915	P41225	AVNGTVPLTHI	20861	P08922	LTHSGYGDGSD	20804
O76009	ARCGPCNTFGY	20914	P26006	VTSWQTRDQYY	20860	P20663	PNSMVASPIEA	20803
Q04860	QTLAGHGGRRL	20914	P04264	FVSTTYSGVTR	20860	Q15491	STSTPFLSLPE	20803
Q13242	SPHYFSPFRPY	20913	P34896	SLFPLPGLPDF	20859	Q13573	HEHEGKKRRKE	20802
P41229	PCPQQPPQQQL	20913	P09912	HKYLDSEEDEE	20859	P21399	ILNYMIRKMAK	20801
O75362	NAHYRPNDKKT	20913	Q9Y287	NKFAVETLICS	20858	Q15726	AGRGWGAGAGQ	20801
P48048	SEVNETDDTKM	20912	Q01546	TTSSSQHSSTK	20858	P06312	VYYCQQYYSTP	20801
P06213	RILTLPRSNPS	20911	P14316	SDITQARVKSC	20856	Q14914	YIIEGFENMPR	20801
P06732	QSIDDMIPAQK	20910	P29508	SILFYGRFSSP	20856	P29353	LCLQQPVERKL	20801
P14618	FTNTMRVVPVP	20910	O94804	KFFPYSSADAS	20856	O00142	ILTPENRKHCP	20799
P31323	GTNMDIVEPTA	20909	Q13275	PRNRRHHPPDT	20854	O75711	KISFVIPCNNQ	20798
P52824	DRAPAPESDPR	20908	P01606	FGEGTRLEIKR	20853	Q9NZ72	RNKEQREEMSG	20797
Q9UET6	EMEDNEMSCSP	20905	Q14653	EGMDFQGPGES	20852	P81133	KGTSVIITNGS	20795
P13795	NQRATKMLGSG	20905	P53708	EQLTNDKTPEA	20852	P11277	ESLQPEPSHPY	20795
Q15356	EEEEEDGEMRE	20904	Q99747	EEEDEYSGGLC	20852	O43175	QHVTEAFQFHF	20794
O43761	TSPKGYQVPAY	20904	Q9UKQ9	YLDWIQEIMEN	20851	Q15531	LASSIGCTLGL	20794
O60220	SKPVFSESLSD	20903	P07306	LDKASQEPPLL	20851	Q01105	EEGEEDEGEDD	20792
P13861	GSSVDLGNLGQ	20903	P43331	MGRGNIFQKRR	20851	P12035	QSSQSSQRYSR	20790
P42263	YNVYGTESVKI	20902	Q9NRJ3	KHETYGHKTPY	20851	Q07890	LPLLENAETPQ	20790
P04183	PQQILQCSPAN	20902	P27930	PHHQDFQSYPK	20849	Q13277	ALIIGLSVGLN	20790
P38384	HIPINNIIVGG	20902	P23229	ITKWNRNESYS	20849	O15266	KARKHAEALGL	20789
P12755	AGSEGAAELEP	20902	Q02383	QYNEDRNPIST	20849	O75558	TLCCFCCPCLK	20789
Q9Y5J7	AAKAGLLGQPR	20900	P02724	VEIENPETSDQ	20848	P49619	FSLRRKSRSKD	20788
P15735	ITEDEAVLVLG	20900	P40305	SAIAAVIARFY	20848	P55735	ASVTEGQQNEQ	20788
Q05513	NPLLLSTEESV	20899	Q99828	PDFASSFKIVL	20848	P51688	SPQCQPLHNEL	20788
P34897	RAFPMPGFDEH	20898	O75838	PDFLSTFHIRI	20848	O95793	GNGPMSVCGRC	20788
O43187	CVGLEPPQDVT	20898	Q9UHI5	KDKDVAGQPQP	20848	O00515	QKSDSSLDAEV	20786
P31213	KSRKALIPFIF	20897	Q15831	KIRRLSACKQQ	20848	P56199	FKRPLKKKMEK	20784
P48551	DVDLGDGYIMR	20894	O15270	FDETTYEETED	20847	Q9Y5L5	LCCWCIKELLD	20782
Q12756	LSRRRSAQMRV	20894	P55855	DVFQQQTGGVY	20847	Q15005	HDSLAIERKIK	20781
P34168	ALLQIVEPAVH	20894	Q01130	KSPEEEGAVSS	20845	NA	FGQGTKLQIKR	20780
Q15532	YDQGQYGNYQQ	20893	O75368	KEAEVQAKQQA	20845	P18428	FLGANVQYMRV	20778
P51460	TQQDLLTLCPY	20892	O15131	QQEAPMDGFQL	20844	P51826	LHWLRNSAHLS	20777
Q13797	EDSWDWVQKNQ	20892	P01593	FGQGTKLEIKR	20844	P08294	KRRRESECKAA	20776
Q9NZV6	KGKETSASQGH	20892	P38391	MLHIWGKYTRS	20844	P51649	LELKYVCYGGL	20776
Q13813	YVEFTRSLFVN	20892	O60674	RVDQIRDNMAG	20843	P01042	SYYFDLTDGLS	20775
P12532	RIPTPVIHTKH	20891	P01604	FGQGTKLDIKR	20842	P46019	ELLPNSGCQMQ	20775
P38484	EKEQEDVLQTL	20890	P37023	SNSPEKPKVIQ	20841	O14497	ICDVLFLIGQS	20774
P30085	DEVVQIFDKEG	20890	P21127	SAAGPGFSLKF	20841	P01594	FGQGTKVEIKR	20773
P05127	NSEFLKPEVKS	20890	P04432	TFGGGTKVDNK	20841	P80511	LKAAHYHTHKE	20769
P57726	SEVGSMGALLF	20890	P01842	VEKTVAPTECS	20841	Q13435	GGSKKYKEFKF	20769
P09914	ADFENSVRQGP	20889	P31151	HGAAPCSGGSQ	20839	Q12872	AASKNLQTSAS	20768
P27448	IASKIANELKL	20889	Q06945	ESSISNLVFTY	20839	P48058	SGLAVIASDLP	20764
NA	FGQGTRLEIKR	20889	Q15404	ISRKPLAAKNR	20838	P35754	LTRLKQIGALQ	20764
P51168	VIESDSEGDAI	20888	O15235	GKYDCGHVQKK	20838	Q14746	AAKDQATAEQP	20763
P04279	HLNNDRNPLFT	20888	P13641	GRGRGRGGPRR	20837	P04278	QSPGNGTDASH	20763
P05455	QQKTENGAGDQ	20886	Q13094	QCTLTHAAGYP	20835	O43760	TTEGYQPPPVY	20761
P17021	SHQRVHTGERP	20886	Q14242	GDDLTLHSFLP	20835	O00570	GVNGTVPLTHI	20761
O43240	MSWINKVIRSN	20885	Q9NYY3	YALNMLLQRCN	20835	P41215	QIDDLYSIIKV	20760
P38935	QRTSRRKERGT	20885	O76014	VPSPCPVFLQD	20834	O14960	NCDSSDPTAYL	20759
P49223	KEKCEKFCKFT	20884	Q9UKR3	TQQQKWLKGPQ	20834	O75351	KKFTEDFGQEG	20759
Q16799	AKIPGAKRHAE	20883	P01617	FGQGTRLEIKR	20834	Q93100	KPNNDDPCLIS	20757
Q01892	DSALLPAVRRA	20883	O75638	AFNVMFSAPHI	20834	Q13683	GPDGHPGPGTA	20756
P48200	GLLNFVARKFS	20881	P19838	YGQEGPLEGKI	20833	Q13487	ELLGRAATPAR	20756
P13612	WSYINSKSNDD	20881	Q12840	LFPLHQETAAS	20833	P10147	QKYVSDLELSA	20756
P35790	AYFHQKRKLGV	20881	P30613	YTNIMRVLSIS	20833	Q16478	PAGPRELAEHE	20755
P18627	EPEPEPEPEQL	20881	P22612	NEKCAKEFSEF	20830	P23919	ATEKPLGELWK	20755

ID: ID from Uniprot Database (http://www.uniprot.org/) **Sequence**: Listed sequences match the Uniprot ID, but for the array experiments and for the computational predictions, the cysteines were changed to serine. **NA**: not available. **BLU**: biochemical light unit.
