# Supplementary material for: Computational Design of a PDZ Domain Peptide Inhibitor that Rescues CFTR Activity
Source: PLoS Comput Biol. 2012 Apr 19;8(4):e1002477. doi: 10.1371/journal.pcbi.1002477 (PMC3330111; doi:10.1371/journal.pcbi.1002477)
Supplement: Text S1 — Proof of Lemma 1 and 2. Additional methods detailing training of energy function weights and computational design of CAL motif residue positions. (PDF) [file pcbi.1002477.s002.pdf]

Supplementary Material: Computational Design of a PDZ Domain Peptide Inhibitor that Rescues CFTR Activity

Kyle E. Roberts[1], Patrick R. Cushing[2], Prisca Boisguerin[3], Dean R. Madden[2], and Bruce R. Donald[1,4,⋆]

[1] Department of Computer Science, Duke University, Durham, NC 27708, USA.
[2] Department of Biochemistry, Dartmouth Medical School, Hanover, NH 03755, USA.
[3] Institute for Medical Immunology, Charite Universitätsmedizin, 10115 Berlin, Germany
[4] Department of Biochemistry, Duke University Medical Center, Durham, NC 27708, USA.

The following is supplementary material for the following paper:

K.E. Roberts, P.R. Cushing, P. Boisguerin, D.R. Madden, and B.R. Donald. Computational Design of a PDZ Domain Peptide Inhibitor that Rescues CFTR Activity. *PLoS Computational Biology.* (2012).

Here we prove the two lemmas stated in the paper, give additional methods details and computational tests, and provide additional experimental data. Lemma 1 shows that the sequence search space can be accurately pruned in order to shorten the computational search and make it more tractable. Lemma 2 shows that the K^* algorithm has provable guarantees on the sequence scores that it finds. Additional information is provided for the training of the energy function weights. Table S1 gives the binding data for the 6223 peptides from the CAL peptide array used in the main text [1]. We also provide the starting template structural model of the CAL-CFTR complex.

S1 Extension of Provable Guarantees to Multiple Strands

The original proof of the K^* algorithm showed that it could find an ε-approximation to the K^* score (where ε is a user-defined parameter) [2]. In this context, a ε-approximation means $(1-\varepsilon)K^* \leq \tilde{K}^* \leq \frac{1}{1-\varepsilon}K^*$, where $\tilde{K}^*$ is the computed K^* score and K^* is the true K^* score. This proof in [2] relied on the assumption that the ligand was small enough that a complete partition function could be computed, which is generally true for enzyme active site designs but not for protein-peptide (PPI) or protein-protein designs. This assumption was used not only to prove the correctness of the intermutation pruning criterion, but also to compute an ε-approximation to the K^* score.

Having provable guarantees on the algorithm output ensures that any incorrect predictions are due to the input model and not the search algorithm; this cannot be shown for heuristic methods. Also, having provable guarantees can make it easier to accurately include experimental data into the computational model.

The goal of the current paper is to expand K^* to apply to protein-peptide and protein-protein interactions. In these cases it is not guaranteed that either member of the bound complex will be small enough to compute the complete partition function. Thus, we extend

⋆Corresponding author: Bruce R. Donald, brd+plos11@cs.duke.edu, Tel: 919-660-6583, Fax: 919-660-6519.

the previous proofs to handle complexes where both partners have approximate partition functions.

Intermutation Pruning. The idea behind intermutation pruning is that it is possible to provably show that, in some cases, a K^* score for a candidate sequence that is currently being computed will never be better than a K^* score for a sequence that has already been found. This pruning step significantly reduces the number of K^* scores that must be fully computed and increases the speed of the algorithm. The original proof can be found in [2]. Given that $K_i^* \geq \gamma K_0^*$, where K_i^* is the K^* score of the current sequence, K_0^* is the best score observed so far, and γ is a user-specified parameter defining the number of top scoring sequences we want an ε-approximation for, there exists an intermutation pruning criteria for PPI designs. In the following lemma, n is the number of conformations in the search yet to be enumerated, k is the number of conformations that have been pruned from the search with DEE, E_0 is the lower energy bound on all pruned conformations, R is the universal gas constant, and T is the temperature. The full partition function for the protein-protein complex, protein A, and protein B are q_{AB}, q_A, and q_B respectively, while q_{AB}^*, q_A^*, and q_B^* denote the current calculated value of the partition functions during the computational search.

Lemma 1. *If the lower bound E_t on the minimized energy of the $(m+1)^{th}$ conformation returned by A^* satisfies $E_t \geq -RT(\ln(\gamma\varepsilon K_0^* q_A^* q_B^* - k\exp(-E_0/RT)) - \ln n)$, then the partition function computation can be halted, with q_{AB}^* guaranteed to be an ε-approximation to the true partition function, q_{AB}, for a candidate sequence whose score K_i^* satisfies $K_i^* \geq \gamma K_0^*$.*

Proof. Using the previous intramutation pruning methods [2], we can compute ε-approximations for the partition functions of each protein. We have:

$$(1-\varepsilon)q_A \leq q_A^* \leq q_A$$

$$(1-\varepsilon)q_B \leq q_B^* \leq q_B \tag{S1}$$

Given $K_i^* \geq \gamma K_0^*$, which is by definition $\frac{q_{AB}(i)}{q_A(i)q_B(i)} \geq \gamma K_0^*$, (where i denotes that the partition function is for the ith sequence) we have that:

$$q_{AB}(i) \geq \gamma K_0^* q_A(i) q_B(i) \geq \gamma K_0^* q_A^*(i) q_B(i) \geq \gamma K_0^* q_A^*(i) q_B^*(i). \tag{S2}$$

Next, by definition $q = q^* + q' + p^*$ where q' is the partition function of the remaining conformations and p^* is the partition function of the pruned conformations, note that:

$$q' \leq n\exp(-E_t/RT) \tag{S3}$$

$$p^* \leq k\exp(-E_0/RT) \tag{S4}$$

If the following condition holds then the search can be stopped and we have an ε-approximation to the partition function $q_{AB}(i)$:

$$n\exp(-E_t/RT) + k\exp(-E_0/RT) \leq \varepsilon K_0^* \gamma q_A^*(i) q_B^*(i). \tag{S5}$$

To show this use eqs. (S3) and (S4) to show that

$$q' + p^* \leq \varepsilon K_0^* \gamma q_A^*(i) q_B^*(i) \tag{S6}$$

and by eq. (S2)

$$q' + p^* \leq \varepsilon q_{AB}(i) \tag{S7}$$

which by the definition of q implies

$$q_{AB}^*(i) \geq (1-\varepsilon) q_{AB}(i). \tag{S8}$$

This shows that when designing multiple strands there exists an intermutation pruning criterion that uses the stopping condition obtained from eq. (S5):

$$E_t \geq -RT(\ln(\gamma \varepsilon K_0^* q_A^* q_B^* - k \exp(-E_0/RT)) - \ln n) \tag{S9}$$

Thus, if the stopping criterion is met then q_{AB}^* is an ε-approximation to q_{AB}. □

K^* Score Approximation. The proof that an ε-approximation can be found for the K^* score also requires that the ligand partition function be fully computed [2]. In PPI designs the ligand partition function will not be fully computed so the K^* approximation will no longer be an ε-approximation but rather a $\sigma = \varepsilon(2-\varepsilon)$ approximation is obtained.
Lemma 2. *When amino acid substitutions (or flexible residues) are allowed on both strands in a computational design, the computed K^* score is a σ-approximation to the actual K^* score, where $\sigma = \varepsilon(2-\varepsilon)$.*

Proof. The full K^* score is denoted as $K^* = \frac{q_{AB}}{q_A q_B}$ and the computed K^* score as $\tilde{K}^* = \frac{q_{AB}^*}{q_A^* q_B^*}$. We can then bound $\tilde{K}^*$ as follows:

$$\max\left(\tilde{K}^*\right) = \max\left(\frac{q_{AB}^*}{q_A^* q_B^*}\right) = \frac{q_{AB}}{(1-\varepsilon)^2 q_B q_A} = \frac{1}{(1-\varepsilon)^2} K^* = \frac{1}{1-[\varepsilon(2-\varepsilon)]} K^*$$

$$\min\left(\tilde{K}^*\right) = \min\left(\frac{q_{AB}^*}{q_A^* q_B^*}\right) = \frac{(1-\varepsilon) q_{AB}}{q_B q_A} = (1-\varepsilon) K^*$$

Noting that $(1-\varepsilon) \geq 1 - [\varepsilon(2-\varepsilon)]$, $\tilde{K}^*$ is bounded by

$$(1-\varepsilon(2-\varepsilon)) K^* \leq \tilde{K}^* \leq \frac{1}{1-\varepsilon(2-\varepsilon)} K^* \tag{S10}$$

Which shows that given ε-approximations for all of the partition functions, we have a $\sigma = \varepsilon(2-\varepsilon)$ approximation for the computed K^* value. □

The two lemmas above provide provable guarantees for the K^* algorithm when allowing amino acid substitutions on multiple protein chains.

S2 Training of Energy Function Weights

To obtain accurate energetic predictions for the CAL-CFTR system, scaling parameters for the van der Waals, electrostatics, and solvation energy terms were determined. The best weights for each of these terms were found by training with 16 previously-determined experimental K_i values for the CAL-CFTR system [1]. A gradient descent method was used to determine the optimal energy weights. The initial weights used for the search were vdW: 0.7, dielectric: 20, solvation: 0.7. For each iteration of the search one energy weight parameter was varied, and a K^* score was computed for each sequence that had a known experimentally-measured K_i. The Pearson correlation of K^* score vs. $1/K_i$ was calculated and the weights with the best correlation were used for the next iteration. Each parameter was varied 8 times and the amount it varied was reduced on each iteration.

The best correlation found through the parameter search that maintains reasonable K^* scores is shown in Fig. S1. The correlations over the entire parameter search space range from 0.0 to 0.75, which highlights the importance of choosing the correct weighting factors. The parameters chosen for the design runs are as follows: a van der Waals scaling of 0.9, a dielectric constant of 20, and a solvation scaling of 0.76. These parameters are reasonable and similar to parameters used in previous designs. Since the peptide design occurs at the surface of the protein, this necessitates the somewhat high dielectric constant.

S3 Recapitulation of CAL Motif at Positions 0 and -2 of the Design Peptide

Comparison of K^* scores against the HumLib array data already suggests that K^* is able to enrich for sequences that bind CAL. However, since the energy function was trained on peptide sequences that all matched the CAL binding motif, it is important to determine whether K^* allows false positives at peptide positions 0 and -2. However, except for the HumLib array, no additional CAL binding data exists for non-motif residues at positions 0 and -2. Therefore to do additional computational tests we must make the assumption that if the peptide sequence matches the CAL motif it can bind CAL, and if it does not match the motif it does not bind CAL. The HumLib data generally support this assumption, although there are some peptide sequences that can bind CAL but do not match the motif (10 out of 5867 sequences).

A K^* design search was conducted where positions 0 and -2 were mutated to all amino acids (except Pro) while keeping positions -1 and -3 fixed to Arg and Val respectively. The resulting ROC curve (Fig. S2) has an AUC = 0.94 which shows that K^* has the ability to recover the known CAL binding motif.

References

1. Cushing PR, Fellows A, Villone D, Boisgurin P, Madden DR (2008) The relative binding affinities of PDZ partners for CFTR: a biochemical basis for efficient endocytic recycling. Biochemistry 47: 10084–10098.

2. Georgiev I, Lilien RH, Donald BR (2008) The minimized dead-end elimination criterion and its application to protein redesign in a hybrid scoring and search algorithm for computing partition functions over molecular ensembles. J Comput Chem 29: 1527–1542.

S4 Supplemental Figures

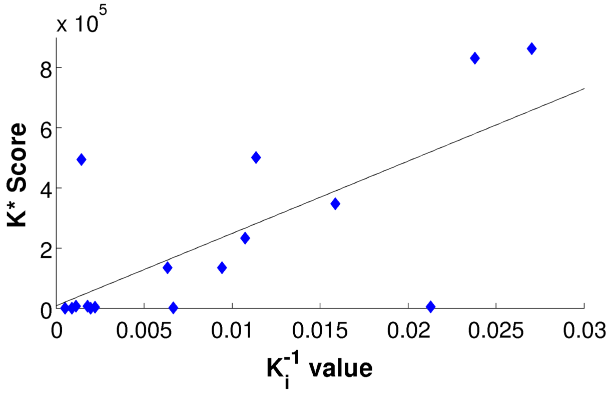

Fig. S1. Correlation between K^* score and experimental K_i^{-1} values for CAL PDZ peptide inhibitors. Pearson Correlation of 0.75.

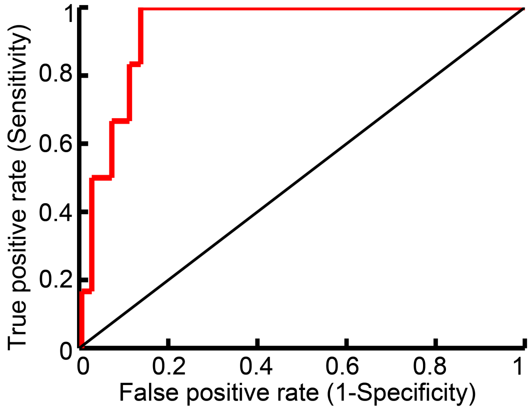

Fig. S2. A K^* design search was conducted where positions 0 and -2 were mutated to all amino acids (except Pro) while keeping positions -1 and -3 fixed to Arg and Val respectively. The resulting ROC curve has an AUC = 0.94.

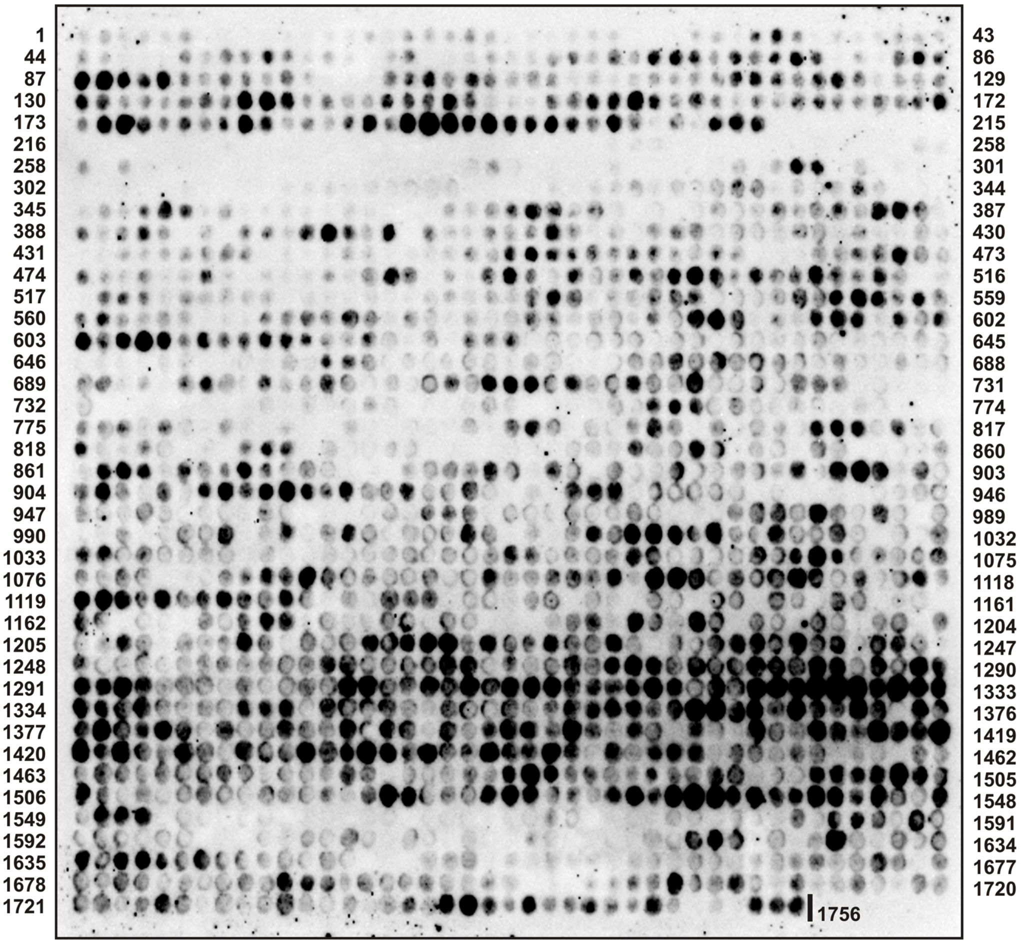

Fig. S3. CAL binding data for ProLib spot array. We selected the amino acid preferences of the last 4 C-terminal ligand residues based on their frequency deduced from the 6223-Humlib and the substitutional analysis. For the CAL profile library of the type $bbbbB_{-3}B_{-2}B_{-1}B_0$, we choose B_{-3} = A/C/D/E/F/I/K/L/M/N/Q/R/S/T/V/W/Y, B_{-2} = S/T, B_{-1} = A/C/D/E/F/I/K/L/M/N/ Q/R/S/T/V/W/Y, B_0 = I/L/V for position -3 through 0, respectively. Incubation condition: 10 μg/ml His-tagged CAL PDZ domain detected by anti-His (Sigma; 1:2600)/anti-mouse-HRP (Calbiochem; 1:2000) antibody sandwich.
